# Energy-Efficient Information Transfer by Visual Pathway Synapses

**DOI:** 10.1016/j.cub.2015.10.063

**Published:** 2015-12-21

**Authors:** Julia J. Harris, Renaud Jolivet, Elisabeth Engl, David Attwell

**Affiliations:** 1 Department of Neuroscience, Physiology and Pharmacology, University College London, Gower Street, London WC1E 6BT, UK

## Abstract

The architecture of computational devices is shaped by their energy consumption. Energetic constraints are used to design silicon-based computers but are poorly understood for neural computation. In the brain, most energy is used to reverse ion influxes generating excitatory postsynaptic currents (EPSCs) and action potentials. Thus, EPSCs should be small to minimize energy use, but not so small as to impair information transmission. We quantified information flow through the retinothalamic synapse in the visual pathway in brain slices, with cortical and inhibitory input to the postsynaptic cell blocked. Altering EPSC size with dynamic clamp, we found that a larger-than-normal EPSC increased information flow through the synapse. Thus, the evolutionarily selected EPSC size does not maximize retinal information flow to the cortex. By assessing the energy used on postsynaptic ion pumping and action potentials, we show that, instead, the EPSC size optimizes the ratio of retinal information transmitted to energy consumed. These data suggest maximization of information transmission per energy used as a synaptic design principle.

## Highlights

• How do synapses optimize energy consumption on information transmission?
• We find that increasing synaptic current size increases information transmission
• But larger or smaller EPSCs decrease the information transmitted per energy used
• Thus, the biologically observed EPSC size maximizes synapse energy efficiency

## Teaser

Synapses transmit information between neurons, but the energetic cost of postsynaptic currents is high. How are synaptic properties set to balance this trade-off? Harris et al. show that thalamic relay synapse properties are tuned to maximize not bits of information transmitted per second but bits of information transmitted per ATP molecule used.

## Introduction

The geometry of excitatory synapses is subject to competing constraints. Synapse diameter needs to be small, first so that a neuronal dendrite can receive a large number of synaptic inputs and second because if synapses are too large in diameter then glutamate clearance by diffusion to surrounding astrocytes will be too slow, limiting the maximum rate of information transfer through the synapse [1]. On the other hand, if synapses are too small and possess only a few glutamate receptors, then variability in the opening of postsynaptic ion channels creates noise in the postsynaptic signal [2, 3]. Similarly, because most brain energy is used to pump out ions that enter through postsynaptic receptors [4, 5], the number of receptors per synapse should be kept small to minimize energy use, but if it is too small, the postsynaptic effect of the input will be negligible. How have excitatory synapses evolved to cope with these competing requirements?

We studied the lateral geniculate nucleus relay synapse in the visual system to investigate whether postsynaptic currents are large, to transmit to the cortex as much as possible of the information arriving from the retina, or smaller, to save energy. Surprisingly, increasing synaptic conductance beyond the biological norm allows more information flow across the synapse, showing that synapse properties are not set to maximize information transfer. Instead, analysis of the energetic cost of the postsynaptic ion pumping associated with synaptic signaling [6] revealed that synapse properties are evolutionarily selected to maximize the information transferred per energy used. In other words, synapses do not maximize bits transmitted per second but bits transmitted per ATP molecule.

Theoretical analysis has previously shown that the low mean firing rate of neurons [7] and the surprisingly low release probability of central synapses [4] can be explained if axons and presynaptic terminals operate to maximize the information transmitted per energy used. The results presented below extend this concept to postsynaptic terminals, the largest consumers of energy in the brain, and are consistent with energy use profoundly constraining the operation of the CNS.

## Results

### Spike Transmission through the LGN Relay Synapse

**Figure 1:**
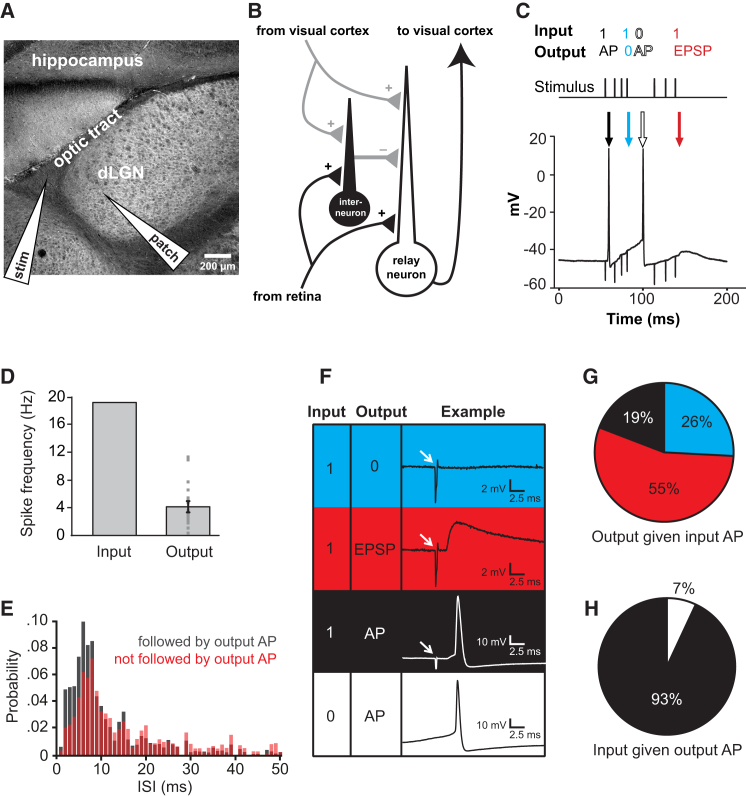
Spike Transmission through the Optic Tract-LGN Synapse (A) Slice preparation showing the stimulating electrode in the optic tract and the recording electrode in the dorsal LGN. (B) Circuitry of the LGN with functioning pathways in black (axons from the retina and to the visual cortex) and inactivated pathways in gray (cortex is removed to remove cortical input, and inhibition from interneurons is abolished with gabazine). (C) Specimen trace showing that the stream of input action potentials (timing shown by stimulus trace) does not reliably generate postsynaptic action potentials. (D) Spike frequency (mean ± SEM) in the input stimulus train (input) and evoked in 18 LGN cells (output). Points show data from individual cells. (E) For cases where a postsynaptic action potential did occur (black distribution) or did not occur (red distribution), graphs show the probability of the preceding two presynaptic APs being separated by the interval shown on the abscissa (the area under each distribution is unity). (F) Logical table stating possible input and output combinations, with specimen examples of each. (G) Observed outcomes given an input AP (EPSPs had to be larger than 1 mV to be counted). (H) Observed input APs given an output AP. See also Figure S1.

We stimulated optic tract axons making synapses onto whole-cell patch-clamped dorsal LGN relay neurons in the thalamus of rat brain slices [8] (Figures 1A and S1) with ganglion cell responses to natural visual scenes [9]. The relay neurons were held in the tonic firing mode (at −55 mV) seen during alert wakefulness in vivo, where a single input spike tends to produce (at the most) one output spike, as opposed to the burst mode (below −70 mV) occurring during less-alert states or sleep, where a single input spike may produce a burst of output spikes (see Supplemental Information and Figure S5E). To isolate a single excitatory input from the optic tract, we used animals at an age (P28) when the retinogeniculate connection is mature and one retinal ganglion cell makes a giant glomerular synapse with many release sites onto one LGN relay neuron [10, 11], we cut off the cortex, and we blocked GABA_A_ receptors (Figure 1B) although blocking inhibition had little effect (see Figure S1F and Supplemental Information). The stimulus trains used had a mean spike frequency of ∼19 Hz, which evoked postsynaptic firing at a mean rate of ∼4 Hz (Figures 1C and 1D). Thus, despite being designated a “relay synapse,” the optic tract to LGN synapse does not ensure an output spike for every input spike. Instead, as first described [12], the occurrence of two input action potentials close together in time increases the chance of generating a postsynaptic action potential (Figure 1E).

Spike transmission at this synapse has been observed to vary widely (even within the same species and anesthetic state) [12, 13, 14, 15, 16], but it is generally agreed that less than 50% of spikes are successfully transmitted (with the average across these papers being 23% ± 8%). We found that only 19% of input action potentials produced an output action potential (Figures 1F and 1G; see Experimental Procedures), whereas 55% produced only an EPSP and 26% produced no detectable EPSP in the following 18 ms (only EPSPs over 1 mV in amplitude were reliably detectable, so this may be an overestimate; see Experimental Procedures for choice of 18 ms). Some apparently spontaneous output action potentials were associated with no input stimulus spike in the preceding 18 ms (Figure 1H).

The input action potential train, which was composed of retinal ganglion cell responses to natural movies (from [9]) carried 94 bits/s of information (quantified using the direct method [17] but with zero noise entropy). The output spike train recorded in relay neurons carried roughly one-fifth of this information (18.3 bits/s). However, the number of bits of information encoded per action potential was not significantly affected by transmission through the synapse: the input train encoded 4.9 bits/spike (94 bits/s with a mean firing rate of 19.0 Hz), which is slightly higher than the 1.5–3.5 bits/spike found for natural stimuli in guinea pig retinal ganglion cells [18], whereas on average, the output train evoked by synaptic input encoded 4.7 bits/action potential (18.3 ± 4.5 bits/s at 3.9 ± 1.1 Hz), which is similar to a previous report of 3.6 bits/action potential in cat LGN cells [19].

### Relationship between Synaptic Conductance and Spike Output

How does the reduction in mean spike rate at the LGN synapse affect the amount of information transmitted, and how is information flow affected by the size of the postsynaptic conductance evoked by presynaptic glutamate release? Although not all retinal spikes are transmitted across the synapse, those that are relayed are more informative about the visual stimulus than those that fail to be transmitted [13, 14]. Does the relay neuron omit some spikes because they are less informative or because reliably transmitting them would require a larger excitatory postsynaptic current (EPSC) with a correspondingly larger energetic cost? We investigated this by altering the postsynaptic conductance evoked by glutamate release in order to increase or decrease the proportion of retinal spikes that are transmitted and examining the effects on information transmission and postsynaptic energy use.

**Figure 2:**
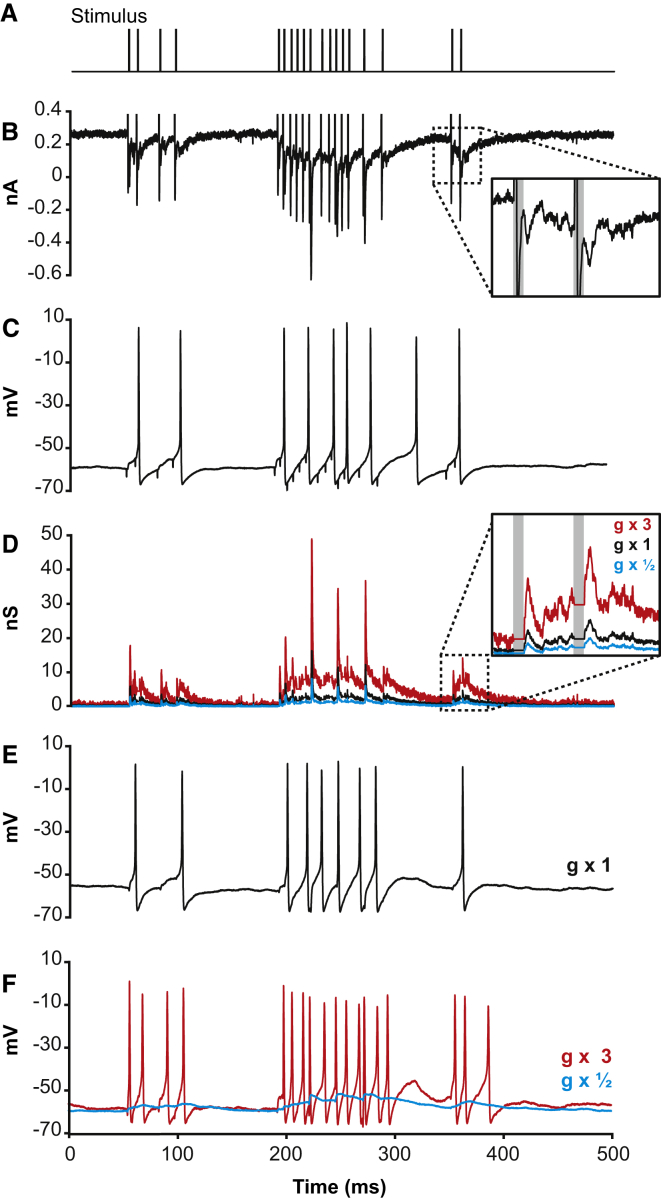
Conversion of Synaptic Conductance to Action Potentials in the LGN (A) A section of the stimulus train applied to the optic tract axon. (B) The EPSC train evoked in the LGN cell by the input train when voltage clamped at −55 mV. (C) The AP sequence evoked in current clamp mode by the train in (A). Large vertical deflections in (B) (gray in inset) and small downward deflections in (C) are stimulus artifacts. (D) The EPSC conductance time course derived from (B) for injection by dynamic clamp, with the same amplitude as evoked by synaptic input (g × 1) and scaled up and down in size (g × 3; g × ½). (E) AP stream evoked by dynamic clamp injection at the soma of the g × 1 conductance trace in (D). (F) AP stream evoked by dynamic clamp injection of the g × 3 and g × ½ conductance traces in (D). All data are from the same cell.

We recorded the sequence of EPSCs evoked by the input action potential train (Figures 2A and 2B) and examined (in current clamp mode) the resulting action potential train that these EPSCs generated (Figure 2C). After converting the EPSCs to conductance changes, we used dynamic clamp [20] to inject into the cell soma the recorded conductance scaled up or down by different factors (see Experimental Procedures), so that we could examine the voltage response that would be produced by a larger or smaller synaptic conductance. The dynamic clamp technique uses a computer interface to calculate how much current needs to be injected into the cell to mimic the synaptic conductance while the membrane potential is changing. Figure 2D shows the scaled conductance time course, derived from the current trace in Figure 2B after removal of the stimulus artifact (see Experimental Procedures), for a range of scaling factors. For a synaptic conductance time course injected by dynamic clamp at the soma, with a magnitude equal to that recorded in voltage clamp for a real synaptic input, the resulting action potential response (Figure 2E) was similar to that evoked by the real synaptic input to the cell (Figure 2C), with a similar mean firing rate (4.2 ± 1.0 Hz in dynamic clamp; 3.9 ± 1.1 Hz with synaptic input; ten cells; not significantly different; p = 0.75). Scaling the conductance time course evoked by the input signal up or down led to the recorded neuron generating more or fewer spikes, respectively (Figure 2F).

### Information Transmission

**Figure 3:**
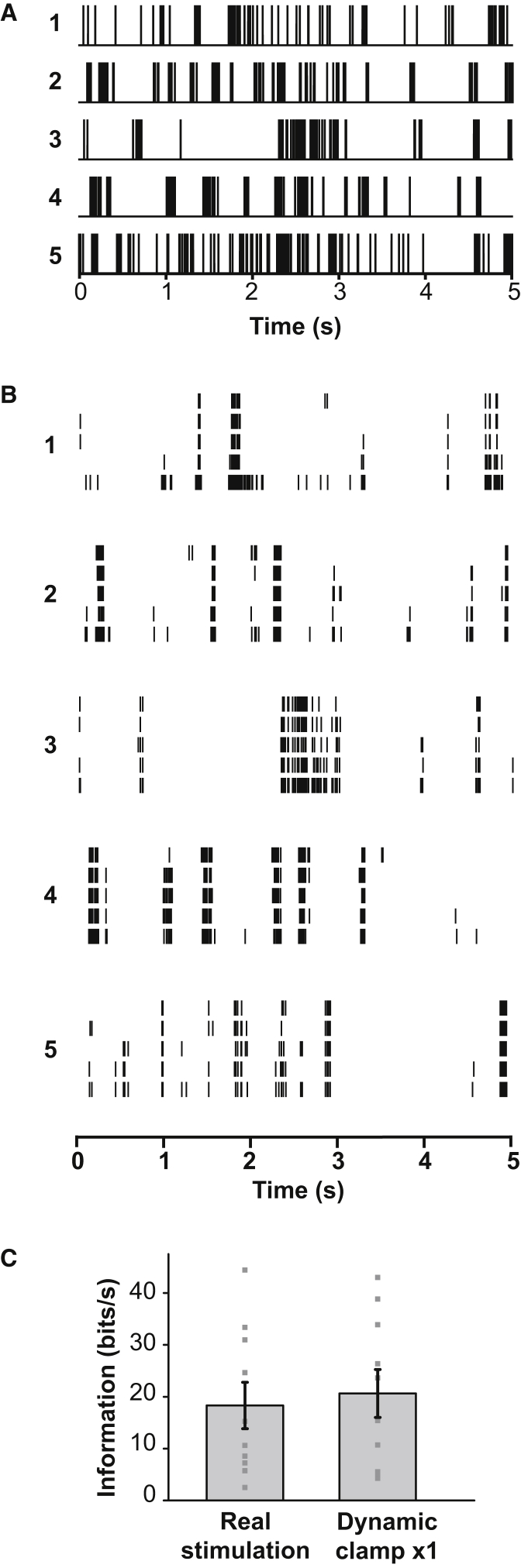
Information Conveyed to the LGN by Natural Scenes (A) Five-second segments of AP streams (1–5) recorded from ganglion cell axons in response to natural scenes [9]. (B) AP responses (each line is one AP) of a specimen cell to five separate applications of trains 1–5 to the optic tract (real stimulation). (C) Output information (mean ± SEM) in ten cells (shown as points) when the cell received AP-evoked synaptic currents (real stimulation) or had the measured conductance evoked by real stimulation injected at the soma with the same magnitude (dynamic clamp × 1). See also Figure S2.

To quantify the information transmitted across the synapse, we measured the mutual information between stimuli and responses using the direct method [17, 19]. Five different spike trains (1–5; Figure 3A) recorded in retinal ganglion cells in response to natural scenes [9] were used as the stimulus input. Optic tract axons were stimulated with these trains in sequence (1-2-3-4-5), five times (Figure S2A). Each relay neuron therefore responded to each train five times (responses are grouped by input train in Figure 3B). The relay neuron responses to the same input train were generally similar, showing that the output spike trains were not very noisy, whereas the responses to different trains were very different, showing that the output spike trains had the capacity for high variability and thus high information content. Mutual information—how much of the input information is transmitted to the output—is the information capacity minus the noise (see Supplemental Information and Figures S2B, S2C, and S3 for full calculations). With presynaptic stimulation, the mutual information (hereafter simply called “information”) was 18.3 ± 4.5 bits/s (Figure 3C). Employing dynamic clamp to apply the recorded synaptic conductance at the cell soma gave an output information rate of 20.6 ± 4.6 bits/s, which was not significantly different from that seen with normal synaptic input (p = 0.59; Figure 3C).

### Energy Use on Postsynaptic Current

**Figure 4:**
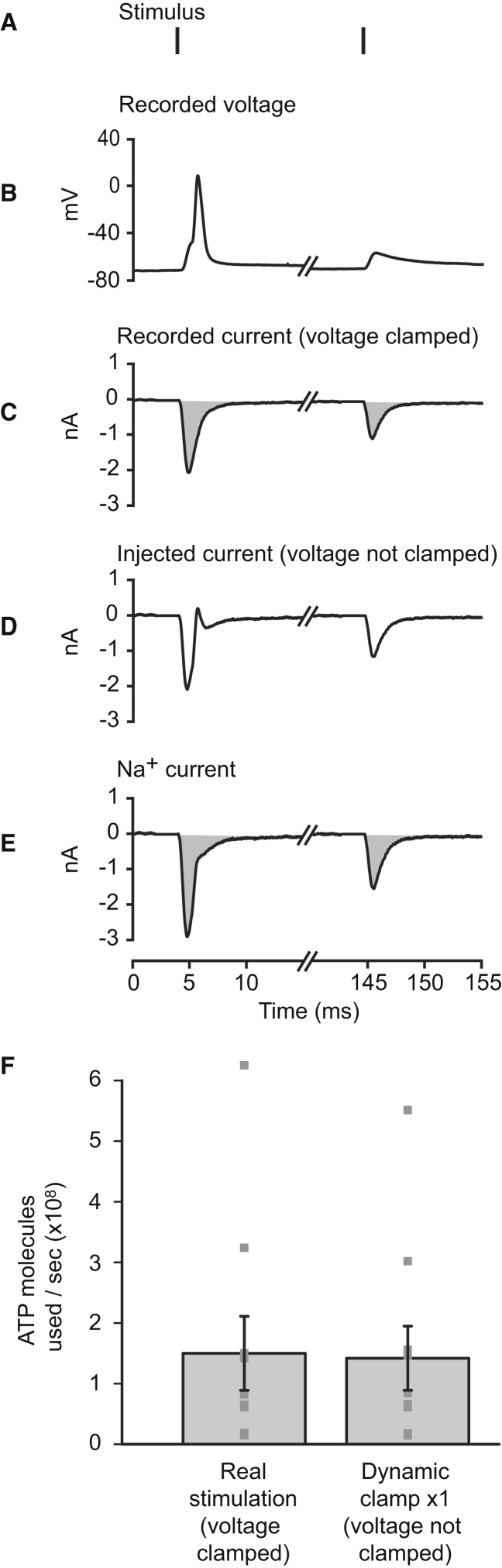
Energy Use on Postsynaptic Currents in the LGN (A and B) Two stimuli from one of the stimulus trains (A), chosen to evoke (B) an action potential or just an EPSC. (C) The EPSCs evoked by the stimuli recorded at −55 mV in voltage clamp. The action potential in response to the first stimulus (as shown in B) was evoked by a larger EPSC (note that, because current was recorded in voltage-clamp mode, it does not reflect the sodium influx associated with the action potential itself). Integrating the current trace (area shaded in gray) gives the total postsynaptic charge entry. The actual Na^+^ entry is 1.42 times larger than this (see Experimental Procedures). Na^+^ entry is then converted to ATP cost at a rate of one ATP molecule per three Na^+^ ions. (D) The synaptic current, calculated from the conductance derived from (C), that is injected in dynamic clamp (with a conductance scaling factor of 1). Because the membrane potential is not voltage clamped, the current shows an outward deflection as the action potential depolarizes the cell positive to the reversal potential for the synapse. (E) The Na^+^ current calculated to occur during dynamic clamping. (F) The ATP used on extruding Na^+^ entering through the postsynaptic conductance, calculated under voltage-clamp conditions during stimulation of the optic tract, and when injecting the same conductance at the soma using dynamic clamp (ten cells; mean ± SEM shown as bar; individual cells shown as points).

**Figure 5:**
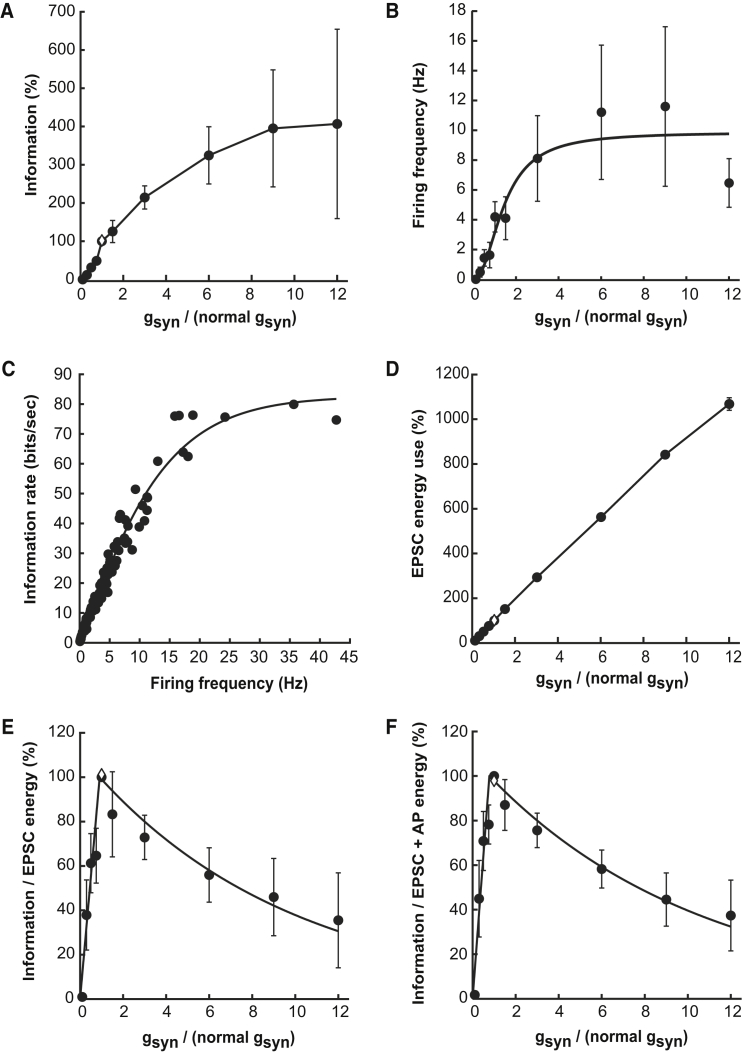
Postsynaptic Conductance Magnitude Maximizes Information Transferred per Energy Used at the RGC-LGN Synapse (A) Dependence of output information on synaptic conductance (g_syn_) magnitude, when cells were stimulated with dynamic clamp (black points) with g_syn_ × 1 (applied to all ten cells) and other values (six cells for g_syn_ × 0.1, seven cells for ×0.3, nine cells for ×0.5, three cells for ×0.75, three cells for ×1.5, eight cells for ×3, seven cells for ×6, seven cells for ×9, and four cells for ×12) or with optic tract stimulation (white diamond; ten cells). Information is normalized to the value with g_syn_ × 1, for which the mean information rate was 20.6 ± 4.6 bits/s. Symbols and number of cells per condition are the same in (A) and (D)–(F). (B) Relationship between firing frequency and g_syn_ for ten cells (fitted equation has the form F_max_.g_syn_^n^/(g_syn_^n^ + g_syn0.5_^n^), where F_max_ = 9.9 Hz, n = 2, and g_syn0.5_ = 1.4). (C) Dependence of information rate on mean output firing frequency evoked by stimulus trains with different g_syn_ values in ten cells (fitted equation has the form I_max_ (1 − exp(−af^n^)), where I_max_ = 83 bits/s, a = 0.1, n = 1.2, and f is frequency). (D) Energy use on pumping out of postsynaptic ion influx as a function of g_syn_ multiplier used in dynamic clamp. (E) Information divided by energy used on reversing the ion influx generating postsynaptic currents as a function of g_syn_ in ten cells (with, for each cell, the efficiency being normalized to the value at g_syn_ × 1; individual data for each cell are shown in Figure S3). The averaged data, shown in black, reveal a maximum very close to the physiological value of g_syn_. 100% corresponds to 15.6 ± 2.7 bits per 10^8^ ATP molecules used. Equation fitted to the mean data has the form 100.g_syn_/g_opt_ for g_syn_ < g_opt_ and 100.exp(−(g_syn_ − g_opt_)/K) for g_syn_ > g_opt_, where g_opt_ = 0.91 and K = 9.36. (F) Information divided by energy use on reversing the ion influx generating postsynaptic currents and postsynaptic action potentials also shows a maximum near the physiological value of g_syn_. Data are averaged over ten cells. 100% corresponds to 9.0 ± 1.2 bits per 10^8^ ATP molecules used. Fitted equation is as in (E) but with g_opt_ = 0.78 and K = 9.95. Data are represented as mean ± SEM. See also Figures S3–S5.

We calculated the energy use on the postsynaptic current for each value of the synaptic conductance—either evoked with presynaptic action potentials (Figures 4A–4C) or scaled and injected with dynamic clamp (Figures 4D and 4E)—by calculating the Na^+^ entry through the postsynaptic glutamate-gated conductance and then converting this to ATP use, knowing that one ATP molecule is consumed by the Na^+^ pump to extrude three Na^+^ ions [6] (see Experimental Procedures). Depolarization of the cell by the postsynaptic current, or by the action potential it evokes, reduces slightly the Na^+^ entry through postsynaptic channels compared to the situation in which the cell is voltage clamped at its resting potential (Figure 4F). As a result, the energy used on the postsynaptic current increases slightly less than linearly with the effective postsynaptic conductance (see Figure 5D below). Knowing both the information transmitted and the energy used, we could then examine the energetic efficiency of the synapse.

### Information Transmission and Energy Efficiency at Different Synaptic Conductances

Examining the output information rate as a function of the postsynaptic conductance (scaled up or down using dynamic clamp) showed that a transmitted information rate 4-fold higher than that observed with the biologically occurring synaptic conductance magnitude could occur if the postsynaptic conductance were increased (Figure 5A). Increasing the effective synaptic conductance value tended to increase the mean output firing rate (Figure 5B), although in about 30% of cells at large conductance values a depolarizing block (caused by sodium channel inactivation) set in and the firing rate declined again. The information transmitted increased roughly linearly with firing rate but then reached a plateau (Figure 5C) that was slightly less than the 94 bits/s of information present in the input train.

The data in Figure 5A demonstrate that the magnitude of the postsynaptic conductance is not set so as to maximize information flow through the synapse; indeed only a small fraction of the input information is successfully transmitted. Could this apparently sub-optimal arrangement be due to the large energetic cost of synaptic transmission?

We calculated the ratio of the information transmitted through the synapse (Figure 5A) to the energy used on postsynaptic currents (Figure 5D) when dynamic clamp was used to inject synaptic conductances of different magnitude. Data from individual cells are shown in Figure S3, with the average over ten cells shown in Figure 5E. Strikingly, the ratio of information transmitted to energy used showed a maximum, which was at the physiological conductance value for six of the ten cells and between 0.5 and three times the physiological value for a further three cells. Only one cell had a maximum substantially away from the physiological value (at nine times the physiological conductance, although even this cell also had a smaller local maximum at the physiological value). Thus, for most cells (Figure S3), and also for the average over all the cells (Figure 5E), either a decrease or an increase of the synaptic conductance from its physiological value leads to a remarkable decrease in energetic efficiency for the synapse. For example, increasing the synaptic conductance by a factor of 12, which maximizes information transmission through the synapse (Figure 5A), more than halved the number of bits of information transmitted per ATP used (Figure 5E). To estimate the position of the peak of the relationship (the optimum), we fitted a curve with the form 100.g_syn_/g_opt_ for g_syn_ < g_opt_, and 100.exp(−(g_syn_ − g_opt_)/K) for g_syn_ > g_opt_ to data from each cell and varied g_opt_ (and K) to minimize the sum of the squares of the residuals of the fit. The resulting mean value of g_opt_ was 0.90 ± 0.10 (which is not significantly different from 1; p = 0.33). Thus, the optimal value of postsynaptic conductance for maximizing the information transmitted per energy used on synaptic currents is not significantly different from the physiologically observed value.

If the postsynaptic current is sufficient to trigger an action potential, an additional energetic cost of restoring ion gradients after the action potential will be incurred. The action potential energy cost per second can be calculated as the product of the cost per action potential (which was 2.05 ± 0.11 × 10^7^ ATP molecules for LGN relay neurons; see Experimental Procedures) and the observed firing frequency of the cell. This cost was added onto the synaptic energy cost calculated above, and the information transmitted was calculated relative to the sum of the energy expended on EPSCs and action potentials, as a function of the synaptic conductance scaling factor (Figure 5F). Again, either a decrease or an increase of the synaptic conductance from its physiological value reduced the energetic efficiency of information transmission (Figure 5F). For each cell, we estimated the position of the peak of the relation in Figure 5F, using the same equation as above. The mean value of g_opt_ was 0.75 ± 0.13 (which is not significantly different from 1; p = 0.08). Thus, the optimal value of postsynaptic conductance for maximizing the information transmitted per energy used on synaptic currents *and* postsynaptic action potentials is again not significantly different from the physiological value.

### Modeling the Energetic Efficiency of Visual Synapses

To check that the membrane currents known to be present in LGN neurons were sufficient to generate the variation of energetic efficiency with synaptic conductance magnitude that is seen in Figure 5, we set up a Hodgkin-Huxley type of mathematical model of these cells, with current amplitudes set to those seen experimentally (see Supplemental Information). For this LGN relay neuron model (Figure S4), we found a dependence of information transfer, energy use, and energetic efficiency on synaptic conductance that was broadly similar to that measured experimentally (Figure 5), with a peak value of information transmitted per energy used at a synaptic conductance close to the normal physiological value.

## Discussion

We have examined how information flow through excitatory synapses is related to the size of the conductance activated by glutamate at the postsynaptic terminal and hence to the energy expended on postsynaptic ion influx. Strikingly, at the retinal ganglion cell to lateral geniculate nucleus synapse, an increase of postsynaptic conductance (implemented experimentally using dynamic clamp) can increase information flow through the synapse 4-fold (Figure 5A), demonstrating that the synapse properties are far from being optimized to maximize information transmission. Indeed, only about one in five presynaptic action potentials evokes a postsynaptic action potential. However, calculating the energy used on pumping out the Na^+^ ions that enter through the postsynaptic conductance (Figure 5E) and during postsynaptic action potentials (Figure 5F) shows that the evolutionarily selected value of the conductance maximizes the ratio of the number of bits of information transmitted through the synapse to the ATP used on ion pumping.

Similar computational sacrifices in favor of energetic efficiency have been observed elsewhere in the brain. The mean firing rate of CNS neurons (∼4 Hz) [6, 21] is much less than the rate that would maximize information coding capacity (half the maximal firing frequency or around 200 Hz for a refractory period of 2.5 ms) [7]. This has been explained [7] in terms of neurons maximizing the ratio of the amount of information they represent to the energy used on propagating the information as action potentials (which may itself be reduced by optimization of the properties of the active conductances generating action potentials) [22, 23, 24]. Similarly, the surprisingly low release probability of central synapses has been explained [4] in terms of presynaptic terminals operating to maximize the information transmitted per energy used. The results presented here extend this concept to postsynaptic terminals, the largest consumers of energy in the brain. Together with previous results recognizing that energy use is a significant constraint on neuronal function [6, 7, 21, 25, 26, 27], our data suggest maximization of information transmission per energy used is an important functional principle in the brain.

These conclusions were obtained for the retinal ganglion cell to lateral geniculate nucleus cell excitatory synapse studied in isolation in brain slices, with cortical input removed and inhibition blocked pharmacologically. This approach was taken in order to analyze the relationship between information transfer and energy use for a single synapse. In vivo, the presence of cortical input and local inhibition might significantly alter the overall transmission of information through the LGN. However, the fraction of input information that is transmitted that we find is similar to that measured in in vivo experiments (see Results) and when experiments were carried out without GABA receptors blocked the results obtained were similar (see Supplemental Information).

Whether energy optimization governs the postsynaptic properties of all excitatory synapses is still unknown. Minimization of postsynaptic energy consumption is, however, likely to be an important constraint on the operation of the weak parallel fiber to Purkinje cell synapse in the cerebellum because, after motor learning, approximately 85% of these synapses are turned off [28], greatly reducing the energy consumption of the cerebellar cortex [5]. At the other end of the spectrum are synapses like the calyx of Held or neuromuscular junction, where one synaptic input is sufficient to drive a highly reliable postsynaptic response. At such synapses, it may seem that faithful transmission must be favored at the expense of energy efficiency. However, research at the calyx of Held has shown that, over development, vesicle exocytosis becomes more efficient and release probability decreases, reducing postsynaptic receptor saturation and desensitization [29, 30], suggesting that such synapses do not use more resources than are necessary to transmit high fidelity information. We think it likely that close examination of a variety of synapses will reveal a widespread principle of energy-efficient information transmission in the brain.

Optimization of the energetic efficiency of synapses may confer additional coding benefits. The fact that the postsynaptic conductance at the retina-LGN synapse is not large enough to guarantee transmission of every retinal action potential not only maximizes the information transmitted per ATP used, as shown above, it also results in a more-efficient transmission of action potentials that occur close together in time [12] (Figure 1E), presumably because postsynaptic summation is needed to reach the threshold for production of a postsynaptic action potential. This occurs despite a decrease of EPSC size occurring (Figure S1) for the second of two action potentials that are close together in time (which can be viewed as a type of gain control) and leads to a change of code at the retina-LGN synapses, from a code where action potential correlations carry most information to a code where each action potential encodes information independently [31]. It is important to realize, however, that this modulation of EPSC size by paired pulse depression is automatically taken account of in our information analysis.

If the ratio of information transmitted to energy consumed at synapses has to be optimized for normal brain function, this raises the question of whether neurological or psychiatric disorders may arise when this ratio is perturbed. Insertion of too few postsynaptic receptors will lead to an excessive loss of information, whereas inserting too many postsynaptic receptors could increase the local energetic demand beyond that that can be met by the ATP supply from local mitochondria. To understand how the brain avoids these problems, it will be necessary to identify the mechanisms by which neurons assess how well they are optimizing information flow in relation to energy consumption. Intriguingly, energy reduction techniques are being introduced to nanoelectronics in which relatively unimportant connections in a semiconductor chip are removed in order to save energy, at the cost of some degradation in the accuracy of the computation performed [32, 33]. This probabilistic pruning of the circuitry has an effect similar to the sacrifice of information transfer made by neurons that adopt a low postsynaptic conductance in order to save energy.

## Experimental Procedures

### Visual Pathway Slice Preparation

P28 Sprague Dawley rats were killed by overdose of isoflurane anesthetic, in accordance with the guidelines of the UK Animals (Scientific Procedures) Act 1986 and subsequent amendments. The brain was rapidly removed and immersed in ice-cold, slicing solution containing (in mM) 87 NaCl, 25 NaHCO_3_, 7 MgCl_2_, 2.5 KCl, 1.25 NaH_2_PO_4_, 0.5 CaCl_2_, 25 glucose, 75 sucrose, and 1 kynurenic acid, saturated with 95% O_2_/5% CO_2_ (modified from [34]).

Parasagittal brain slices containing the dorsal lateral geniculate nucleus (dLGN) were obtained as described [8]. Briefly, each hemisphere was isolated using a cut either side of the midline at 3°–5° to the sagittal plane, angled outward by 10°–25° in the mediolateral plane. The medial side of each brain half was glued to the cutting stage of a vibratome (Leica VT1200S) and submerged in ice-cold continuously oxygenated slicing solution, and 225-μm slices were made. In general, only a single slice from each hemisphere contained the optic tract and its fibers radiating to the dLGN. Before use, the cortex of each slice was removed using a scalpel to prevent disynaptic excitation via the thalamocortical feedback loop.

Slices were placed in a storage chamber containing continuously oxygenated slicing solution at 35°C, which was allowed to come to room temperature naturally. During the experiment, slices were continuously perfused with artificial cerebrospinal fluid (aCSF) containing (in mM) 124 NaCl, 26 NaHCO_3_, 10 glucose, 2.5 KCl, 2 CaCl_2_, 1 NaH_2_PO_4_, 1 MgCl_2_, and 0.005 gabazine (to block disynaptic inhibition during stimulation). The aCSF was heated to 35°C and constantly bubbled with 95% O_2_/5% CO_2_.

### Electrophysiology

Whole-cell recordings from LGN relay neurons were obtained using 2- to 3-MΩ borosilicate glass electrodes filled with internal solution containing (in mM) 130 K-gluconate, 10 EGTA, 10 HEPES, 4 NaCl, 4 MgATP, 1 CaCl_2_, 0.5 Na_2_GTP, and 0.4 K_2_-Lucifer yellow. Relay neurons were identified by their large cell bodies (15–25 μm) and the presence of a hyperpolarization-activated inward current [35]. Throughout the experiment, relay neurons were held at −55 mV (by injection of a small amount of current, as resting potentials were typically around −70 mV), in order to restrict them to firing in tonic mode, where one input spike generally produces one output spike, as opposed to burst mode at <−70 mV, where one input spike generally leads to a burst of output spikes [36, 37].

Online corrections were made for the junction potential of −14 mV for the gluconate-based internal solution used (e.g., neurons were held at an apparent potential of −41 mV to achieve a true potential of −55 mV for LGN cells). Recordings were made with an Axopatch 200B amplifier, filtered at 5 kHz, and sampled at 20 kHz. Data were acquired using custom-made MATLAB software, kindly provided by Ho Ko and Tom Mrsic-Flogel, UCL.

The first part of each experiment was performed in voltage clamp. Upon seal formation, pipette capacitance was compensated. Once in whole-cell mode, the series resistance was compensated by up to 70% (after which the mean series resistance was 6.7 ± 0.6 MΩ). The second part of the experiment was performed in current clamp, using the I-CLAMP FAST mode (which was stable with the 2- to 3-MΩ pipettes used). In current clamp mode, series resistance compensation was set to 100%.

Retinal ganglion cell (RGC) axons in the optic tract were stimulated extracellularly with a borosilicate glass electrode (gently broken to achieve a tip diameter of ∼10–15 μm) containing aCSF. In voltage clamp, stimulation was adjusted to achieve the smallest reliable EPSC (defined as an EPSC that, when it occurred, did not vary in size in response to a pulse delivered every 3 s). The EPSC size usually increased in one clear step, and the stimulus intensity could therefore be set to activate a single presynaptic RGC axon [10, 11] (Figure S1A). This intensity was then maintained throughout the experiment. The average EPSC size (949 ± 141 pA) was similar to that found previously at this age [10, 11] (Figure S1B). All recordings used showed the strong paired pulse depression characteristic of this synapse [10, 11, 38] (PPR = 0.39 ± 0.05; Figures S1C and S1D).

### Stimulation Pattern

RGC axons were stimulated (Figures 1A and S2A) with five 5-s spike trains (average frequency ∼19 Hz), recorded [9] from ON-RGCs in isolated mouse retinae in response to five natural movies and kindly provided by Sheila Nirenberg, Cornell. We cannot be sure that the type of ganglion cell axon stimulated is exactly the same as that recorded in the Nirenberg experiments. Nevertheless, there are no publications (to our knowledge) suggesting that the output synapses of different classes of ganglion cell differ in their mechanisms. Determining the energy efficiencies of the synapses to LGN cells from different classes of ganglion cell will be an interesting area to study in future.

After an initial run of train 3 to habituate the synapse (EPSCs tended to be substantially larger directly after a period of no stimulation; Figure S1C), from which the data were discarded, the five trains were played in sequence (1-2-3-4-5), five times, resulting in a 125-s stimulation train from which data were collected. This procedure was followed once in voltage clamp, once in current clamp, and several times in dynamic clamp with various conductance gains (see below).

### Dynamic Clamp

The 125-s postsynaptic current recording obtained from LGN relay neurons in voltage clamp was used to calculate a 125-s conductance train. First, the stimulation artifacts were removed by setting the current value for the duration of the artifact to the current value immediately preceding the artifact (Figures 2B, inset, and 2D, inset). The resulting current trace (I_syn_) was converted to a conductance trace (g_syn_) via

$$g_{\mathrm{syn}}\left(t\right)=I_{\mathrm{syn}}\left(t\right)/\left(V_{m}-V_{\mathrm{rev}}\right),$$

where V_m_ is the membrane potential of the cell (the holding potential; −55 mV) and V_rev_ is the reversal potential of the synapse (0 mV; the reversal potential for glutamatergic ionotropic receptors). g_syn_ was then scaled up or down by a factor of 0.1, 0.3, 0.5, 0.75, 1, 1.5, 3, 6, 9, or 12. The new 125-s conductance trace (g_syn_) was then applied directly to the postsynaptic cell using dynamic clamp [20] (SM-1; Cambridge Conductance), which injects a time-varying current I_inj_(t), at time t, calculated from g_syn_(t) and the instantaneous value of the cell membrane potential

$$I_{\mathrm{inj}}\left(t\right)=g_{\mathrm{syn}}\left(t\right)\times \left(V_{m}\left(t\right)-V_{\mathrm{rev}}\right).$$

Because of the liquid junction potential, the V_m_ received by the SM-1 was 14 mV more positive than the real membrane potential. We therefore set V_rev_ on the SM-1 to 14 mV (rather than 0 mV) to account for this in the online calculation of I_inj_. In this calculation, all of the synaptic current was assumed to scale linearly with membrane potential (i.e., non-linearities related to the magnesium block of NMDA receptors were not mimicked here, but detailed simulations showed that this had no qualitative effect on the relationship between synaptic conductance and the ratio of information transmitted to energy used; Figure S4).

The voltage response of the postsynaptic cell was simultaneously recorded. When the conductance was scaled by 1 and applied by dynamic clamp, the postsynaptic firing pattern was similar to that recorded when electrical stimulation was applied presynaptically (compare Figures 2C and 2E). Thus, although in dynamic clamp the conductance increases were applied at the soma rather than in the dendrites, this did not appear to affect the cell’s decision to spike. This was probably because (1) the conductance injected at the soma was originally recorded at the soma was originally recorded at the soma, and was thus already “filtered” by the dendrites, and may mimic the current injected into the soma from the dendrites during synaptic simulation; and (2) relay neurons in the LGN are highly electrically compact [39], which, along with the close proximity of the retinogeniculate synapse to the cell body [40] (<100 μm), implies that the retinal input seen by the soma is only mildly attenuated compared to that seen by the dendrites.

To prevent possible damage to the cell with large injected conductances, the traces scaled by 6, 9, or 12 were always injected last. The order of injection of the smaller conductances was randomized.

### Data Analysis

Data were analyzed using custom scripts written in MATLAB (The Mathworks). Postsynaptic current traces were used to calculate ATP consumption at the synapse as described below. Postsynaptic voltage traces were converted to binarized sequences of 1 s (representing action potentials) and 0 s (their absence) by identifying events whose amplitude exceeded a threshold defining action potential occurrences (set individually for each cell; between −15 mV and −30 mV). This output sequence could then be compared with the binary input spike train to look at simple transmission characteristics (Figure 1) or used to calculate the amount of information that would be propagated to visual cortex by the postsynaptic cell (Figures 3 and 5).

### Synapse Transmission Characteristics

To assess how the probability of an output spike depends on presynaptic inter-stimulus interval (ISI) at the RGC-LGN synapse, output spikes were searched for in an 18-ms time window following the second spike of an ISI pair (18 ms was chosen because it encompasses the majority of the action potentials evoked by an input at all dynamic clamp gains; Figures S5A–S5D). If an output spike was present, the preceding ISI was counted as “relayed”; if not, the preceding ISI was counted as “non-relayed.” The probability distribution for each category was calculated based on the total frequency counts for all 18 LGN relay neurons studied (Figure 1E). Note that, if a single presynaptic spike can sometimes evoke a postsynaptic action potential, this procedure has the potential to artifactually indicate action potential production by large ISIs when in fact it was only the second action potential of a presynaptic pair that produced the postsynaptic action potential; consequently, this procedure overestimates the frequency of action potential production by large ISIs.

To assess the occurrence of output responses produced by an input spike, the 18 ms following each input spike was searched for either a 1 in the binarized output trace (indicating a postsynaptic action potential) or an EPSP in the original voltage trace (with a minimum threshold of 1 mV). If neither of these were found, no output response was considered to have occurred. If two inputs arrived within 18 ms of each other, the search window following the first input was ended at the time of the second input. The probability of each outcome was calculated from its relative occurrence across all 18 cells in Figures 1F and 1G.

To assess the probability of an input spike given an output spike, the 18 ms preceding each output spike was searched for a 1 in the binarized input trace (indicating an input spike). If this was not found, the postsynaptic spike was considered to have occurred spontaneously. If two output spikes occurred within 18 ms of each other, the search window preceding the second output spike was ended at the time of the first output spike. The probability of each outcome was calculated from its relative occurrence across all 18 relay cells (Figures 1F and 1H).

The value chosen for the time window after a presynaptic spike or before a postsynaptic spike is not critical. Its main effect is on the pie charts in Figures 1G and 1H. Reducing the window to 9 ms or increasing it to 30 ms alters the percentage of occasions on which a presynaptic AP generates a postsynaptic AP from 19% (Figure 1G) to 16% or 20%, respectively (as a longer time window results in more postsynaptic APs being found). More significantly, the same alterations of time window alter the percentage of output APs that are not associated with an input AP from 7% (Figure 1H) to 20% or 3%, respectively (again because a longer time window results in more presynaptic APs being found).

### Calculating Synaptic Energy Use

For voltage-clamp conditions, the ATP used to reverse the postsynaptic ion flux (which is the largest synaptic energy cost) [4] was calculated from the postsynaptic current trace recorded in response to presynaptic stimulation. The current trace was integrated to obtain the total charge entry over the 125-s recording (Figure 4C). Dividing this by the charge on a Na^+^ ion gives an estimate of the Na^+^ influx. However, because K^+^ efflux is occurring simultaneously (through the non-specific cation pores of AMPA and NMDA receptors), the actual Na^+^ entry is 1.42 times larger than this (see next paragraph). The total Na^+^ influx must then be actively pumped out by the Na^+^/K^+^-ATPase, which uses one ATP molecule per three Na^+^ ions. This ATP cost was divided by the length of the recording (125 s) to get a rate of energy consumption (in ATP molecules/s) for each cell.

For voltage recording in current clamp mode using dynamic clamp, with the synaptic conductance scaled up or down, energy consumption was calculated differently. First, the synaptic Na^+^ conductance (g_Na_) was calculated from the total synaptic conductance (g_syn_) by assuming that the contributions to the total AMPA receptor current carried by Na^+^ and K^+^ (Ca^2+^ was neglected) vary ohmically with voltage displacement from the reversal potentials V_Na_ (+90 mV) and V_K_ (−105 mV), so that (for a synaptic current reversal potential of V_rev_ = 0 mV)

$$g_{\mathrm{Na}}=g_{\mathrm{syn}}/\left(\mathrm{1-}\left(V_{\mathrm{Na}}/V_{K}\right)\right).$$

For the experimentally imposed reversal potentials stated above, g_Na_ = (7/13).g_syn_. The Na^+^ current (I_Na_) was then calculated directly from the Na^+^ conductance, the Na^+^ reversal potential, and the membrane potential of the cell as

$$I_{\mathrm{Na}}\left(t\right)=g_{\mathrm{Na}}\left(t\right)\times \left(V_{m}\left(t\right)-V_{\mathrm{Na}}\right).$$

The integral of I_Na_(t) was then used to calculate the total postsynaptic Na^+^ entry over the 125-s recording (Figure 4E), which was converted to a rate of energy consumption as above. For voltage-clamp experiments at our holding potential of −55 mV, the Na^+^ entry calculated from the equation above can be shown (using the relationship between g_Na_ and g_syn_ given above) to be 1.42-fold larger than the charge entry measured from the synaptic current as g_syn_(t) × (V_m_(t) − V_rev_).

### Calculating Action Potential Energy Use

Action potentials in rodent thalamocortical relay neurons have been found to be highly energy efficient, costing 1.35 × 10^11^ ATP molecules/AP/cm^2^ of membrane [24]. From the recorded membrane capacitance for each LGN cell (mean = 152 ± 8 pF) and the standard biological membrane capacitance (1 μF/cm^2^), we could calculate the surface area, and thus the action potential cost, for each cell (mean = 2.05 ± 0.11 × 10^7^ ATP molecules/AP/cell). This value—calculated for each LGN cell—was multiplied by the firing frequency of the cell in each stimulation condition to obtain the energy used on action potentials per second.

### Calculating Information

To calculate the information transmitted across a synapse, we employed information theory [41] to estimate the mutual information between the input and output spike trains. The calculations are described in detail in the Supplemental Information. The so-called direct method [17, 19] requires an input train made up of unique and repeating sections, responses to which are used to calculate the total entropy and the noise entropy in the output signal, respectively. Mutual information is the total entropy minus the noise entropy. A major advantage of this method is that it does not require any assumptions about correlations between spikes or the temporal relationship between input and output spikes. An alternative method gave similar results (see Supplemental Experimental Procedures and Figure S5D).

### Analysis of Energetic Efficiency

For each condition (real stimulation and all dynamic clamp gains), the information rate was divided by the rate of energy consumption on reversing the ion flux generating EPSCs (Figure 5E), or on reversing the ion flux generating EPSCs and postsynaptic action potentials (Figure 5F), to get a measure of efficiency in bits/(ATP consumed).

### Statistics

Data are presented as mean ± SEM. Differences between means were assessed with Student’s t tests and corrected for multiple comparisons using a modified Holm-Bonferroni method; differences are taken as significant when p < 0.05.

## Author Contributions

J.J.H., R.J., and D.A. conceived experiments. J.J.H., R.J., and E.E. performed experiments. R.J., J.J.H., and E.E. performed analyses. J.J.H., R.J., E.E., and D.A. wrote and reviewed the manuscript. D.A. supervised and acquired funding.

## Supplemental Information

Document S1. Figures S1–S5 and Supplemental Experimental Procedures

Document S2. Article plus Supplemental Information
