# Supplementary material for: Energy-Efficient Information Transfer by Visual Pathway Synapses
Source: Curr Biol. 2015 Dec 21;25(24):3151–60. doi: 10.1016/j.cub.2015.10.063 (PMC4691239; doi:10.1016/j.cub.2015.10.063)
Supplement: Document S1. Figures S1–S5 and Supplemental Experimental Procedures [file mmc1.pdf]

Current Biology
Supplemental Information

Energy-Efficient Information Transfer by Visual Pathway Synapses

Julia J. Harris, Renaud Jolivet, Elisabeth Engl, and David Attwell

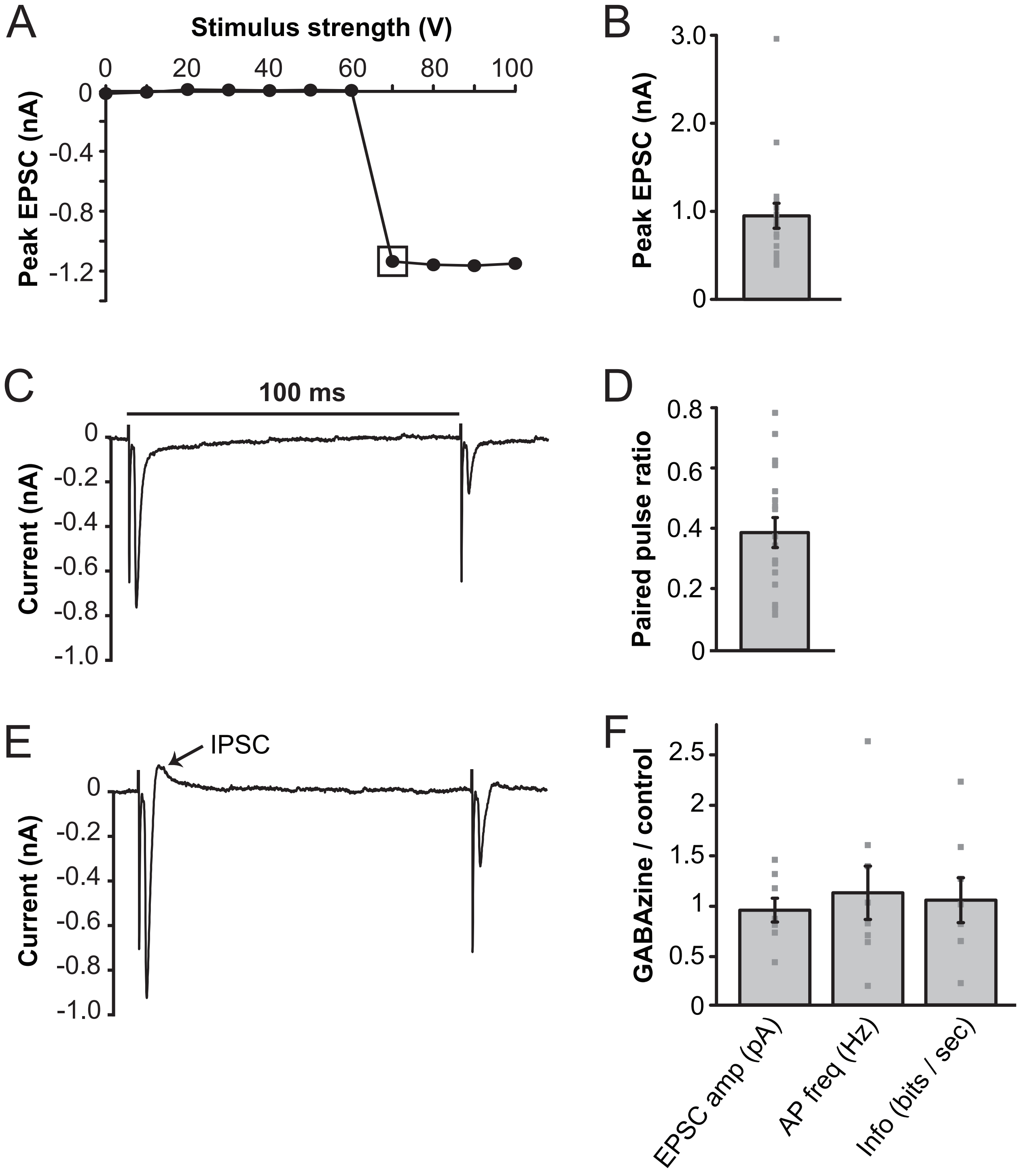
A
Stimulus strength (V)
0
20
40
60
80
100
Peak EPSC (nA)
0
-0.4
-0.8
-1.2
B
3.0
2.0
1.0
0
Peak EPSC (nA)
C
100 ms
0
-0.2
-0.4
-0.6
-0.8
-1.0
Current (nA)
D
0.8
0.6
0.4
0.2
0
Paired pulse ratio
E
IPSC
0
-0.2
-0.4
-0.6
-0.8
-1.0
Current (nA)
F
2.5
2
1.5
1
0.5
0
GABAzine / control
EPSC amp (pA)
AP freq (Hz)
Info (bits / sec)

Figure S1

Figure S1 (related to Figure 1). Characterization of the optic tract input to dLGN cells. (A) Stimulus-response curve shows a single increase indicating stimulation of one axon. (B) Mean amplitude (bar and s.e.m.) of first excitatory postsynaptic current (EPSC) in 18 cells (points). (C) Paired pulse depression of EPSC in a specimen cell evoked by stimuli 100 msec apart. (D) Mean (bar and s.e.m.) paired pulse ratio (2nd / 1st EPSC) for 100 msec separation in 18 cells (points). (E) In the absence of GABAzine, some cells display disynaptic inhibition (seen as an IPSC after the EPSC), which tends to show postsynaptic depression (mean paired pulse ratio of IPSC = 0.6±0.2). (F) For the cells with disynaptic inhibition, GABAzine does not significantly alter the EPSC size ($p = 0.62$), the action potential frequency ($p = 0.69$) or the information rate ($p = 0.87$) of presynaptically stimulated LGN neurons (8 cells) (bars with s.e.m. are mean data; individual cells shown as points).

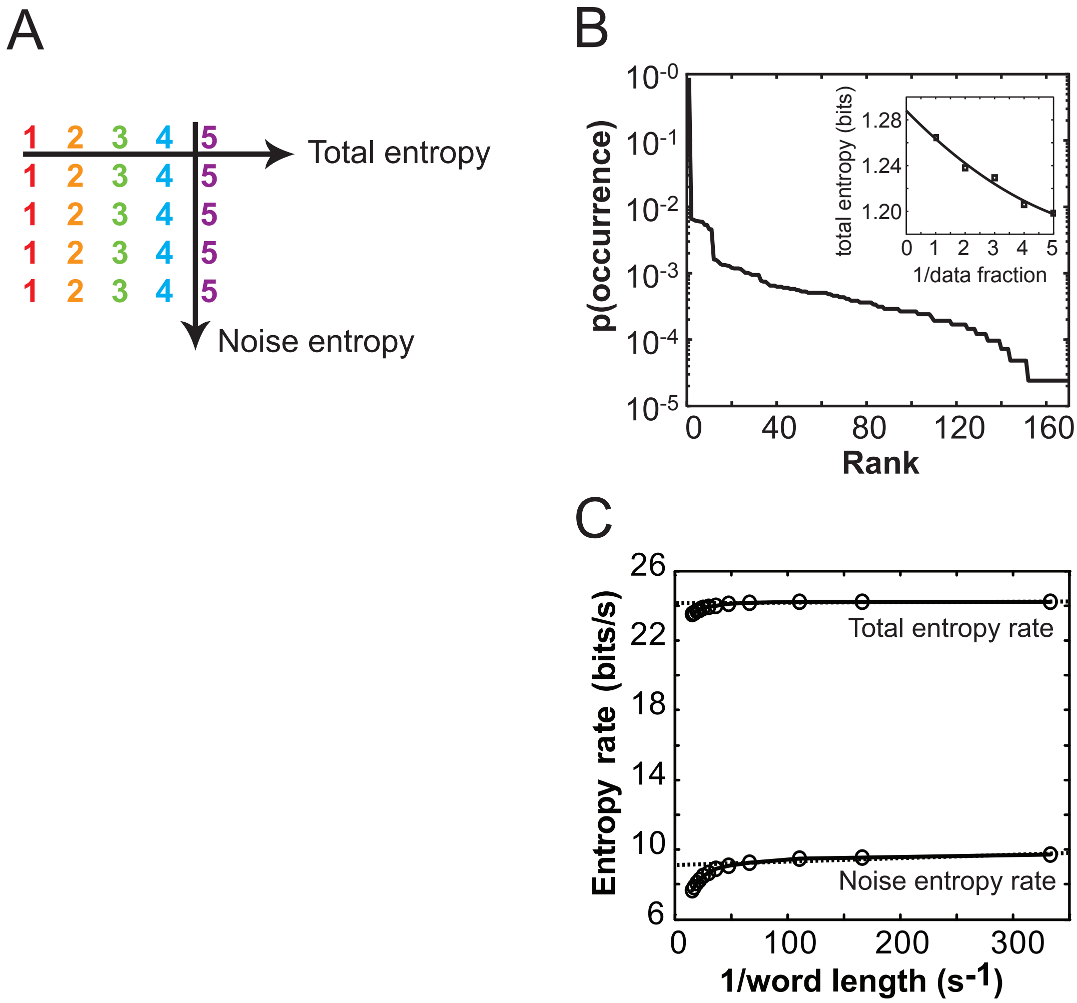
A
1 2 3 4 5
Total entropy
Noise entropy
B
p(occurrence)
Rank
total entropy (bits)
1/data fraction
C
Entropy rate (bits/s)
Total entropy rate
Noise entropy rate
1/word length (s-1)

Figure S2

Figure S2 (related to Figure 3). Calculating mutual information at the RGC-LGN synapse using the direct method. (A) Trains 1-5 were applied 5 times (in the order left to right, and then down the successive rows). Analysis of entropy across all 5 trains gives a measure of total response variability. Analysis of entropy across repeated application of the same train gives the noise entropy. (B) Probability of occurrence of each of the 10-letter words produced by application of all 5 trains for a specimen cell, ranked by probability value. Inset shows the dependence of the entropy, computed from this probability distribution according to the Shannon formula [3] (see Supplemental Experimental Procedures), on the fraction of data included in the analysis. Also plotted is a least squares fit of a quadratic function (see Supplemental Experimental Procedures), where the intercept is our extrapolation to the true value of the entropy with infinite data [1]. (C) Total and noise entropy for a specimen cell as a function of 1/word length (a word length of ten 3 msec bins corresponds to 1/word length = 33 sec^{-1}). Dashed lines indicate linear extrapolations to infinite word length [1].

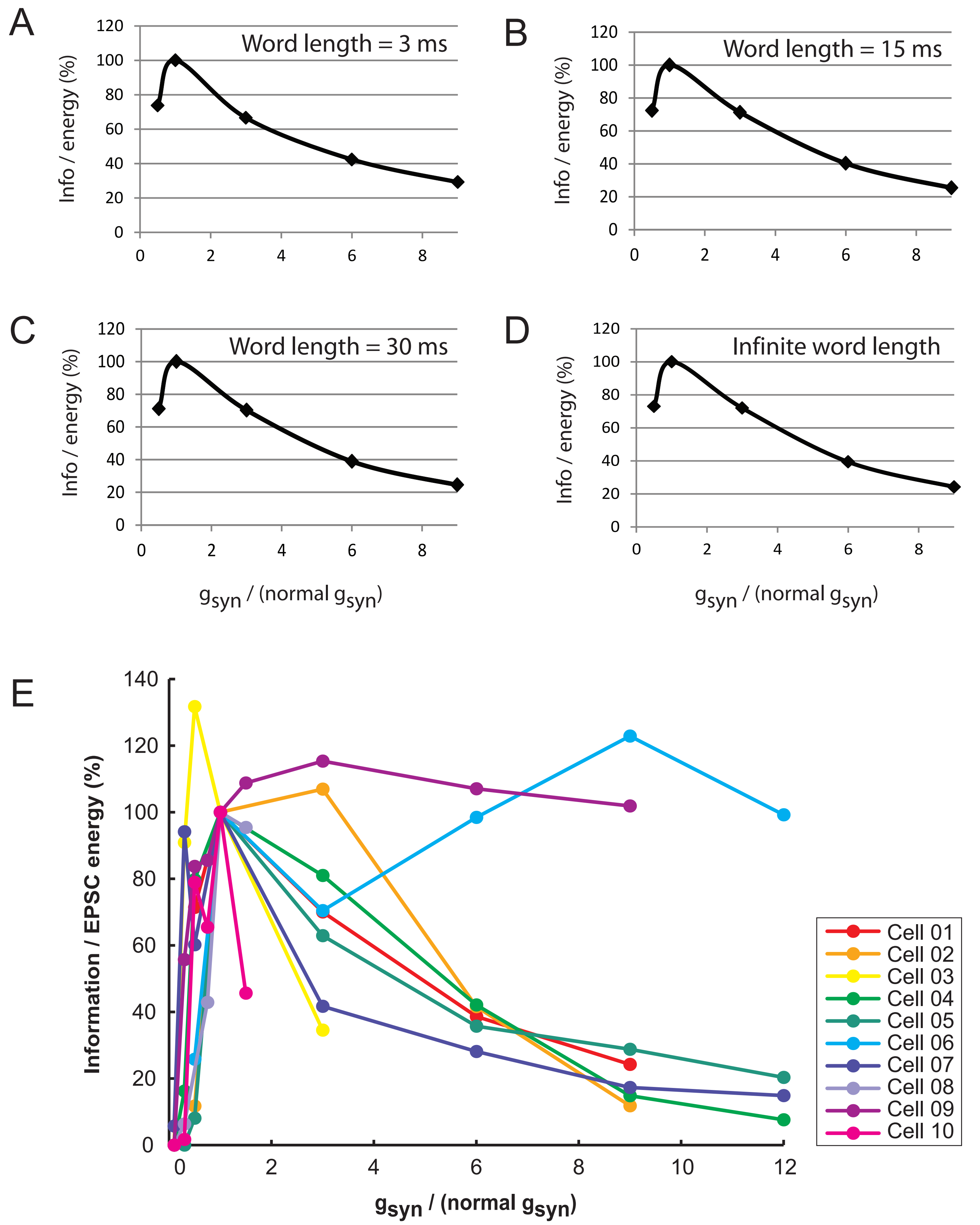
A
Word length = 3 ms
B
Word length = 15 ms
C
Word length = 30 ms
D
Infinite word length
Info / energy (%)
gsyn / (normal gsyn)
E
Information / EPSC energy (%)
gsyn / (normal gsyn)
Cell 01
Cell 02
Cell 03
Cell 04
Cell 05
Cell 06
Cell 07
Cell 08
Cell 09
Cell 10

Figure S3

Figure S3 (related to Figure 5). Information divided by energy used on reversing the ion influx generating postsynaptic currents as a function of g_{syn}. (A-D) Efficiency (bits transmitted / ATP used on EPSCs) plotted against synaptic conductance (altered using dynamic clamp to be 0.5, 1, 3, 6 and 9 times the biologically-occurring conductance) for an example cell. The relationship does not change when the information is calculated using words of length (A) 3 msec; (B) 15 msec; (C), 30 msec (used for all cells in Figure 5); or (D) linear extrapolation to infinite word length (as in Figure S2C). Bin size was always 3 msec. This analysis was performed on three cells, with similar results. (E) Information divided by energy used on reversing the ion influx generating postsynaptic currents in 10 individual cells plotted in different colours against g_{syn}. For each cell, the efficiency is normalised to the value at g_{syn}x1. Six out of ten cells show a maximum efficiency at the biological conductance value (g_{syn}x1), three cells show a maximum at a higher conductance magnitude (two at g_{syn}x3 and one at g_{syn}x9), and one cell shows a maximum at a lower conductance magnitude (at g_{syn}x0.5). The averaged data are shown in Figure 5E. 100% corresponds to 15.6±2.7 bits per 10^8 ATP molecules used.

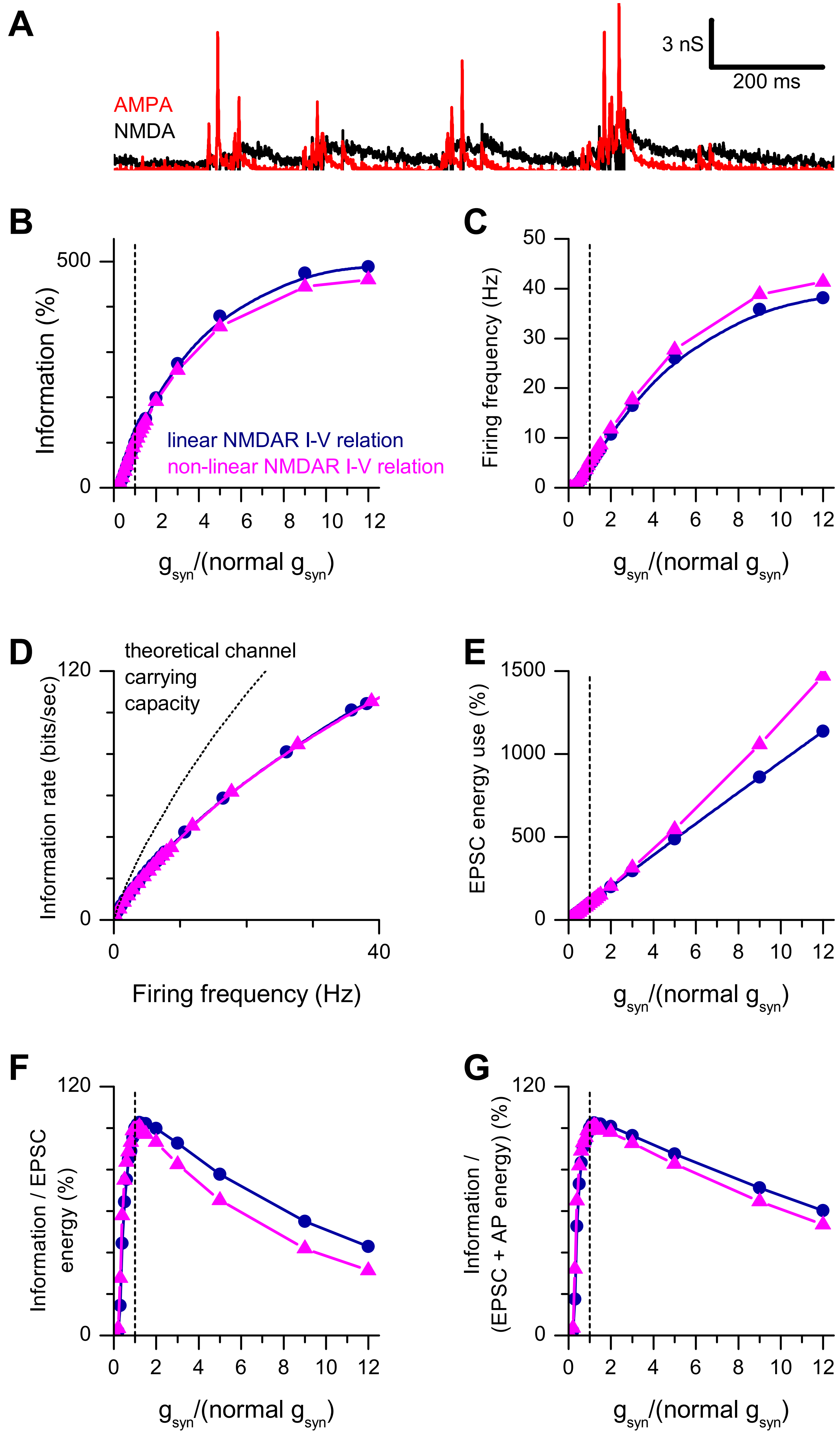
A
AMPA
NMDA
3 nS
200 ms
B
Information (%)
500
0
linear NMDAR I-V relation
non-linear NMDAR I-V relation
0 2 4 6 8 10 12
g_syn/(normal g_syn)
C
Firing frequency (Hz)
50 40 30 20 10 0
0 2 4 6 8 10 12
g_syn/(normal g_syn)
D
theoretical channel
carrying
capacity
Information rate (bits/sec)
120
0
0 40
Firing frequency (Hz)
E
EPSC energy use (%)
1500 1000 500 0
0 2 4 6 8 10 12
g_syn/(normal g_syn)
F
Information / EPSC
energy (%)
120
0
0 2 4 6 8 10 12
g_syn/(normal g_syn)
G
Information /
(EPSC + AP energy) (%)
120
0
0 2 4 6 8 10 12
g_syn/(normal g_syn)

Figure S4

Figure S4 (related to Figure 5 and Supplemental Experimental Procedures). Postsynaptic conductance magnitude maximising information transferred per energy used in a model of thalamic relay cells. (A) Specimen segments of the AMPA and NMDA conductance time course derived from experiments (see Supplemental Experimental Procedures) and used in the simulations. (B) Dependence of the output information on synaptic conductance (g_{syn}) magnitude, when the thalamic relay cell model (see Supplemental Experimental Procedures) was stimulated with the AMPA and NMDA conductance recorded experimentally in response to optic tract stimulation. The graph shows the effect of assuming a linear NMDAR I-V relation (blue) or a non-linear NMDAR I-V relation (violet; see Supplemental Experimental procedures). Information is normalized to the value with g_{syn} x1 for which the mean information rate was 21.4 bits/sec (linearized NMDA conductance) or 23.7 bits/sec (non-linear NMDA conductance). For comparison, the experimental information rate for recorded cells with presynaptic stimulation was 18.3±4.5 bits/sec. Colours are the same in B to F. (C) Relationship between firing frequency and g_{syn} in the simulations. (D) Dependence of output information on mean output firing frequency evoked by stimulus trains with different g_{syn} values. Linearizing the NMDAR I-V relation had no significant effect on this relation. The theoretical maximum channel capacity, given by:

$$-[f.\Delta t.\log_2(f.\Delta t) + (1-f.\Delta t).\log_2(1-f.\Delta t)]/\Delta t,$$

where $f.\Delta t$ is the probability of an action potential occurring in the temporal bin width Δt (3 msec) at a firing frequency f, is also shown. (E) Energy use on pumping out of postsynaptic ion influx as a function of g_{syn} multiplier. (F) Output information divided by energy use on reversing the ion influx generating postsynaptic currents in simulations shows a maximum close to the physiological value of g_{syn} (102.8% at g_{syn}=1.2 with the NMDAR I-V relation linearized and 100.8% at g_{syn}=1.2 with a non-linear NMDAR I-V relation). 100% (at g_{syn} = 1) corresponds to 31.9 bits per 10^8 ATP molecules used with the NMDAR I-V relation linearized and to 32.3 bits per 10^8 ATP molecules used with the non-linear NMDAR I-V relation, slightly larger than the experimental value of 15.6±2.7 bits per 10^8 ATP molecules used. (G) Output information divided by energy use on reversing the ion influx generating postsynaptic currents and postsynaptic action potentials also shows a maximum close to the physiological value of g_{syn} (102.5% at g_{syn}=1.2 with the NMDAR I-V relation linearized and 101.1% at g_{syn}=1.2 with a non-linear NMDAR I-V relation). 100% (at g_{syn} = 1) corresponds to 8.0 bits per 10^8 ATP molecules used with the NMDAR I-V relation linearized and to 8.1 bits per 10^8 ATP molecules used with the non-linear NMDAR I-V relation, slightly below the experimental value of 9.0±1.2 bits per 10^8 ATP molecules used.

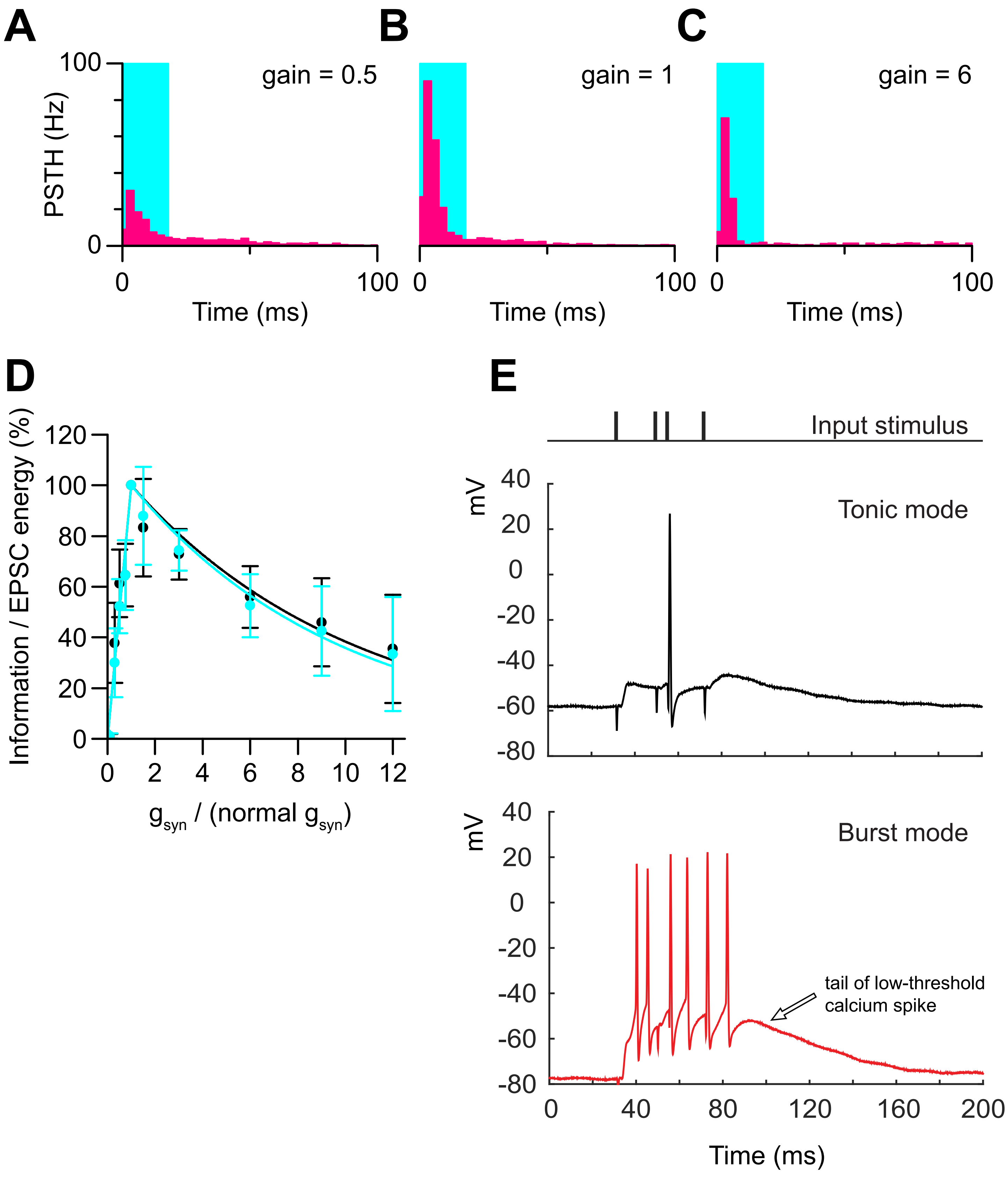
A
B
C
gain = 0.5
gain = 1
gain = 6
PSTH (Hz)
100
0
Time (ms)
D
E
Information / EPSC energy (%)
120
100
80
60
40
20
0
2
4
6
8
10
12
g syn / (normal g syn)
Input stimulus
Tonic mode
Burst mode
mV
40
20
-20
-40
-60
-80
tail of low-threshold calcium spike
160
200
Time (ms)

Figure S5

Figure S5 (related to Figure 5 and Supplemental Experimental Procedures). Sampling window for assessing action potential transmission, and comparison of energy efficiency as a function of g_{syn} when information is estimated with two different methods. (A-C) Peristimulus time histograms for one example cell at three different scaling values for g_{syn}/(normal g_{syn}) (0.5, 1 and 6) illustrating that an 18 ms search window (cyan rectangle) contains most of the PSTH at gains and mutual information rates covering almost the entire range of the LGN data. (D) Comparison of energetic efficiency at the retinothalamic synapse obtained using mutual information estimates obtained with the direct method (black; same data as in Figure 5E) and using the alternate calculation described in the Supplemental Experimental Procedures with a search window of 18 msec (cyan). A similar peak in the relationship at g_{syn}/(normal g_{syn}) = 1 was seen even using search windows as small as 9 ms or as large as 30 ms. Data are represented as mean ±s.e.m. (E) Spiking response to the same input stimulus when an example cell was held in tonic mode (at approximately -55 mV) and burst mode (at approximately -75 mV). In burst mode, a small depolarization can trigger a low-threshold calcium spike, upon which multiple action potentials “ride”. This often means that one input spike triggers multiple output spikes, raising the question of whether one takes a single spike or a burst of spikes as the “output” in this mode of firing.

SUPPLEMENTAL EXPERIMENTAL PROCEDURES

Information analysis: direct method applied to retinogeniculate data

To calculate the information transmitted across the retinogeniculate synapse, we assessed the possible repertoire of signal encoding achievable by the optic tract to LGN neuron synapse by applying five separate 5 sec input trains evoked by different natural scenes (Figure 3A). For each recording, we applied these five trains in sequence (1-2-3-4-5), five times (Figure S2A), thus generating a 125 sec output spike train for analysis. We assessed the variability of the output response to the input information (to obtain the "total entropy", calculated below, Figure S2B), and also the variability in output response to a single input train applied on five separate occasions (to give the "noise entropy", calculated below).

To calculate the stimulus-related information in the output spike train, we used the direct method [S1]. Binarised output spike trains were binned with 3 msec precision (approximately the action potential refractory period). The binned trains were examined in segments of a fixed length, called "words" (a particular sequence of 1s and 0s), each word representing a possible neural response. For example, for a word length of five (3 msec) bins, an example word would be 10010. Words were allowed to overlap, so that each time bin was the start of a new word [S1 and S2]. The action potential string 100100001… would thus yield the 5 letter words 10010, 00100, 01000, 10000, 00001, etc. This maximised the number of words available for analysis in the 125 sec output spike train, the effects of correlations in which are removed by extrapolation to infinite word length [S1 and S2] as in Figure S2C. Words that are too long can cause a sampling bias (due to insufficient data being acquired) in which case the entropy (calculated below) is underestimated [S1 and S2]. Choice of word length for the analysis to avoid this problem is discussed below.

A probability distribution of word occurrences was built (Figure S2B), and used to calculate, for two distinct situations (described below), the Shannon entropy [S3], H (in bits), using the general formula:

$$H = -\Sigma\, p(\text{word}) \times \log_2 [p(\text{word})]$$

where p is the probability of a particular word occurring.

First, a probability distribution was built for the occurrence of all possible words of a given length, by counting the frequency of occurrence of each word in the first 25 sec of stimulation (i.e. across the first run of all five trains, represented by the horizontal arrow in Figure S2A). This probability distribution (Figure S2B) was used to calculate the total entropy (H_{total}) of the first 25 sec of the spike train (using the formula above). H_{total} was calculated in this way for each successive run of the five different input trains (i.e. for each row in Figure S2A), and then averaged to get a final estimate of H_{total} for the whole 125 sec recording. H_{total} reflects the maximum possible variability of the postsynaptic response for the input trains used, and a higher value reflects a higher information capacity in the spike train.

Second, to assess variability in the response to a repeated input train [S1], a probability distribution was built for the occurrence of all possible words (of a given length) that were evoked at a fixed time by a single input train when it was repeated five times. To do this, for a given word length, and looking at the responses over the 5 repeated identical trains, we counted the frequency of occurrence of words starting at a set time, *t*, after the onset of each repeated train. H_{noise} was calculated in the window from *t* to *t*+(word length) in the repeated train (using the formula above), and then averaged across all time points. H_{noise} was calculated in this way for each different repeated train (i.e. for each column in Figure S2A) and then averaged to get a final estimate of H_{noise} for the whole recording. H_{noise} reflects the trial-to-trial variability of the response, and a lower value reflects low noise levels in the system (i.e. little variability in the response to repeats of the same input).

Subtracting H_{noise} from H_{total} gives an estimate of the mutual information (I, in bits) between the output and input spike trains [S1], in other words, how informative the output spike train is about the input spike train:

$$I = H_{total} - H_{noise}$$

Because extrapolating to infinite word length [S1] as in Figure S2C was very computationally time-consuming, we defined the maximum word length that could be used before sampling problems arose by varying the word length between 3 and 63 ms (Figure S2C). We found that total entropy estimates based on 30 msec words differed by only 1.2±0.4% (averaged across dynamic clamp conditions for 3 cells) from the entropy estimate based on a linear extrapolation to infinite word length [S1 and S2], while the resulting value of information rate differed by only 0.3±0.6% from the value obtained by extrapolation. Similarly, the relationship between efficiency and g_{syn} was not affected by the use of either extrapolated entropy estimates or entropy estimates based on 30 msec words (or, indeed, words shorter than 30 msec; 3 cells, Figures S3A-S3D). 30 msec words were therefore used for the entropy calculations that follow. Throughout the paper, entropy and information estimates derived from this method are divided by the word length (30 msec) to get entropy and information rates in bits/sec.

Data adequacy for entropy calculations

Entropy estimates based on insufficient data could yield misleading results. However, there are significant experimental constraints on the length of whole-cell recordings (applying synaptic stimulation in voltage and current clamp modes, and then applying up to eight different conductance amplitudes in dynamic clamp takes around one hour and requires exceptional stability of the recorded cell). We therefore used each stimulus train (1-5, Figure 3A and Figure S2A) as both a “unique” input (to calculate total entropy) and a “repeat” input (to calculate noise entropy), thus maximising the utility of the data we collect. We checked whether this amount of data was sufficient to produce unbiased entropy estimates by applying the quadratic extrapolation correction described in ref. [S1] (their Figure 2 inset). Specifically, for every cell’s response to presynaptic stimulation, and for one cell’s response to every dynamic clamp condition, we fit a quadratic polynomial [S1] describing how

the entropy estimate converges when using increasing fractions of the dataset (Figure S2B inset). The correction (extrapolated value minus value obtained using the whole data set) averaged over the 10 cells for synaptic stimulation was only 1.3 ± 0.4% for the total entropy and 0.6 ± 0.3% for the noise entropy, and averaged over the 7 g_{syn} values in one specimen cell was only 1.0 ± 0.2% for the total entropy and 0.4 ± 0.4% for the noise entropy. We therefore used the empirical values (based on the entire dataset) rather than the quadratically extrapolated values for both total and noise entropies.

For the noise entropy, using 5 repetitions of the same input undersamples the distribution of words for word lengths of 3 bins (9 msec) or longer. Nevertheless, the relationship between synaptic energetic efficiency and g_{syn} was independent of the word length used for the analysis (Figure S3), and was replicated by a different method of calculating mutual information which avoids this undersampling problem (Figure S5D). For this second method, we segmented the 125 second input train into a series of 3 ms time bins (approximately the refractory period of a neuron) and calculated the information contained in this train (I_{input}) as:

$$I_{input} = - s.\log_2(s) - (1 - s).\log_2(1 - s)$$

where s is the probability of an action potential arriving in any given time bin, and I_{input} is given in bits per time bin [S2, S3 and S4]. We then calculated the mutual information per time bin (I_m), i.e. how much the sequence of output spikes measured in the LGN cell (AP_{out}) tells us about the train of input spikes (AP_{in}):

$$I_m(APs_{out}; APs_{in}) = I_{input} + \Sigma\ p(y)\ .\ \Sigma\ p(x|y)\ .\ \log_2\ [p(x|y)]$$

where the input x is 1 when there is a spike and 0 otherwise, the output y is 1 when there is a spike and 0 otherwise, and the sums are over all x and y (ref. [S2], eqn. 4.12). In order to be counted as being triggered by an input spike, an output spike had to occur within a particular time window after the input spike. This time window was set to 18 ms based on post-stimulus time histograms of the experimental data (Figures S5A-S5C), and probability distributions were built for each recording, for each of four possible occurrences (x-y): (1) an output spike preceded by an input spike (1-1); (2) an output spike not preceded by an input spike (“0-1”); (3) no output spike when there was an input spike (“1-0”); (4) no output spike when there was not an input spike (“0-0”). Results of this calculation were divided by the time bin (3 ms) to get information rates in bits/sec. Applying this method gave information estimates that were almost identical to those calculated using the direct method, and the choice of information analysis method did not affect the energy efficiency curve (Figure S5D).

Mathematical model of thalamic relay cells

The mathematical model of thalamic relay cells was adapted from earlier models [S5, S6 and S7]. Briefly, the model LGN neuron consisted of a single compartment that included voltage-dependent currents described by Hodgkin-Huxley kinetics [S8]

$$C_m \frac{dV}{dt} = -\sum_j i_j - i_{Hold} - i_{Syn}$$

where C_m = 1 μF/cm^2 is the membrane capacitance, V is the membrane voltage (in mV), i_{Hold} is the injected current, i_{Syn} is the synaptic current and i_j are the intrinsic currents. The cell surface area was 1.52·10^{-4} cm^2, derived from the measured cell capacitance of 152±8 pF (mean±s.e.m., n=18) by assuming a specific capacitance of 1 μF/cm^2. All currents and conductances are subsequently reported per unit surface area (cm^2). Following Bazhenov and colleagues [S6], the intrinsic currents included a leak current i_L, a potassium leak current i_{KL}, an A-type potassium current i_A, a T-type low threshold calcium current i_T, an h-current i_h, a fast sodium current i_{Na} and a fast potassium current i_K. All the intrinsic currents had the same general form

$$i = g m^M h^N (V - E)$$

where for each current i, g is the maximal conductance, $m(t)$ is the activation variable, $h(t)$ is the inactivation variable, E is the reversal potential and M and N are the number of independent activation and inactivation gates.

The i_h current was given by

$$i_h = g_{\max}\, O\, (V - E_h)$$

with E_h = –43 mV [S9]. $g_{\max}$ = 0.0254 mS/cm^2 was set to match the average current recorded in our experiments in response to a 200 ms hyperpolarizing voltage-clamp pulse from –60 to –120 mV. The time dependence of the gating variable O was defined by

$$\frac{dO}{dt} = \frac{1}{\tau_O}(O_\infty - O)$$

with time constant $\tau_O = 1/\left[e^{(-14.59-0.086\cdot V)} + e^{(-1.87+0.0701\cdot V)}\right]$ (in msec) and steady-state variable $O_\infty = 1/\left[1 + e^{((V+75)/5.5)}\right]$ [S9].

The leak currents were given by

$$i_L = g_{\mathrm{L}}(V - E_L)$$

and

$$i_{KL} = g_{\mathrm{KL}}(V - E_K)$$

with E_L = –70 mV [S5]. E_K was set to match the effective potassium reversal potential used in the experiments E_K = –105 mV, while g_{L} = 0.025 mS/cm^2 and g_{KL} = 0.025 mS/cm^2 were manually adjusted to match both the average input resistance at the resting membrane potential (R_i = 149±28 MΩ in experiments, mean ± s.e.m., n=18; R_i = 145 MΩ in the model) and the resting membrane potential as recorded in experiments (V_{rest} = -76±2 mV in experiments, mean ± s.e.m., n=18; V_{rest} = –77.4 mV in the model).

The A-type potassium current was given by

$$i_A = g_A m^M h^N (V - E_K)$$

with M = 4 and N = 1. The time dependence for m and h was defined as for O, with

$$m_\infty = 1/\left[1 + e^{(-(V+60)/8.5)}\right]$$

$$\tau_m = 0.1 + 0.27/\left[e^{((V+35.8)/19.7)} + e^{(-(V+79.7)/12.7)}\right]$$

$$h_\infty = 1/\left[1 + e^{((V+78)/6)}\right]$$

and

$$\tau_h = 0.27/\left[e^{((V+46)/5)} + e^{(-(V+238)/37.5)}\right]$$

if $V < -63$ mV and $\tau_h = 5.1$ msec otherwise [S6 and S7].

The T-type calcium current was given by

$$i_T = g_T m^M h^N (V - E_T)$$

with M = 2 and N = 1. The time dependence for m and h was defined as for O, with

$$m_\infty = 1/\left[1 + e^{(-(V+57)/6.2)}\right]$$

$$\tau_m = 0.13 + 0.22/\left[e^{(-(V+132)/16.7)} + e^{((V+16.8)/18.2)}\right]$$

$$h_\infty = 1/\left[1 + e^{((V+83)/4)}\right]$$

and

$$\tau_h = 8.2 + \left[56.6 + 0.27 \cdot e^{((V+115.2)/5)}\right]/\left[1 + e^{((V+86)/3.2)}\right]$$

E_T is given by $E_T = RT/2F \cdot \log(\mathrm{Ca}_0^{2+}/\mathrm{Ca}^{2+})$ with F = 96489 C/mol the Faraday constant, R = 8.314 J mol^{-1} K^{-1} the gas constant, T = 309°K the temperature and Ca_0^{2+} = 2 mM the extracellular calcium concentration. The intracellular calcium dynamics were defined by

$$\frac{d\mathrm{Ca}^{2+}}{dt} = -\frac{1}{\tau_{\mathrm{Ca}}}\left(\mathrm{Ca}^{2+} - \mathrm{Ca}_i^{2+}\right) - A\, i_T$$

with Ca_i^{2+} = 2.4·10^{-4} mM, the baseline intracellular calcium concentration, and A = 5.18·10^{-5} mM cm^2 msec^{-1} µA^{-1}, a constant.

The fast sodium current was defined by

$$i_{Na} = g_{Na} m^3 h (V - E_{Na})$$

with E_{Na} = +90 mV adapted to match the experiments. The maximal conductance g_{Na} = 4.4 mS/cm^2 was set to match the peak current recorded in experiments in response to a 200 ms +40 mV depolarizing voltage step from the resting membrane potential. The time dependence for m and h was defined by

$$\frac{dx}{dt} = \alpha_x(1 - x) - \beta_x x$$

where x stands for either h or m and with [S10]

$$\alpha_m = 0.32\left[13.1 - V + V_{\text{shift}}^{\text{Na}}\right] / \left[e^{\left((13.1 - V + V_{\text{shift}}^{\text{Na}})/4\right)} - 1\right]$$

$$\beta_m = 0.28\left[V - V_{\text{shift}}^{\text{Na}} - 40.1\right] / \left[e^{\left((V - V_{\text{shift}}^{\text{Na}} - 40.1)/5\right)} - 1\right]$$

$$\alpha_h = 0.128\, e^{\left((17 - V + V_{\text{shift}}^{\text{Na}})/18\right)}$$

and

$$\beta_h = 4/\left[1 + e^{\left((40 - V + V_{\text{shift}}^{\text{Na}})/5\right)}\right].$$

The fast potassium current was given by

$$i_K = g_K n^4 (V - E_K)$$

The maximal conductance g_K = 3.3 mS/cm^2 was set to match the steady-state current recorded in experiments in response to a 200 ms +40 mV depolarizing voltage step from the resting membrane potential. The time dependence for n was defined as for the sodium gating variables m and h with [S10]

$$\alpha_n = 0.032\left[15 - V + V_{\text{shift}}^{\text{K}}\right] / \left[e^{\left((15 - V + V_{\text{shift}}^{K})/5\right)} - 1\right]$$

and

$$\beta_n = 0.5\, e^{\left((10 - V + V_{\text{shift}}^{\text{K}})/40\right)}.$$

$V_{\text{shift}}^{\text{Na}}$ = –60.1 mV and $V_{\text{shift}}^{\text{K}}$= –62.5 mV were manually adjusted to allow the model to be depolarized to –55 mV without spontaneously spiking. These values slightly differ from the values in the model of ref. [S10] ($V_{\text{shift}}^{\text{Na}}$ = $V_{\text{shift}}^{\text{K}}$= –60 mV) and from the values in the model of refs. [S6] and [S7] ($V_{\text{shift}}^{\text{Na}}$ = $V_{\text{shift}}^{\text{K}}$= –63 mV). i_{Hold} = -2.05 µA/cm^2 was set in subsequent simulations so as to hold the model at –55 mV. For a cell surface area of $1.52 \cdot 10^{-4}$ cm^2, this corresponds to an injected current of ~310 pA, similar to experimentally measured values of 30-550 pA. g_A = 3 mS/cm^2 and g_T = 1.8 mS/cm^2 were set so that the model achieved an output frequency, when stimulated with the recorded synaptic conductance, similar to the average frequency observed in experiments with extracellular stimulation (5.3 Hz in the model, 4.1±0.8 Hz in the experiments, mean±s.e.m., n=18).

To determine the AMPA and NMDA synaptic conductance components generated by extracellular stimulation of retinal ganglion cell axons with the 125 second stimulus trains used in Figs. 3 to 5, we recorded the evoked currents in thalamic relay neurons held at -55 mV with and without bath application of the NMDAR blocker 50 µM D-AP5 (in the presence of 5 µM GABAzine). Currents were converted to conductance by dividing them by the holding potential (i.e. using a reversal potential of 0 mV). To determine the time course of the NMDA component, the conductance time course recorded in AP5 was subtracted from the total conductance time course. This procedure was repeated with three cells and the average AMPA (g_{AMPA}) and NMDA (g_{NMDA}) conductance time courses (Figure S4A) that were evoked by the input stimulation trains were used as inputs in the

simulations. To reproduce the dynamic-clamp experiments modulating the amplitude of the synaptic conductance, g_{AMPA} and g_{NMDA} were scaled by a gain factor varying between 0 and 12. Two batches of simulations were run, one with a linearized NMDA I-V relation, so that the total synaptic current varied linearly with voltage, as was used in the experiments, and one with a voltage-dependent NMDA conductance including Mg^{2+} block, to investigate whether our omission of this non-linearity in the dynamic clamp experiments had a significant effect on the results. In the first case, the synaptic current i_{Syn} is simply given by

$$i_{Syn} = -(g_{AMPA} + g_{NMDA})\,(V - E_{\mathrm{excitatory}})$$

with $E_{\mathrm{excitatory}}$ = 0 mV. To describe the NMDA non-linear I-V relation, we recorded the evoked currents in thalamic relay neurons generated by retinal ganglion cell stimulation during bath application of 1 µM NBQX (in the presence of 5 µM GABAzine) while holding the cell at different voltages (-74 mV to 36 mV in 10 mV steps). The recorded currents were then averaged across cells, and the voltage dependence was fitted by a function of the form $f(V) = a/(1 + b \cdot e^{-c \cdot V})$ with a, b and c as constants. i_{Syn} was then given by

$$i_{Syn} = -g_{\mathrm{AMPA}}(V - E_{\mathrm{excitatory}}) - g_{\mathrm{NMDA}}\left(\frac{9.69}{1 + 0.1688\, e^{-0.0717\,V}}\right)(V - E_{\mathrm{excitatory}})$$

where $g_{\mathrm{AMPA}}(V\text{-}E_{\mathrm{excitatory}})$ and $g_{\mathrm{NMDA}}(V\text{-}E_{\mathrm{excitatory}})$ describe the time course of the separate synaptic current components at -55 mV.

To calculate information transfer at the simulated synapse (Figure S4B-D), output spike trains were processed exactly as described for the experimental data. To calculate the metabolic cost incurred by the modelled cell, for Figures S4E and S4F the Na^+ component of i_{Syn} was integrated and converted to the corresponding ATP consumption per unit time, while for Figure S4G the same procedure was followed for i_{Na}, the Na^+ component of i_h and Ca^{2+} entry via i_T, and this was added to the ATP used on i_{Syn}. For i_{Syn}, the conductance was scaled by 7/13 (derived from the reversal potentials $E_{\mathrm{excitatory}}$ = 0 mV, E_{Na} = +90 mV and E_K = -105 mV) and multiplied by V - E_{Na} to calculate the contribution of sodium ions. For i_h, the conductance was scaled by $(E_K - E_h)/(E_K - E_{Na})$ and multiplied by V - E_{Na} to isolate the contribution of sodium ions. For i_T, we assumed that each calcium ion is exchanged for 3 sodium ions [S11].

To convert currents and conductance back and forth between absolute amplitudes (measured) and amplitudes per surface area, we used the average cell surface area taken from membrane capacitance measurements obtained in experiments ($1.52 \cdot 10^{-4}$ cm^2; see above). Simulations were run using custom-written MATLAB scripts (The Mathworks, Natick MA). Differential equations were integrated using the built-in solver *ode15s* with an integration time step dt = 0.05 ms.

Tonic and burst modes of thalamic firing

Depending on their resting potential, relay neurons in the thalamus have two modes of firing: tonic mode (at -55 mV), where a single input spike tends to produce (at the most) one output spike, and burst mode (below -70 mV), where a single input spike may produce a burst of output spikes [S12], riding on a depolarising "calcium spike" (Figure S5E). Our analysis is restricted to cells firing in the tonic mode seen during alert wakefulness [S13 and S14]. Preliminary data from 3 cells (not shown) suggested that the same optimisation of information transmitted per energy used occurs during burst mode, however the synaptic energy efficiency may differ during less alert states or sleep, when synapses may renormalise their postsynaptic conductances and restore their energy supply [S15, S16 and S17]. A complication with carrying out this analysis in burst mode is the uncertainty in how information should be calculated: should bursts be treated as unitary events (in which case, they have been found to carry as much as three times the information of single spikes in tonic mode [S18]) or should the spikes within the bursts be treated as independent events, as we have done for tonic mode (in which case, bursts have been reported to contribute negatively to information transfer at the retinogeniculate synapse [S19])? A better understanding of which burst features are most relevant to coding at this synapse will be critical to assessing the energetic efficiency of information transmission in burst mode.

Effects of local inhibition at the retinogeniculate synapse

In vivo, the retinogeniculate synapse does not act in isolation, but in the presence of GABAergic modulation (both pre- and post-synaptically [S20, S21 and S22]). Inhibition evoked by retinal input has been suggested, in the cat, to generate net hyperpolarization of the LGN cell and control the transition from tonic firing to burst firing mode [S23]. We never saw this behaviour in our recordings (optic tract stimulation always evoked excitation followed by disynaptic inhibition, rather than net inhibition), possibly because of our use of rat rather than cat, or the absence of anaesthesia in our brain slice experiments, but inhibition might nevertheless shape the cells' coding properties. Indeed, in principle, inhibition might improve the temporal precision of postsynaptic action potentials and thus increase the information encoded [S24].

We therefore performed the retinogeniculate experiments in the absence of gabazine, and found that approximately half of patch-clamped relay neurons had observable disynaptic inhibition, which tended to display short-term depression (Figure S1E). Surprisingly, we found that blocking the disynaptic inhibition in these cells by superfusing gabazine did not alter the firing frequency or information rate when presynaptic stimulation was applied (Figure S1F). Because gabazine also has no effect on EPSC size (Figure S1F), it appears that local inhibition does not significantly affect the energy efficiency of retinogeniculate transmission.

Correlations between inputs

In vivo, especially in sensory systems, it is likely that a set of postsynaptic neurons will receive inputs from presynaptic cells that are transmitting correlated information. An important future

line of work will be to assess the energetic efficiency of the whole population of retinal synapses onto the whole set of thalamic neurons. For example, the correlated activity of multiple cells may [S25], or may not [S26], increase the amount of information available to the visual cortex. When assessing how synaptic conductances are set to regulate information transfer and energy use, it will be interesting to investigate whether the conductance of each input to a postsynaptic cell is set independently, or whether account is taken of the correlations in information passing through spatially adjacent cells.

Relationship to earlier work

An earlier theoretical analysis claimed that the information transmitted at excitatory synapses is maximized per molecule of glutamate released [S27]. However, as has previously been noted (https://pubpeer.com/publications/23242311), this paper confused the two uses of the term entropy in information theory. If a signalling system can represent signals in many different ways (implying a high signal entropy), then it can transmit a large amount of information. However, if there is a lot of random noise in the signal then there will be a large noise entropy. Unfortunately the calculations in ref. [S27] maximise the noise entropy (by maximising the variations of postsynaptic current generated by random opening and closing of ion channels) - they do not maximise the signal entropy. Thus, the claim [S27] that the observed number of glutamate molecules maximises the signal information content is entirely the opposite of what their analysis does, which is to maximise the noise.

SUPPLEMENTAL REFERENCES

S1. Strong, S.P., Koberle, R., de Ruyter van Steveninck, R., and Bialek, W. (1998). Entropy and Information in Neural Spike Trains. Phys. Rev. Lett. *80*, 197-200.

S2. Dayan, P., and Abbott, L.F. (2001). Theoretical Neuroscience (Cambridge, Massachusetts: The MIT Press).

S3. Shannon, C.E. (1948). A mathematical theory of communication. Bell System Tech. J. *27*, 379-423.

S4. Levy, W.B., and Baxter, R.A. (1996). Energy efficient neural codes. Neural Comput. *8*, 531-543.

S5. McCormick, D.A., and Huguenard, J.R. (1992). A model of the electrophysiological properties of thalamocortical relay neurons. J. Neurophysiol. *68*, 1384-1400.

S6. Bazhenov, M., Timofeev, I., Steriade, M., and Sejnowski, T.J. (1998a). Cellular and network models for intrathalamic augmenting responses during 10 Hz stimulation. J. Neurophysiol. *79*, 2730-2748.

S7. Bazhenov, M., Timofeev, I., Steriade, M., and Sejnowski, T.J. (1998b). Computational models of thalamocortical augmenting responses. J. Neurosci. *18*, 6444-6465.

S8. Hodgkin, A.L., and Huxley, A.F. (1952). A quantitative description of membrane current and its application to conduction and excitation in nerve. J. Physiol. *117*, 500-544.

S9. Huguenard, J.R., and McCormick, D.A. (1992). Simulation of the currents involved in rhythmic oscillations in thalamic relay neurons. J. Neurophysiol. *68*, 1373-1383.

S10. Traub, R.D., and Miles, D. (1991). Neuronal networks of the hippocampus (Cambridge: CUP).

S11. Attwell, D., and Laughlin, S.B. (2001). An energy budget for signaling in the grey matter of the brain. J. Cereb. Blood. Flow Metab. *21*, 1133-1145.

S12. Scharfman, H.E., Lu, S.M., Guido, W., Adams, P.R., and Sherman, S.M. (1990). N-methyl-D-aspartate receptors contribute to excitatory postsynaptic potentials of cat lateral geniculate neurons recorded in thalamic slices. Proc. Natl. Acad. Sci. U.S.A. *87*, 4548-4552.

S13. Coenen, A.M., and Vendrik, A.J. (1972). Determination of the transfer ratio of cat's geniculate neurons through quasi-intracellular recordings and the relation with the level of alertness. Exp. Brain Res. *14*, 227-242.

S14. Livingstone, M.S., and Hubel, D.H. (1981). Effects of sleep and arousal on the processing of visual information in the cat. Nature *291*, 554-561.

S15. Maret, S., Faraguna, U., Nelson, A.B., Cirelli, C., and Tononi, G. (2011). Sleep and waking modulate spine turnover in the adolescent mouse cortex. Nat. Neurosci. *14*, 1418-1420.

S16. Vyazovskiy, V.V., Cirelli, C., Pfister-Genskow, M., Faraguna, U., and Tononi, G. (2008). Molecular and electrophysiological evidence for net synaptic potentiation in wake and depression in sleep. Nat. Neurosci. *11*, 200-208.

S17. Harris, J.J., Jolivet, R., and Attwell, D. (2012). Synaptic energy use and supply. Neuron *75*, 762-777.

S18. Reinagel, P., Godwin, D., Sherman, S.M., and Koch, C. (1999). Encoding of visual information by LGN bursts. J. Neurophysiol. *81*, 2558-2569.

S19. Reinagel, P., and Reid, R.C. (2000). Temporal coding of visual information in the thalamus. J. Neurosci. *20*, 5392-5400.

S20. Turner, J.P., and Salt, T.E. (1998). Characterization of sensory and corticothalamic excitatory inputs to rat thalamocortical neurones in vitro. J. Physiol. *510*, 829-843.

S21. Chen, C., and Regehr, W.G. (2000). Developmental remodeling of the retinogeniculate synapse. Neuron *28*, 955-966.

S22. Chen, C., and Regehr, W.G. (2003). Presynaptic modulation of the retinogeniculate synapse. J. Neurosci. *23*, 3130-3135.

S23. Wang, X., Hirsch, J.A., and Sommer, F.T. (2010). Recoding of sensory information across the retinothalamic synapse. J. Neurosci. *30*, 13567-13577.

S24. Butts, D.A., Weng, C., Jin, J., Yeh, C., Lesica, N., Alonso, J., and Stanley, G.B. (2007). Temporal precision in the neural code and the timescales of natural vision. Nature *449*, 92-96.

S25. Dan, Y., Alonso, J.M., Usrey, W.M., and Reid, R.C. (1998). Coding of visual information by precisely correlated spikes in the lateral geniculate nucleus. Nat. Neurosci. *1*, 501-507.

S26. Meytlis, M., Nichols, Z., and Nirenberg, S. (2012). Determining the role of correlated firing in large populations of neurons using white noise and natural scene stimuli. Vision Res. *70*, 44-53 .

S27. Savtchenko, L.P., Sylantyev S., and Rusakov D.A. (2013). Central synapses release a resource-efficient amount of glutamate. Nat. Neurosci. *16*, 10-12.
