# Supplementary material for: Energy-Efficient Information Transfer by Visual Pathway Synapses
Source: Curr Biol. 2015 Dec 21;25(24):3151–60. doi: 10.1016/j.cub.2015.10.063 (PMC4691239; doi:10.1016/j.cub.2015.10.063)
Supplement: Document S2. Article plus Supplemental Information [file mmc2.pdf]

Article

Current Biology

Energy-Efficient Information Transfer by Visual Pathway Synapses

Highlights

- How do synapses optimize energy consumption on information transmission?
- We find that increasing synaptic current size increases information transmission
- But larger or smaller EPSCs decrease the information transmitted per energy used
- Thus, the biologically observed EPSC size maximizes synapse energy efficiency

Authors

Julia J. Harris, Renaud Jolivet, Elisabeth Engl, David Attwell

Correspondence

d.attwell@ucl.ac.uk

In Brief

Synapses transmit information between neurons, but the energetic cost of postsynaptic currents is high. How are synaptic properties set to balance this trade-off? Harris et al. show that thalamic relay synapse properties are tuned to maximize not bits of information transmitted per second but bits of information transmitted per ATP molecule used.

CrossMark

Harris et al., 2015, Current Biology *25*, 3151–3160
December 21, 2015 ©2015 The Authors
http://dx.doi.org/10.1016/j.cub.2015.10.063

CellPress

Current Biology
Article

CellPress

Energy-Efficient Information Transfer by Visual Pathway Synapses

Julia J. Harris,[1,2,3] Renaud Jolivet,[1,2] Elisabeth Engl,[1] and David Attwell[1,*]
[1]Department of Neuroscience, Physiology and Pharmacology, University College London, Gower Street, London WC1E 6BT, UK
[2]Co-first author
[3]Present address: Department of Life Sciences, Imperial College London, South Kensington Campus, London SW7 2AZ, UK and Francis Crick Institute, Mill Hill Laboratories, London NW7 1AA, UK
*Correspondence: d.attwell@ucl.ac.uk
http://dx.doi.org/10.1016/j.cub.2015.10.063
This is an open access article under the CC BY license (http://creativecommons.org/licenses/by/4.0/).

SUMMARY

The architecture of computational devices is shaped by their energy consumption. Energetic constraints are used to design silicon-based computers but are poorly understood for neural computation. In the brain, most energy is used to reverse ion influxes generating excitatory postsynaptic currents (EPSCs) and action potentials. Thus, EPSCs should be small to minimize energy use, but not so small as to impair information transmission. We quantified information flow through the retinothalamic synapse in the visual pathway in brain slices, with cortical and inhibitory input to the postsynaptic cell blocked. Altering EPSC size with dynamic clamp, we found that a larger-than-normal EPSC increased information flow through the synapse. Thus, the evolutionarily selected EPSC size does not maximize retinal information flow to the cortex. By assessing the energy used on postsynaptic ion pumping and action potentials, we show that, instead, the EPSC size optimizes the ratio of retinal information transmitted to energy consumed. These data suggest maximization of information transmission per energy used as a synaptic design principle.

INTRODUCTION

The geometry of excitatory synapses is subject to competing constraints. Synapse diameter needs to be small, first so that a neuronal dendrite can receive a large number of synaptic inputs and second because if synapses are too large in diameter then glutamate clearance by diffusion to surrounding astrocytes will be too slow, limiting the maximum rate of information transfer through the synapse [1]. On the other hand, if synapses are too small and possess only a few glutamate receptors, then variability in the opening of postsynaptic ion channels creates noise in the postsynaptic signal [2, 3]. Similarly, because most brain energy is used to pump out ions that enter through postsynaptic receptors [4, 5], the number of receptors per synapse should be kept small to minimize energy use, but if it is too small, the postsynaptic effect of the input will be negligible. How have excitatory synapses evolved to cope with these competing requirements?

We studied the lateral geniculate nucleus relay synapse in the visual system to investigate whether postsynaptic currents are large, to transmit to the cortex as much as possible of the information arriving from the retina, or smaller, to save energy. Surprisingly, increasing synaptic conductance beyond the biological norm allows more information flow across the synapse, showing that synapse properties are not set to maximize information transfer. Instead, analysis of the energetic cost of the postsynaptic ion pumping associated with synaptic signaling [6] revealed that synapse properties are evolutionarily selected to maximize the information transferred per energy used. In other words, synapses do not maximize bits transmitted per second but bits transmitted per ATP molecule.

Theoretical analysis has previously shown that the low mean firing rate of neurons [7] and the surprisingly low release probability of central synapses [4] can be explained if axons and presynaptic terminals operate to maximize the information transmitted per energy used. The results presented below extend this concept to postsynaptic terminals, the largest consumers of energy in the brain, and are consistent with energy use profoundly constraining the operation of the CNS.

RESULTS

Spike Transmission through the LGN Relay Synapse

We stimulated optic tract axons making synapses onto whole-cell patch-clamped dorsal LGN relay neurons in the thalamus of rat brain slices [8] (Figures 1A and S1) with ganglion cell responses to natural visual scenes [9]. The relay neurons were held in the tonic firing mode (at −55 mV) seen during alert wakefulness in vivo, where a single input spike tends to produce (at the most) one output spike, as opposed to the burst mode (below −70 mV) occurring during less-alert states or sleep, where a single input spike may produce a burst of output spikes (see Supplemental Information and Figure S5E). To isolate a single excitatory input from the optic tract, we used animals at an age (P28) when the retinogeniculate connection is mature and one retinal ganglion cell makes a giant glomerular synapse with many release sites onto one LGN relay neuron [10, 11], we cut off the cortex, and we blocked $GABA_A$ receptors (Figure 1B) although blocking inhibition had little effect (see Figure S1F and Supplemental Information). The stimulus trains used had a mean spike frequency of ~19 Hz, which evoked postsynaptic firing at a mean rate of ~4 Hz (Figures 1C and 1D). Thus, despite being

CrossMark

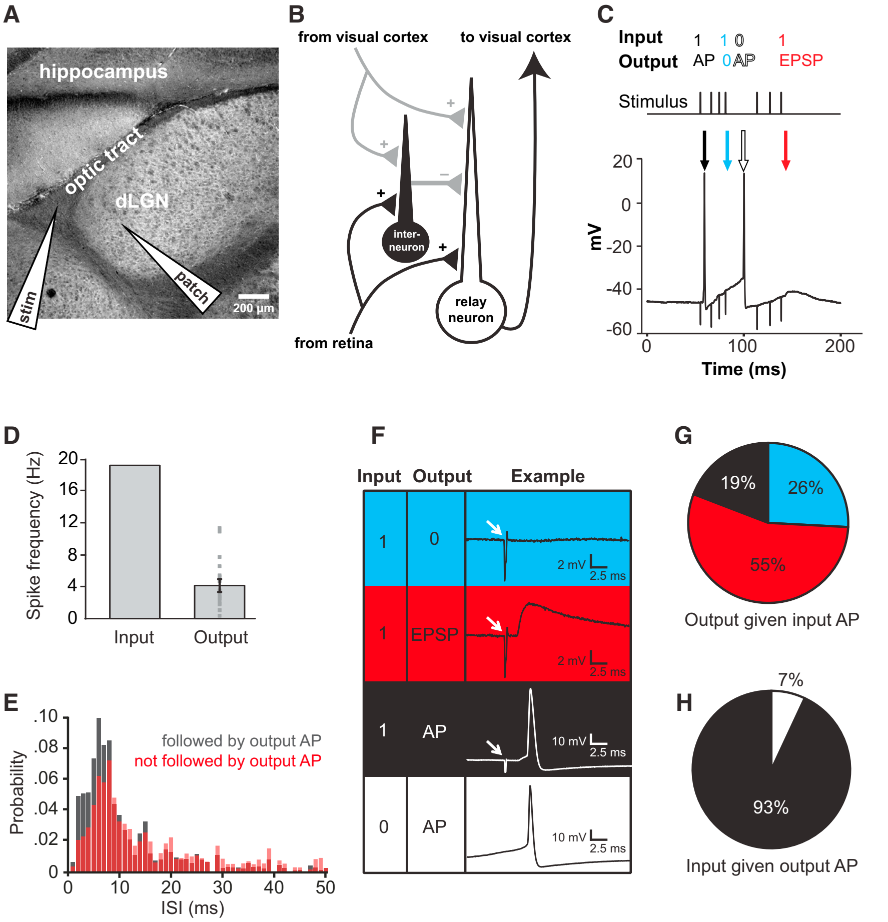

Figure 1. Spike Transmission through the Optic Tract-LGN Synapse

(A) Slice preparation showing the stimulating electrode in the optic tract and the recording electrode in the dorsal LGN.

(B) Circuitry of the LGN with functioning pathways in black (axons from the retina and to the visual cortex) and inactivated pathways in gray (cortex is removed to remove cortical input, and inhibition from interneurons is abolished with gabazine).

(C) Specimen trace showing that the stream of input action potentials (timing shown by stimulus trace) does not reliably generate postsynaptic action potentials.

(D) Spike frequency (mean ± SEM) in the input stimulus train (input) and evoked in 18 LGN cells (output). Points show data from individual cells.

(E) For cases where a postsynaptic action potential did occur (black distribution) or did not occur (red distribution), graphs show the probability of the preceding two presynaptic APs being separated by the interval shown on the abscissa (the area under each distribution is unity).

(F) Logical table stating possible input and output combinations, with specimen examples of each.

(G) Observed outcomes given an input AP (EPSPs had to be larger than 1 mV to be counted).

(H) Observed input APs given an output AP.

See also Figure S1.

designated a “relay synapse,” the optic tract to LGN synapse does not ensure an output spike for every input spike. Instead, as first described [12], the occurrence of two input action potentials close together in time increases the chance of generating a postsynaptic action potential (Figure 1E).

Spike transmission at this synapse has been observed to vary widely (even within the same species and anesthetic state) [12–16], but it is generally agreed that less than 50% of spikes are successfully transmitted (with the average across these papers being 23% ± 8%). We found that only 19% of input action potentials produced an output action potential (Figures 1F and 1G; see Experimental Procedures), whereas 55% produced only an EPSP and 26% produced no detectable EPSP in the following 18 ms (only EPSPs over 1 mV in amplitude were reliably detectable, so this may be an overestimate; see Experimental Procedures for choice of 18 ms). Some apparently spontaneous output action potentials were associated with no input stimulus spike in the preceding 18 ms (Figure 1H).

The input action potential train, which was composed of retinal ganglion cell responses to natural movies (from [9]) carried 94 bits/s of information (quantified using the direct method [17] but with zero noise entropy). The output spike train recorded in relay neurons carried roughly one-fifth of this information (18.3 bits/s). However, the number of bits of information encoded per action potential was not significantly affected by transmission through the synapse: the input train encoded 4.9 bits/spike (94 bits/s with a mean firing rate of 19.0 Hz), which is slightly higher than the 1.5–3.5 bits/spike found for natural stimuli in guinea pig retinal ganglion cells [18], whereas on average, the output train evoked by synaptic input encoded 4.7 bits/action potential (18.3 ± 4.5 bits/s at 3.9 ± 1.1 Hz), which is similar to a previous report of 3.6 bits/action potential in cat LGN cells [19].

Relationship between Synaptic Conductance and Spike Output

How does the reduction in mean spike rate at the LGN synapse affect the amount of information transmitted, and how is information flow affected by the size of the postsynaptic conductance evoked by presynaptic glutamate release? Although not all retinal spikes are transmitted across the synapse, those that are relayed are more informative about the visual stimulus than those that fail to be transmitted [13, 14]. Does the relay neuron omit some spikes because they are less informative or because reliably transmitting them would require a larger excitatory postsynaptic current (EPSC) with a correspondingly larger energetic cost? We investigated this by altering the postsynaptic conductance evoked by glutamate release in order to increase or decrease the proportion of retinal spikes that are transmitted and examining the effects on information transmission and postsynaptic energy use.

We recorded the sequence of EPSCs evoked by the input action potential train (Figures 2A and 2B) and examined (in current clamp mode) the resulting action potential train that these EPSCs generated (Figure 2C). After converting the EPSCs to conductance changes, we used dynamic clamp [20] to inject

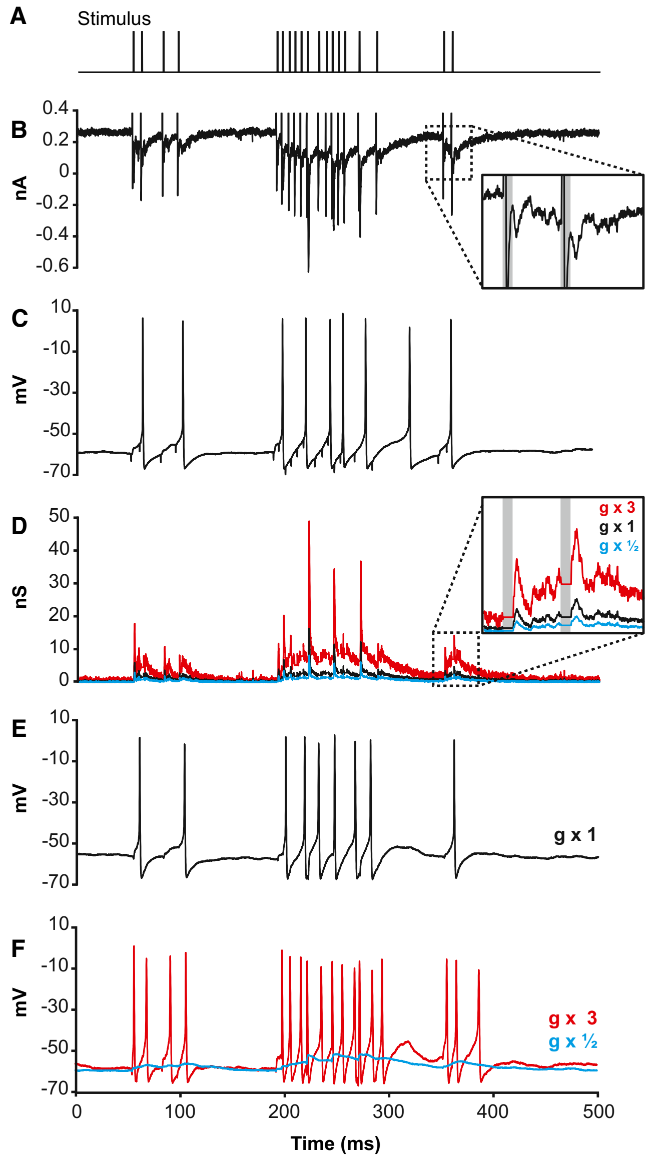

Figure 2. Conversion of Synaptic Conductance to Action Potentials in the LGN

(A) A section of the stimulus train applied to the optic tract axon.

(B) The EPSC train evoked in the LGN cell by the input train when voltage clamped at −55 mV.

(C) The AP sequence evoked in current clamp mode by the train in (A). Large vertical deflections in (B) (gray in inset) and small downward deflections in (C) are stimulus artifacts.

(D) The EPSC conductance time course derived from (B) for injection by dynamic clamp, with the same amplitude as evoked by synaptic input (g × 1) and scaled up and down in size (g × 3; g × ½).

(E) AP stream evoked by dynamic clamp injection at the soma of the g × 1 conductance trace in (D).

(F) AP stream evoked by dynamic clamp injection of the g × 3 and g × ½ conductance traces in (D).

All data are from the same cell.

into the cell soma the recorded conductance scaled up or down by different factors (see Experimental Procedures), so that we could examine the voltage response that would be produced by a larger or smaller synaptic conductance. The dynamic clamp technique uses a computer interface to calculate how much current needs to be injected into the cell to mimic the synaptic conductance while the membrane potential is changing. Figure 2D shows the scaled conductance time course, derived from the current trace in Figure 2B after removal of the stimulus artifact (see Experimental Procedures), for a range of scaling factors. For a synaptic conductance time course injected by dynamic clamp at the soma, with a magnitude equal to that recorded in voltage clamp for a real synaptic input, the resulting action potential response (Figure 2E) was similar to that evoked by the real synaptic input to the cell (Figure 2C), with a similar mean firing rate (4.2 ± 1.0 Hz in dynamic clamp; 3.9 ± 1.1 Hz with synaptic input; ten cells; not significantly different; $p = 0.75$). Scaling the conductance time course evoked by the input signal up or down led to the recorded neuron generating more or fewer spikes, respectively (Figure 2F).

Information Transmission

To quantify the information transmitted across the synapse, we measured the mutual information between stimuli and responses using the direct method [17, 19]. Five different spike trains (1–5; Figure 3A) recorded in retinal ganglion cells in response to natural scenes [9] were used as the stimulus input. Optic tract axons were stimulated with these trains in sequence (1-2-3-4-5), five times (Figure S2A). Each relay neuron therefore responded to each train five times (responses are grouped by input train in Figure 3B). The relay neuron responses to the same input train were generally similar, showing that the output spike trains were not very noisy, whereas the responses to different trains were very different, showing that the output spike trains had the capacity for high variability and thus high information content. Mutual information—how much of the input information is transmitted to the output—is the information capacity minus the noise (see Supplemental Information and Figures S2B, S2C, and S3 for full calculations). With presynaptic stimulation, the mutual information (hereafter simply called "information") was 18.3 ± 4.5 bits/s (Figure 3C). Employing dynamic clamp to apply the recorded synaptic conductance at the cell soma gave an output information rate of 20.6 ± 4.6 bits/s, which was not significantly different from that seen with normal synaptic input ($p = 0.59$; Figure 3C).

Energy Use on Postsynaptic Current

We calculated the energy use on the postsynaptic current for each value of the synaptic conductance—either evoked with presynaptic action potentials (Figures 4A–4C) or scaled and injected with dynamic clamp (Figures 4D and 4E)—by calculating the Na^+ entry through the postsynaptic glutamate-gated conductance and then converting this to ATP use, knowing that one ATP molecule is consumed by the Na^+ pump to extrude three Na^+ ions [6] (see Experimental Procedures). Depolarization of the cell by the postsynaptic current, or by the action potential it evokes, reduces slightly the Na^+ entry through postsynaptic channels compared to the situation in which the cell is voltage clamped at its resting potential (Figure 4F). As a result, the energy used on the postsynaptic current increases slightly less than linearly with the effective postsynaptic conductance (see Figure 5D below). Knowing both the information transmitted

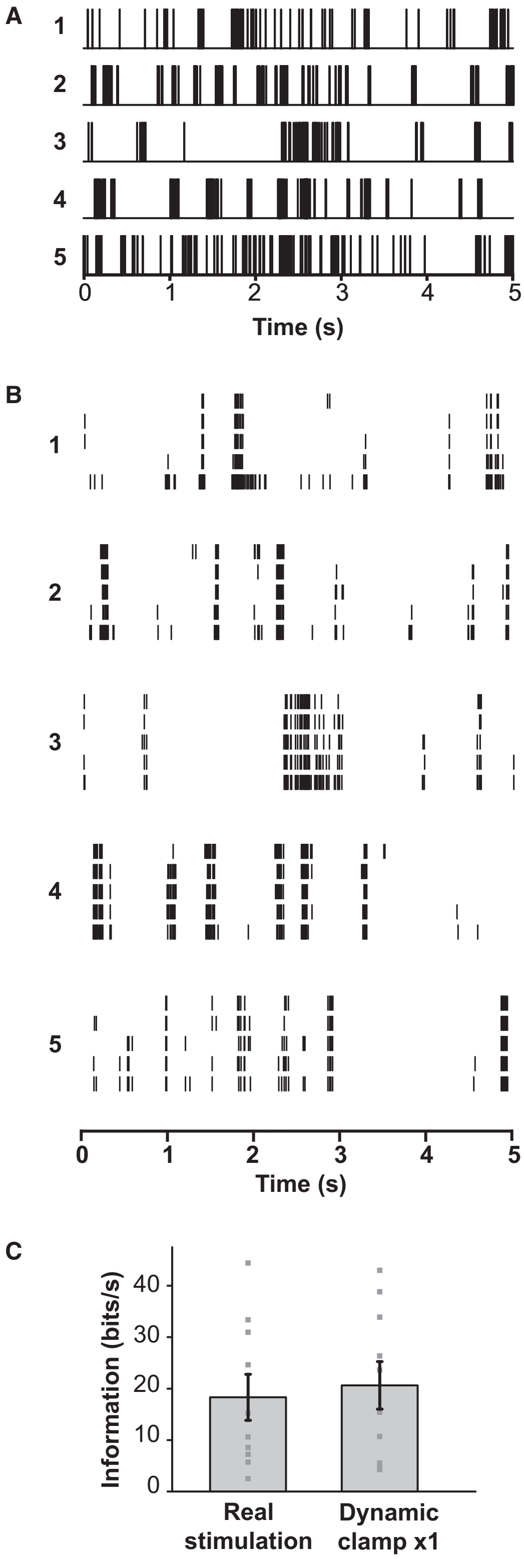

Figure 3. Information Conveyed to the LGN by Natural Scenes

(A) Five-second segments of AP streams (1–5) recorded from ganglion cell axons in response to natural scenes [9].

(B) AP responses (each line is one AP) of a specimen cell to five separate applications of trains 1–5 to the optic tract (real stimulation).

(C) Output information (mean ± SEM) in ten cells (shown as points) when the cell received AP-evoked synaptic currents (real stimulation) or had the measured conductance evoked by real stimulation injected at the soma with the same magnitude (dynamic clamp × 1).

See also Figure S2.

and the energy used, we could then examine the energetic efficiency of the synapse.

Information Transmission and Energy Efficiency at Different Synaptic Conductances

Examining the output information rate as a function of the postsynaptic conductance (scaled up or down using dynamic clamp) showed that a transmitted information rate 4-fold higher than that observed with the biologically occurring synaptic conductance magnitude could occur if the postsynaptic conductance were increased (Figure 5A). Increasing the effective synaptic conductance value tended to increase the mean output firing rate (Figure 5B), although in about 30% of cells at large conductance values a depolarizing block (caused by sodium channel inactivation) set in and the firing rate declined again. The information transmitted increased roughly linearly with firing rate but then reached a plateau (Figure 5C) that was slightly less than the 94 bits/s of information present in the input train.

The data in Figure 5A demonstrate that the magnitude of the postsynaptic conductance is not set so as to maximize information flow through the synapse; indeed only a small fraction of the input information is successfully transmitted. Could this apparently sub-optimal arrangement be due to the large energetic cost of synaptic transmission?

We calculated the ratio of the information transmitted through the synapse (Figure 5A) to the energy used on postsynaptic currents (Figure 5D) when dynamic clamp was used to inject synaptic conductances of different magnitude. Data from individual cells are shown in Figure S3, with the average over ten cells shown in Figure 5E. Strikingly, the ratio of information transmitted to energy used showed a maximum, which was at the physiological conductance value for six of the ten cells and between 0.5 and three times the physiological value for a further three cells. Only one cell had a maximum substantially away from the physiological value (at nine times the physiological conductance, although even this cell also had a smaller local maximum at the physiological value). Thus, for most cells (Figure S3), and also for the average over all the cells (Figure 5E), either a decrease or an increase of the synaptic conductance from its physiological value leads to a remarkable decrease in energetic efficiency for the synapse. For example, increasing the synaptic conductance by a factor of 12, which maximizes information transmission through the synapse (Figure 5A), more than halved the number of bits of information transmitted per ATP used (Figure 5E). To estimate the position of the peak of the relationship (the optimum), we fitted a curve with the form $100.g_{syn}/g_{opt}$ for $g_{syn} < g_{opt}$, and $100.\exp(-(g_{syn} - g_{opt})/K)$ for $g_{syn} > g_{opt}$ to data from each cell and varied g_{opt} (and K) to minimize the sum of the squares of the residuals of the fit. The resulting mean value of g_{opt} was 0.90 ± 0.10 (which is not significantly different from 1; $p = 0.33$). Thus, the optimal value of postsynaptic conductance for maximizing the information transmitted

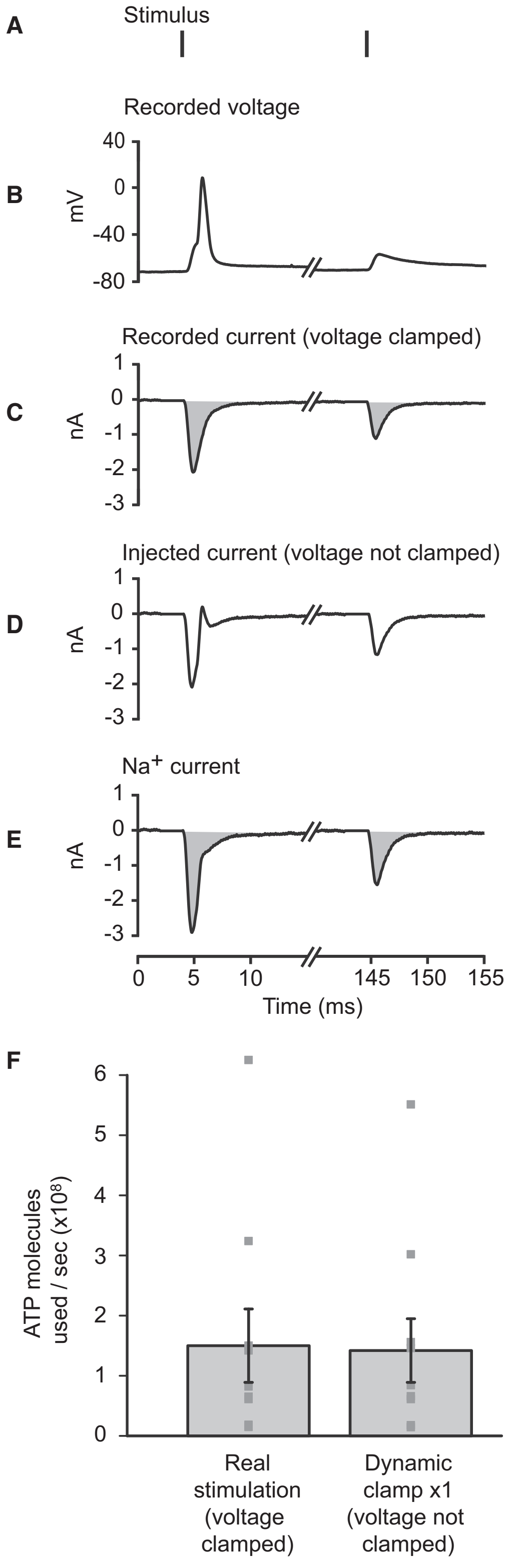

Figure 4. Energy Use on Postsynaptic Currents in the LGN

(A and B) Two stimuli from one of the stimulus trains (A), chosen to evoke (B) an action potential or just an EPSC.

(C) The EPSCs evoked by the stimuli recorded at −55 mV in voltage clamp. The action potential in response to the first stimulus (as shown in B) was evoked by a larger EPSC (note that, because current was recorded in voltage-clamp mode, it does not reflect the sodium influx associated with the action potential itself). Integrating the current trace (area shaded in gray) gives the total postsynaptic charge entry. The actual Na^+ entry is 1.42 times larger than this (see Experimental Procedures). Na^+ entry is then converted to ATP cost at a rate of one ATP molecule per three Na^+ ions.

(D) The synaptic current, calculated from the conductance derived from (C), that is injected in dynamic clamp (with a conductance scaling factor of 1). Because the membrane potential is not voltage clamped, the current shows an outward deflection as the action potential depolarizes the cell positive to the reversal potential for the synapse.

(E) The Na^+ current calculated to occur during dynamic clamping.

(F) The ATP used on extruding Na^+ entering through the postsynaptic conductance, calculated under voltage-clamp conditions during stimulation of the optic tract, and when injecting the same conductance at the soma using dynamic clamp (ten cells; mean ± SEM shown as bar; individual cells shown as points).

per energy used on synaptic currents is not significantly different from the physiologically observed value.

If the postsynaptic current is sufficient to trigger an action potential, an additional energetic cost of restoring ion gradients after the action potential will be incurred. The action potential energy cost per second can be calculated as the product of the cost per action potential (which was $2.05 \pm 0.11 \times 10^7$ ATP molecules for LGN relay neurons; see Experimental Procedures) and the observed firing frequency of the cell. This cost was added onto the synaptic energy cost calculated above, and the information transmitted was calculated relative to the sum of the energy expended on EPSCs and action potentials, as a function of the synaptic conductance scaling factor (Figure 5F). Again, either a decrease or an increase of the synaptic conductance from its physiological value reduced the energetic efficiency of information transmission (Figure 5F). For each cell, we estimated the position of the peak of the relation in Figure 5F, using the same equation as above. The mean value of g_{opt} was 0.75 ± 0.13 (which is not significantly different from 1; $p = 0.08$). Thus, the optimal value of postsynaptic conductance for maximizing the information transmitted per energy used on synaptic currents *and* postsynaptic action potentials is again not significantly different from the physiological value.

Modeling the Energetic Efficiency of Visual Synapses

To check that the membrane currents known to be present in LGN neurons were sufficient to generate the variation of energetic efficiency with synaptic conductance magnitude that is seen in Figure 5, we set up a Hodgkin-Huxley type of mathematical model of these cells, with current amplitudes set to those seen experimentally (see Supplemental Information). For this LGN relay neuron model (Figure S4), we found a dependence of information transfer, energy use, and energetic efficiency on synaptic conductance that was broadly similar to that measured experimentally (Figure 5), with a peak value of information transmitted per energy used at a synaptic conductance close to the normal physiological value.

DISCUSSION

We have examined how information flow through excitatory synapses is related to the size of the conductance activated by glutamate at the postsynaptic terminal and hence to the

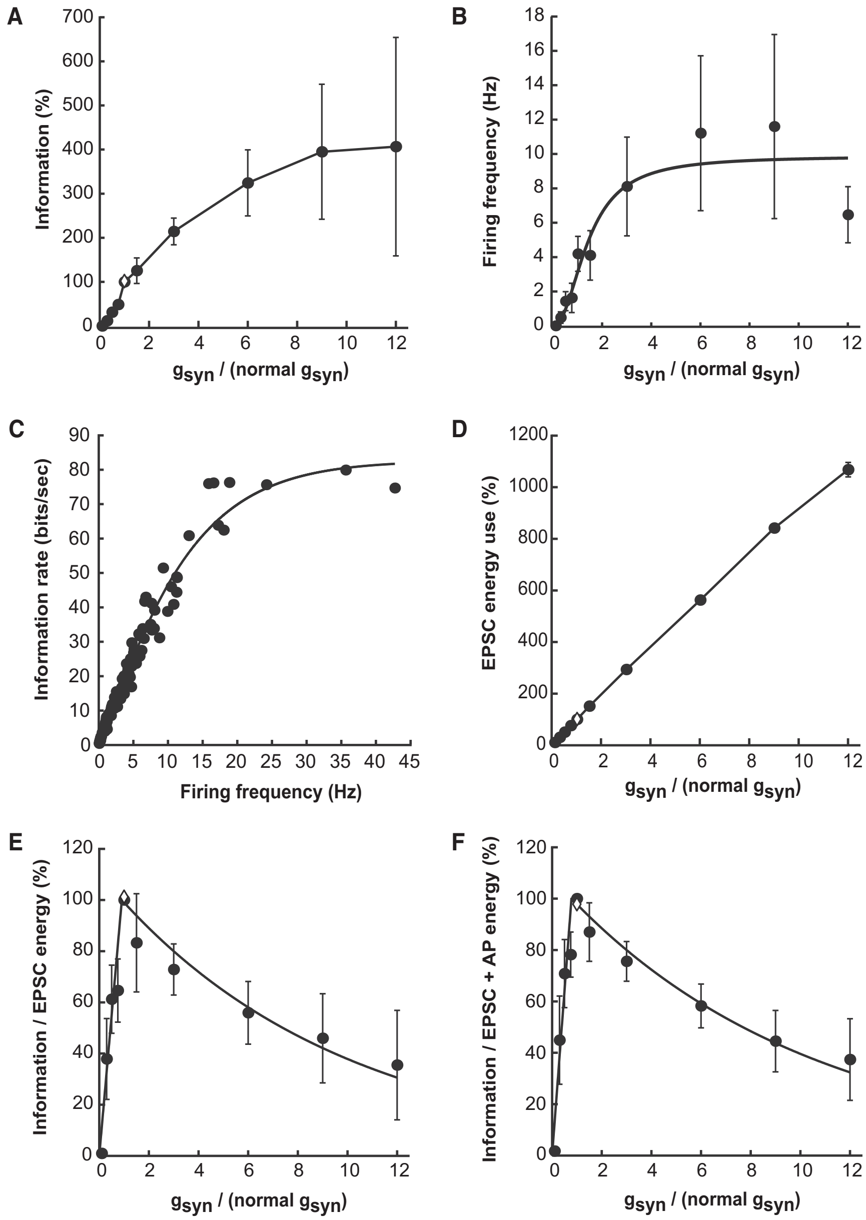

Figure 5. Postsynaptic Conductance Magnitude Maximizes Information Transferred per Energy Used at the RGC-LGN Synapse

(A) Dependence of output information on synaptic conductance (g_{syn}) magnitude, when cells were stimulated with dynamic clamp (black points) with $g_{syn} \times 1$ (applied to all ten cells) and other values (six cells for $g_{syn} \times 0.1$, seven cells for $\times 0.3$, nine cells for $\times 0.5$, three cells for $\times 0.75$, three cells for $\times 1.5$, eight cells for $\times 3$, seven cells for $\times 6$, seven cells for $\times 9$, and four cells for $\times 12$) or with optic tract stimulation (white diamond; ten cells). Information is normalized to the value with $g_{syn} \times 1$, for which the mean information rate was 20.6 ± 4.6 bits/s. Symbols and number of cells per condition are the same in (A) and (D)–(F).

(B) Relationship between firing frequency and g_{syn} for ten cells (fitted equation has the form $F_{max}.g_{syn}{}^n/(g_{syn}{}^n + g_{syn0.5}{}^n)$, where F_{max} = 9.9 Hz, n = 2, and $g_{syn0.5}$ = 1.4).

(C) Dependence of information rate on mean output firing frequency evoked by stimulus trains with different g_{syn} values in ten cells (fitted equation has the form I_{max} $(1 - \exp(-af^n))$, where I_{max} = 83 bits/s, a = 0.1, n = 1.2, and f is frequency).

(D) Energy use on pumping out of postsynaptic ion influx as a function of g_{syn} multiplier used in dynamic clamp.

(E) Information divided by energy used on reversing the ion influx generating postsynaptic currents as a function of g_{syn} in ten cells (with, for each cell, the efficiency being normalized to the value at $g_{syn} \times 1$; individual data for each cell are shown in Figure S3). The averaged data, shown in black, reveal a maximum very close to the physiological value of g_{syn}. 100% corresponds to 15.6 ± 2.7 bits per 10^8 ATP molecules used. Equation fitted to the mean data has the form $100.g_{syn}/g_{opt}$ for $g_{syn} < g_{opt}$ and $100.\exp(-(g_{syn} - g_{opt})/K)$ for $g_{syn} > g_{opt}$, where g_{opt} = 0.91 and K = 9.36.

(F) Information divided by energy use on reversing the ion influx generating postsynaptic currents and postsynaptic action potentials also shows a maximum near the physiological value of g_{syn}. Data are averaged over ten cells. 100% corresponds to 9.0 ± 1.2 bits per 10^8 ATP molecules used. Fitted equation is as in (E) but with g_{opt} = 0.78 and K = 9.95.

Data are represented as mean ± SEM. See also Figures S3–S5.

energy expended on postsynaptic ion influx. Strikingly, at the retinal ganglion cell to lateral geniculate nucleus synapse, an increase of postsynaptic conductance (implemented experimentally using dynamic clamp) can increase information flow through the synapse 4-fold (Figure 5A), demonstrating that the synapse properties are far from being optimized to maximize information transmission. Indeed, only about one in five presynaptic action potentials evokes a postsynaptic action potential. However, calculating the energy used on pumping out the Na^+ ions that enter through the postsynaptic conductance (Figure 5E) and during postsynaptic action potentials (Figure 5F) shows that the evolutionarily selected value of the conductance maximizes the ratio of the number of bits of information transmitted through the synapse to the ATP used on ion pumping.

Similar computational sacrifices in favor of energetic efficiency have been observed elsewhere in the brain. The mean firing rate of CNS neurons (~4 Hz) [6, 21] is much less than the rate that would maximize information coding capacity (half the maximal firing frequency or around 200 Hz for a refractory period of 2.5 ms) [7]. This has been explained [7] in terms of neurons maximizing the ratio of the amount of information they represent to the energy used on propagating the information as action potentials (which may itself be reduced by optimization of the properties of the active conductances generating action potentials) [22–24]. Similarly, the surprisingly low release probability of

central synapses has been explained [4] in terms of presynaptic terminals operating to maximize the information transmitted per energy used. The results presented here extend this concept to postsynaptic terminals, the largest consumers of energy in the brain. Together with previous results recognizing that energy use is a significant constraint on neuronal function [6, 7, 21, 25–27], our data suggest maximization of information transmission per energy used is an important functional principle in the brain.

These conclusions were obtained for the retinal ganglion cell to lateral geniculate nucleus cell excitatory synapse studied in isolation in brain slices, with cortical input removed and inhibition blocked pharmacologically. This approach was taken in order to analyze the relationship between information transfer and energy use for a single synapse. In vivo, the presence of cortical input and local inhibition might significantly alter the overall transmission of information through the LGN. However, the fraction of input information that is transmitted that we find is similar to that measured in in vivo experiments (see Results) and when experiments were carried out without GABA receptors blocked the results obtained were similar (see Supplemental Information).

Whether energy optimization governs the postsynaptic properties of all excitatory synapses is still unknown. Minimization of postsynaptic energy consumption is, however, likely to be an important constraint on the operation of the weak parallel fiber to Purkinje cell synapse in the cerebellum because, after motor learning, approximately 85% of these synapses are turned off [28], greatly reducing the energy consumption of the cerebellar cortex [5]. At the other end of the spectrum are synapses like the calyx of Held or neuromuscular junction, where one synaptic input is sufficient to drive a highly reliable postsynaptic response. At such synapses, it may seem that faithful transmission must be favored at the expense of energy efficiency. However, research at the calyx of Held has shown that, over development, vesicle exocytosis becomes more efficient and release probability decreases, reducing postsynaptic receptor saturation and desensitization [29, 30], suggesting that such synapses do not use more resources than are necessary to transmit high fidelity information. We think it likely that close examination of a variety of synapses will reveal a widespread principle of energy-efficient information transmission in the brain.

Optimization of the energetic efficiency of synapses may confer additional coding benefits. The fact that the postsynaptic conductance at the retina-LGN synapse is not large enough to guarantee transmission of every retinal action potential not only maximizes the information transmitted per ATP used, as shown above, it also results in a more-efficient transmission of action potentials that occur close together in time [12] (Figure 1E), presumably because postsynaptic summation is needed to reach the threshold for production of a postsynaptic action potential. This occurs despite a decrease of EPSC size occurring (Figure S1) for the second of two action potentials that are close together in time (which can be viewed as a type of gain control) and leads to a change of code at the retina-LGN synapses, from a code where action potential correlations carry most information to a code where each action potential encodes information independently [31]. It is important to realize, however, that this modulation of EPSC size by paired pulse depression is automatically taken account of in our information analysis.

If the ratio of information transmitted to energy consumed at synapses has to be optimized for normal brain function, this raises the question of whether neurological or psychiatric disorders may arise when this ratio is perturbed. Insertion of too few postsynaptic receptors will lead to an excessive loss of information, whereas inserting too many postsynaptic receptors could increase the local energetic demand beyond that that can be met by the ATP supply from local mitochondria. To understand how the brain avoids these problems, it will be necessary to identify the mechanisms by which neurons assess how well they are optimizing information flow in relation to energy consumption. Intriguingly, energy reduction techniques are being introduced to nanoelectronics in which relatively unimportant connections in a semiconductor chip are removed in order to save energy, at the cost of some degradation in the accuracy of the computation performed [32, 33]. This probabilistic pruning of the circuitry has an effect similar to the sacrifice of information transfer made by neurons that adopt a low postsynaptic conductance in order to save energy.

EXPERIMENTAL PROCEDURES

Visual Pathway Slice Preparation

P28 Sprague Dawley rats were killed by overdose of isoflurane anesthetic, in accordance with the guidelines of the UK Animals (Scientific Procedures) Act 1986 and subsequent amendments. The brain was rapidly removed and immersed in ice-cold, slicing solution containing (in mM) 87 NaCl, 25 $NaHCO_3$, 7 $MgCl_2$, 2.5 KCl, 1.25 NaH_2PO_4, 0.5 $CaCl_2$, 25 glucose, 75 sucrose, and 1 kynurenic acid, saturated with 95% O_2/5% CO_2 (modified from [34]).

Parasagittal brain slices containing the dorsal lateral geniculate nucleus (dLGN) were obtained as described [8]. Briefly, each hemisphere was isolated using a cut either side of the midline at 3°–5° to the sagittal plane, angled outward by 10°–25° in the mediolateral plane. The medial side of each brain half was glued to the cutting stage of a vibratome (Leica VT1200S) and submerged in ice-cold continuously oxygenated slicing solution, and 225-μm slices were made. In general, only a single slice from each hemisphere contained the optic tract and its fibers radiating to the dLGN. Before use, the cortex of each slice was removed using a scalpel to prevent disynaptic excitation via the thalamocortical feedback loop.

Slices were placed in a storage chamber containing continuously oxygenated slicing solution at 35°C, which was allowed to come to room temperature naturally. During the experiment, slices were continuously perfused with artificial cerebrospinal fluid (aCSF) containing (in mM) 124 NaCl, 26 $NaHCO_3$, 10 glucose, 2.5 KCl, 2 $CaCl_2$, 1 NaH_2PO_4, 1 $MgCl_2$, and 0.005 gabazine (to block disynaptic inhibition during stimulation). The aCSF was heated to 35°C and constantly bubbled with 95% O_2/5% CO_2.

Electrophysiology

Whole-cell recordings from LGN relay neurons were obtained using 2- to 3-MΩ borosilicate glass electrodes filled with internal solution containing (in mM) 130 K-gluconate, 10 EGTA, 10 HEPES, 4 NaCl, 4 MgATP, 1 $CaCl_2$, 0.5 Na_2GTP, and 0.4 K_2-Lucifer yellow. Relay neurons were identified by their large cell bodies (15–25 μm) and the presence of a hyperpolarization-activated inward current [35]. Throughout the experiment, relay neurons were held at −55 mV (by injection of a small amount of current, as resting potentials were typically around −70 mV), in order to restrict them to firing in tonic mode, where one input spike generally produces one output spike, as opposed to burst mode at <−70 mV, where one input spike generally leads to a burst of output spikes [36, 37].

Online corrections were made for the junction potential of −14 mV for the gluconate-based internal solution used (e.g., neurons were held at an apparent potential of −41 mV to achieve a true potential of −55 mV for LGN cells).

Recordings were made with an Axopatch 200B amplifier, filtered at 5 kHz, and sampled at 20 kHz. Data were acquired using custom-made MATLAB software, kindly provided by Ho Ko and Tom Mrsic-Flogel, UCL.

The first part of each experiment was performed in voltage clamp. Upon seal formation, pipette capacitance was compensated. Once in whole-cell mode, the series resistance was compensated by up to 70% (after which the mean series resistance was 6.7 ± 0.6 MΩ). The second part of the experiment was performed in current clamp, using the I-CLAMP FAST mode (which was stable with the 2- to 3-MΩ pipettes used). In current clamp mode, series resistance compensation was set to 100%.

Retinal ganglion cell (RGC) axons in the optic tract were stimulated extracellularly with a borosilicate glass electrode (gently broken to achieve a tip diameter of ~10–15 μm) containing aCSF. In voltage clamp, stimulation was adjusted to achieve the smallest reliable EPSC (defined as an EPSC that, when it occurred, did not vary in size in response to a pulse delivered every 3 s). The EPSC size usually increased in one clear step, and the stimulus intensity could therefore be set to activate a single presynaptic RGC axon [10, 11] (Figure S1A). This intensity was then maintained throughout the experiment. The average EPSC size (949 ± 141 pA) was similar to that found previously at this age [10, 11] (Figure S1B). All recordings used showed the strong paired pulse depression characteristic of this synapse [10, 11, 38] (PPR = 0.39 ± 0.05; Figures S1C and S1D).

Stimulation Pattern

RGC axons were stimulated (Figures 1A and S2A) with five 5-s spike trains (average frequency ~19 Hz), recorded [9] from ON-RGCs in isolated mouse retinae in response to five natural movies and kindly provided by Sheila Nirenberg, Cornell. We cannot be sure that the type of ganglion cell axon stimulated is exactly the same as that recorded in the Nirenberg experiments. Nevertheless, there are no publications (to our knowledge) suggesting that the output synapses of different classes of ganglion cell differ in their mechanisms. Determining the energy efficiencies of the synapses to LGN cells from different classes of ganglion cell will be an interesting area to study in future.

After an initial run of train 3 to habituate the synapse (EPSCs tended to be substantially larger directly after a period of no stimulation; Figure S1C), from which the data were discarded, the five trains were played in sequence (1-2-3-4-5), five times, resulting in a 125-s stimulation train from which data were collected. This procedure was followed once in voltage clamp, once in current clamp, and several times in dynamic clamp with various conductance gains (see below).

Dynamic Clamp

The 125-s postsynaptic current recording obtained from LGN relay neurons in voltage clamp was used to calculate a 125-s conductance train. First, the stimulation artifacts were removed by setting the current value for the duration of the artifact to the current value immediately preceding the artifact (Figures 2B, inset, and 2D, inset). The resulting current trace (I_{syn}) was converted to a conductance trace (g_{syn}) via

$$g_{syn}(t) = I_{syn}(t) / (V_m - V_{rev}),$$

where V_m is the membrane potential of the cell (the holding potential; −55 mV) and V_{rev} is the reversal potential of the synapse (0 mV; the reversal potential for glutamatergic ionotropic receptors). g_{syn} was then scaled up or down by a factor of 0.1, 0.3, 0.5, 0.75, 1, 1.5, 3, 6, 9, or 12. The new 125-s conductance trace (g_{syn}) was then applied directly to the postsynaptic cell using dynamic clamp [20] (SM-1; Cambridge Conductance), which injects a time-varying current $I_{inj}(t)$, at time t, calculated from $g_{syn}(t)$ and the instantaneous value of the cell membrane potential

$$I_{inj}(t) = g_{syn}(t) \times (V_m(t) - V_{rev}).$$

Because of the liquid junction potential, the V_m received by the SM-1 was 14 mV more positive than the real membrane potential. We therefore set V_{rev} on the SM-1 to 14 mV (rather than 0 mV) to account for this in the online calculation of I_{inj}. In this calculation, all of the synaptic current was assumed to scale linearly with membrane potential (i.e., non-linearities related to the magnesium block of NMDA receptors were not mimicked here, but detailed simulations showed that this had no qualitative effect on the relationship between synaptic conductance and the ratio of information transmitted to energy used; Figure S4).

The voltage response of the postsynaptic cell was simultaneously recorded. When the conductance was scaled by 1 and applied by dynamic clamp, the postsynaptic firing pattern was similar to that recorded when electrical stimulation was applied presynaptically (compare Figures 2C and 2E). Thus, although in dynamic clamp the conductance increases were applied at the soma rather than in the dendrites, this did not appear to affect the cell's decision to spike. This was probably because (1) the conductance injected at the soma was originally recorded at the soma was originally recorded at the soma, and was thus already "filtered" by the dendrites, and may mimic the current injected into the soma from the dendrites during synaptic simulation; and (2) relay neurons in the LGN are highly electrically compact [39], which, along with the close proximity of the retinogeniculate synapse to the cell body [40] (<100 μm), implies that the retinal input seen by the soma is only mildly attenuated compared to that seen by the dendrites.

To prevent possible damage to the cell with large injected conductances, the traces scaled by 6, 9, or 12 were always injected last. The order of injection of the smaller conductances was randomized.

Data Analysis

Data were analyzed using custom scripts written in MATLAB (The Mathworks). Postsynaptic current traces were used to calculate ATP consumption at the synapse as described below. Postsynaptic voltage traces were converted to binarized sequences of 1 s (representing action potentials) and 0 s (their absence) by identifying events whose amplitude exceeded a threshold defining action potential occurrences (set individually for each cell; between −15 mV and −30 mV). This output sequence could then be compared with the binary input spike train to look at simple transmission characteristics (Figure 1) or used to calculate the amount of information that would be propagated to visual cortex by the postsynaptic cell (Figures 3 and 5).

Synapse Transmission Characteristics

To assess how the probability of an output spike depends on presynaptic interstimulus interval (ISI) at the RGC-LGN synapse, output spikes were searched for in an 18-ms time window following the second spike of an ISI pair (18 ms was chosen because it encompasses the majority of the action potentials evoked by an input at all dynamic clamp gains; Figures S5A–S5D). If an output spike was present, the preceding ISI was counted as "relayed"; if not, the preceding ISI was counted as "non-relayed." The probability distribution for each category was calculated based on the total frequency counts for all 18 LGN relay neurons studied (Figure 1E). Note that, if a single presynaptic spike can sometimes evoke a postsynaptic action potential, this procedure has the potential to artifactually indicate action potential production by large ISIs when in fact it was only the second action potential of a presynaptic pair that produced the postsynaptic action potential; consequently, this procedure overestimates the frequency of action potential production by large ISIs.

To assess the occurrence of output responses produced by an input spike, the 18 ms following each input spike was searched for either a 1 in the binarized output trace (indicating a postsynaptic action potential) or an EPSP in the original voltage trace (with a minimum threshold of 1 mV). If neither of these were found, no output response was considered to have occurred. If two inputs arrived within 18 ms of each other, the search window following the first input was ended at the time of the second input. The probability of each outcome was calculated from its relative occurrence across all 18 cells in Figures 1F and 1G.

To assess the probability of an input spike given an output spike, the 18 ms preceding each output spike was searched for a 1 in the binarized input trace (indicating an input spike). If this was not found, the postsynaptic spike was considered to have occurred spontaneously. If two output spikes occurred within 18 ms of each other, the search window preceding the second output spike was ended at the time of the first output spike. The probability of each outcome was calculated from its relative occurrence across all 18 relay cells (Figures 1F and 1H).

The value chosen for the time window after a presynaptic spike or before a postsynaptic spike is not critical. Its main effect is on the pie charts in Figures 1G and 1H. Reducing the window to 9 ms or increasing it to 30 ms alters the percentage of occasions on which a presynaptic AP generates a postsynaptic

AP from 19% (Figure 1G) to 16% or 20%, respectively (as a longer time window results in more postsynaptic APs being found). More significantly, the same alterations of time window alter the percentage of output APs that are not associated with an input AP from 7% (Figure 1H) to 20% or 3%, respectively (again because a longer time window results in more presynaptic APs being found).

Calculating Synaptic Energy Use

For voltage-clamp conditions, the ATP used to reverse the postsynaptic ion flux (which is the largest synaptic energy cost) [4] was calculated from the postsynaptic current trace recorded in response to presynaptic stimulation. The current trace was integrated to obtain the total charge entry over the 125-s recording (Figure 4C). Dividing this by the charge on a Na^+ ion gives an estimate of the Na^+ influx. However, because K^+ efflux is occurring simultaneously (through the non-specific cation pores of AMPA and NMDA receptors), the actual Na^+ entry is 1.42 times larger than this (see next paragraph). The total Na^+ influx must then be actively pumped out by the Na^+/K^+-ATPase, which uses one ATP molecule per three Na^+ ions. This ATP cost was divided by the length of the recording (125 s) to get a rate of energy consumption (in ATP molecules/s) for each cell.

For voltage recording in current clamp mode using dynamic clamp, with the synaptic conductance scaled up or down, energy consumption was calculated differently. First, the synaptic Na^+ conductance (g_{Na}) was calculated from the total synaptic conductance (g_{syn}) by assuming that the contributions to the total AMPA receptor current carried by Na^+ and K^+ (Ca^{2+} was neglected) vary ohmically with voltage displacement from the reversal potentials V_{Na} (+90 mV) and V_K (−105 mV), so that (for a synaptic current reversal potential of V_{rev} = 0 mV)

$$g_{Na} = g_{syn}/(1 - (V_{Na}/V_K)).$$

For the experimentally imposed reversal potentials stated above, g_{Na} = (7/13).g_{syn}. The Na^+ current (I_{Na}) was then calculated directly from the Na^+ conductance, the Na^+ reversal potential, and the membrane potential of the cell as

$$I_{Na}(t) = g_{Na}(t) \times (V_m(t) - V_{Na}).$$

The integral of $I_{Na}(t)$ was then used to calculate the total postsynaptic Na^+ entry over the 125-s recording (Figure 4E), which was converted to a rate of energy consumption as above. For voltage-clamp experiments at our holding potential of −55 mV, the Na^+ entry calculated from the equation above can be shown (using the relationship between g_{Na} and g_{syn} given above) to be 1.42-fold larger than the charge entry measured from the synaptic current as $g_{syn}(t) \times (V_m(t) - V_{rev})$.

Calculating Action Potential Energy Use

Action potentials in rodent thalamocortical relay neurons have been found to be highly energy efficient, costing 1.35×10^{11} ATP molecules/AP/cm^2 of membrane [24]. From the recorded membrane capacitance for each LGN cell (mean = 152 ± 8 pF) and the standard biological membrane capacitance (1 $\mu F/cm^2$), we could calculate the surface area, and thus the action potential cost, for each cell (mean = $2.05 \pm 0.11 \times 10^7$ ATP molecules/AP/cell). This value—calculated for each LGN cell—was multiplied by the firing frequency of the cell in each stimulation condition to obtain the energy used on action potentials per second.

Calculating Information

To calculate the information transmitted across a synapse, we employed information theory [41] to estimate the mutual information between the input and output spike trains. The calculations are described in detail in the Supplemental Information. The so-called direct method [17, 19] requires an input train made up of unique and repeating sections, responses to which are used to calculate the total entropy and the noise entropy in the output signal, respectively. Mutual information is the total entropy minus the noise entropy. A major advantage of this method is that it does not require any assumptions about correlations between spikes or the temporal relationship between input and output spikes. An alternative method gave similar results (see Supplemental Experimental Procedures and Figure S5D).

Analysis of Energetic Efficiency

For each condition (real stimulation and all dynamic clamp gains), the information rate was divided by the rate of energy consumption on reversing the ion flux generating EPSCs (Figure 5E), or on reversing the ion flux generating EPSCs and postsynaptic action potentials (Figure 5F), to get a measure of efficiency in bits/(ATP consumed).

Statistics

Data are presented as mean ± SEM. Differences between means were assessed with Student's t tests and corrected for multiple comparisons using a modified Holm-Bonferroni method; differences are taken as significant when $p < 0.05$.

SUPPLEMENTAL INFORMATION

Supplemental Information includes five figures and Supplemental Experimental Procedures and can be found with this article online at http://dx.doi.org/10.1016/j.cub.2015.10.063.

AUTHOR CONTRIBUTIONS

J.J.H., R.J., and D.A. conceived experiments. J.J.H., R.J., and E.E. performed experiments. R.J., J.J.H., and E.E. performed analyses. J.J.H., R.J., E.E., and D.A. wrote and reviewed the manuscript. D.A. supervised and acquired funding.

ACKNOWLEDGMENTS

We thank Sheila Nirenberg for ganglion cell responses to natural visual scenes; Ho Ko for software; Mickey London, Hugh Robinson, Arnd Roth, and Tom Salt for advice; and Guy Billings, Tiago Branco, Matteo Carandini, Lee Cossell, Peter Dayan, Alasdair Gibb, Sonja Hofer, Simon Laughlin, Nick Lesica, Zhaoping Li, Tom Mrsic-Flogel, and Angus Silver for comments. This work was supported by the European Research Council, Fondation Leducq, the Medical Research Council, the Wellcome Trust, and a Marie Curie Fellowship of the EU to R.J. J.J.H. and E.E. were in UCL's 4-year PhD Programme in Neuroscience.

Received: September 8, 2015
Revised: October 22, 2015
Accepted: October 27, 2015
Published: December 3, 2015

REFERENCES

1. Attwell, D., and Gibb, A. (2005). Neuroenergetics and the kinetic design of excitatory synapses. Nat. Rev. Neurosci. *6*, 841–849.
2. Hestrin, S. (1992). Activation and desensitization of glutamate-activated channels mediating fast excitatory synaptic currents in the visual cortex. Neuron *9*, 991–999.
3. Traynelis, S.F., Silver, R.A., and Cull-Candy, S.G. (1993). Estimated conductance of glutamate receptor channels activated during EPSCs at the cerebellar mossy fiber-granule cell synapse. Neuron *11*, 279–289.
4. Harris, J.J., Jolivet, R., and Attwell, D. (2012). Synaptic energy use and supply. Neuron *75*, 762–777.
5. Howarth, C., Gleeson, P., and Attwell, D. (2012). Updated energy budgets for neural computation in the neocortex and cerebellum. J. Cereb. Blood Flow Metab. *32*, 1222–1232.
6. Attwell, D., and Laughlin, S.B. (2001). An energy budget for signaling in the grey matter of the brain. J. Cereb. Blood Flow Metab. *21*, 1133–1145.
7. Levy, W.B., and Baxter, R.A. (1996). Energy efficient neural codes. Neural Comput. *8*, 531–543.
8. Turner, J.P., and Salt, T.E. (1998). Characterization of sensory and corticothalamic excitatory inputs to rat thalamocortical neurones in vitro. J. Physiol. *510*, 829–843.

9. Meytlis, M., Nichols, Z., and Nirenberg, S. (2012). Determining the role of correlated firing in large populations of neurons using white noise and natural scene stimuli. Vision Res. *70*, 44–53.
10. Budisantoso, T., Matsui, K., Kamasawa, N., Fukazawa, Y., and Shigemoto, R. (2012). Mechanisms underlying signal filtering at a multisynapse contact. J. Neurosci. *32*, 2357–2376.
11. Chen, C., and Regehr, W.G. (2000). Developmental remodeling of the retinogeniculate synapse. Neuron *28*, 955–966.
12. Mastronarde, D.N. (1987). Two classes of single-input X-cells in cat lateral geniculate nucleus. II. Retinal inputs and the generation of receptive-field properties. J. Neurophysiol. *57*, 381–413.
13. Rathbun, D.L., Warland, D.K., and Usrey, W.M. (2010). Spike timing and information transmission at retinogeniculate synapses. J. Neurosci. *30*, 13558–13566.
14. Sincich, L.C., Horton, J.C., and Sharpee, T.O. (2009). Preserving information in neural transmission. J. Neurosci. *29*, 6207–6216.
15. Usrey, W.M., Reppas, J.B., and Reid, R.C. (1999). Specificity and strength of retinogeniculate connections. J. Neurophysiol. *82*, 3527–3540.
16. Weyand, T.G. (2007). Retinogeniculate transmission in wakefulness. J. Neurophysiol. *98*, 769–785.
17. Strong, S.P., Koberle, R., de Ruyter van Steveninck, R.R., and Bialek, W. (1998). Entropy and information in neural spike trains. Phys. Rev. Lett. *80*, 197–200.
18. Koch, K., McLean, J., Segev, R., Freed, M.A., Berry, M.J., 2nd, Balasubramanian, V., and Sterling, P. (2006). How much the eye tells the brain. Curr. Biol. *16*, 1428–1434.
19. Reinagel, P., and Reid, R.C. (2000). Temporal coding of visual information in the thalamus. J. Neurosci. *20*, 5392–5400.
20. Robinson, H.P., and Kawai, N. (1993). Injection of digitally synthesized synaptic conductance transients to measure the integrative properties of neurons. J. Neurosci. Methods *49*, 157–165.
21. Perge, J.A., Koch, K., Miller, R., Sterling, P., and Balasubramanian, V. (2009). How the optic nerve allocates space, energy capacity, and information. J. Neurosci. *29*, 7917–7928.
22. Alle, H., Roth, A., and Geiger, J.R. (2009). Energy-efficient action potentials in hippocampal mossy fibers. Science *325*, 1405–1408.
23. Carter, B.C., and Bean, B.P. (2009). Sodium entry during action potentials of mammalian neurons: incomplete inactivation and reduced metabolic efficiency in fast-spiking neurons. Neuron *64*, 898–909.
24. Sengupta, B., Stemmler, M., Laughlin, S.B., and Niven, J.E. (2010). Action potential energy efficiency varies among neuron types in vertebrates and invertebrates. PLoS Comput. Biol. *6*, e1000840.
25. Laughlin, S.B., and Sejnowski, T.J. (2003). Communication in neuronal networks. Science *301*, 1870–1874.
26. Perge, J.A., Niven, J.E., Mugnaini, E., Balasubramanian, V., and Sterling, P. (2012). Why do axons differ in caliber? J. Neurosci. *32*, 626–638.
27. Niven, J.E., and Farris, S.M. (2012). Miniaturization of nervous systems and neurons. Curr. Biol. *22*, R323–R329.
28. Isope, P., and Barbour, B. (2002). Properties of unitary granule cell→Purkinje cell synapses in adult rat cerebellar slices. J. Neurosci. *22*, 9668–9678.
29. Taschenberger, H., Leão, R.M., Rowland, K.C., Spirou, G.A., and von Gersdorff, H. (2002). Optimizing synaptic architecture and efficiency for high-frequency transmission. Neuron *36*, 1127–1143.
30. Fedchyshyn, M.J., and Wang, L.Y. (2005). Developmental transformation of the release modality at the calyx of Held synapse. J. Neurosci. *25*, 4131–4140.
31. Wang, X., Hirsch, J.A., and Sommer, F.T. (2010). Recoding of sensory information across the retinothalamic synapse. J. Neurosci. *30*, 13567–13577.
32. Palem, K., and Lingamneni, A. (2012). What to do about the end of Moore's law, probably! In Proceedings of the 49th Annual Design Automation Conference (ACM Digital Library), pp. 924–929.
33. Lingamneni, A., Muntimadugu, K.K., Enz, C., Karp, R.M., Palem, K.V., and Piguet, C. (2012). Algorithmic methodologies for ultra-efficient inexact architectures for sustaining technology scaling. In Proceedings of the 9th ACM International Conference on Computing Frontiers (ACM Digital Library), pp. 3–12.
34. Bischofberger, J., Engel, D., Li, L., Geiger, J.R., and Jonas, P. (2006). Patch-clamp recording from mossy fiber terminals in hippocampal slices. Nat. Protoc. *1*, 2075–2081.
35. McCormick, D.A., and Pape, H.C. (1990). Properties of a hyperpolarization-activated cation current and its role in rhythmic oscillation in thalamic relay neurones. J. Physiol. *431*, 291–318.
36. Scharfman, H.E., Lu, S.M., Guido, W., Adams, P.R., and Sherman, S.M. (1990). N-methyl-D-aspartate receptors contribute to excitatory postsynaptic potentials of cat lateral geniculate neurons recorded in thalamic slices. Proc. Natl. Acad. Sci. USA *87*, 4548–4552.
37. Reinagel, P., Godwin, D., Sherman, S.M., and Koch, C. (1999). Encoding of visual information by LGN bursts. J. Neurophysiol. *81*, 2558–2569.
38. Chen, C., Blitz, D.M., and Regehr, W.G. (2002). Contributions of receptor desensitization and saturation to plasticity at the retinogeniculate synapse. Neuron *33*, 779–788.
39. Bloomfield, S.A., and Sherman, S.M. (1989). Dendritic current flow in relay cells and interneurons of the cat's lateral geniculate nucleus. Proc. Natl. Acad. Sci. USA *86*, 3911–3914.
40. Wilson, J.R., Friedlander, M.J., and Sherman, S.M. (1984). Fine structural morphology of identified X- and Y-cells in the cat's lateral geniculate nucleus. Proc. R. Soc. Lond. B Biol. Sci. *221*, 411–436.
41. Shannon, C.E. (1948). A mathematical theory of communication. Bell Syst. Tech. J. *27*, 379–423.

Current Biology
Supplemental Information

Energy-Efficient Information Transfer by Visual Pathway Synapses

Julia J. Harris, Renaud Jolivet, Elisabeth Engl, and David Attwell

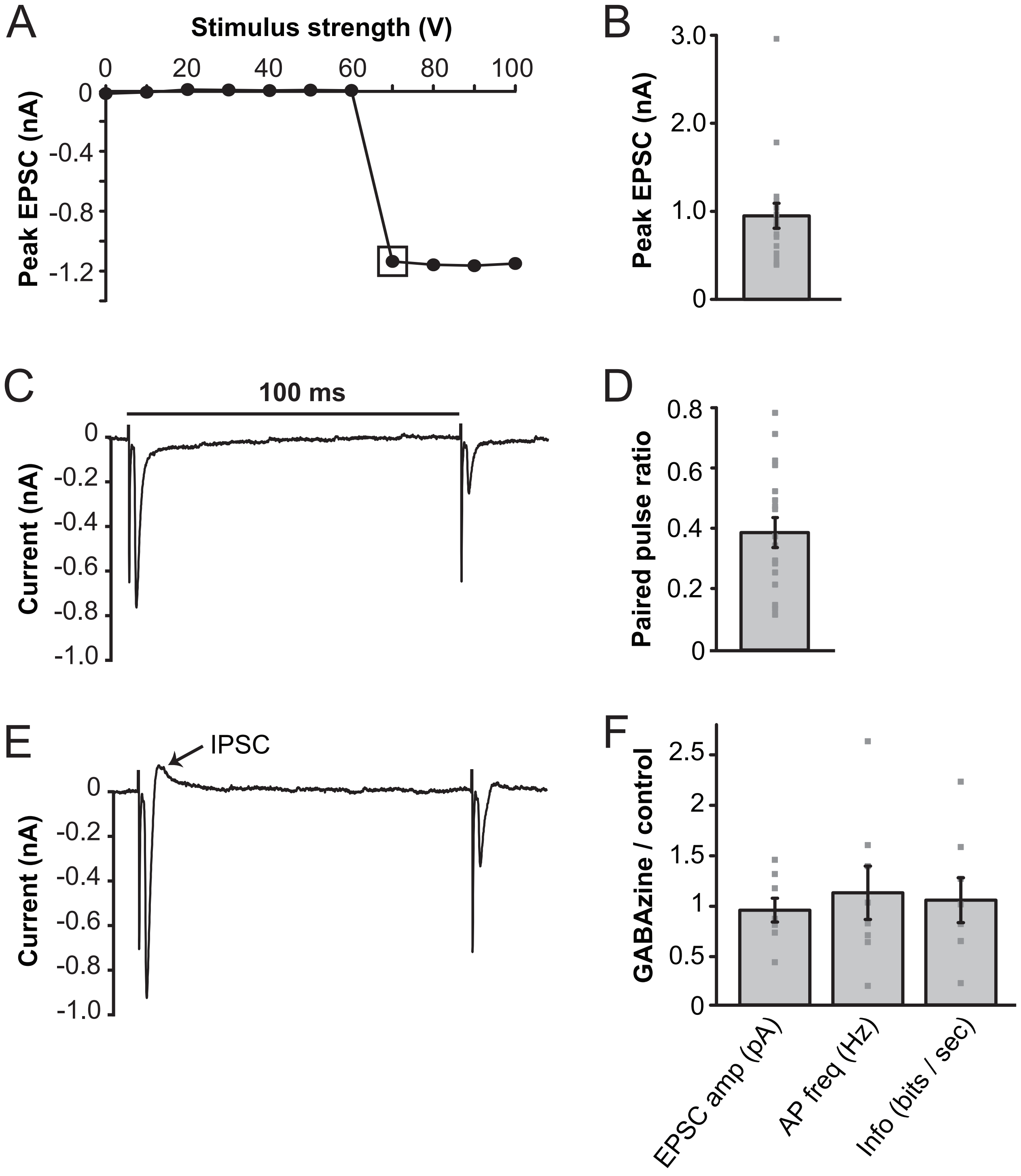
A
Stimulus strength (V)
0
20
40
60
80
100
Peak EPSC (nA)
0
-0.4
-0.8
-1.2
B
3.0
2.0
1.0
0
Peak EPSC (nA)
C
100 ms
0
-0.2
-0.4
-0.6
-0.8
-1.0
Current (nA)
D
0.8
0.6
0.4
0.2
0
Paired pulse ratio
E
IPSC
0
-0.2
-0.4
-0.6
-0.8
-1.0
Current (nA)
F
2.5
2
1.5
1
0.5
0
GABAzine / control
EPSC amp (pA)
AP freq (Hz)
Info (bits / sec)

Figure S1

Figure S1 (related to Figure 1). Characterization of the optic tract input to dLGN cells. (A) Stimulus-response curve shows a single increase indicating stimulation of one axon. (B) Mean amplitude (bar and s.e.m.) of first excitatory postsynaptic current (EPSC) in 18 cells (points). (C) Paired pulse depression of EPSC in a specimen cell evoked by stimuli 100 msec apart. (D) Mean (bar and s.e.m.) paired pulse ratio (2nd / 1st EPSC) for 100 msec separation in 18 cells (points). (E) In the absence of GABAzine, some cells display disynaptic inhibition (seen as an IPSC after the EPSC), which tends to show postsynaptic depression (mean paired pulse ratio of IPSC = 0.6±0.2). (F) For the cells with disynaptic inhibition, GABAzine does not significantly alter the EPSC size ($p = 0.62$), the action potential frequency ($p = 0.69$) or the information rate ($p = 0.87$) of presynaptically stimulated LGN neurons (8 cells) (bars with s.e.m. are mean data; individual cells shown as points).

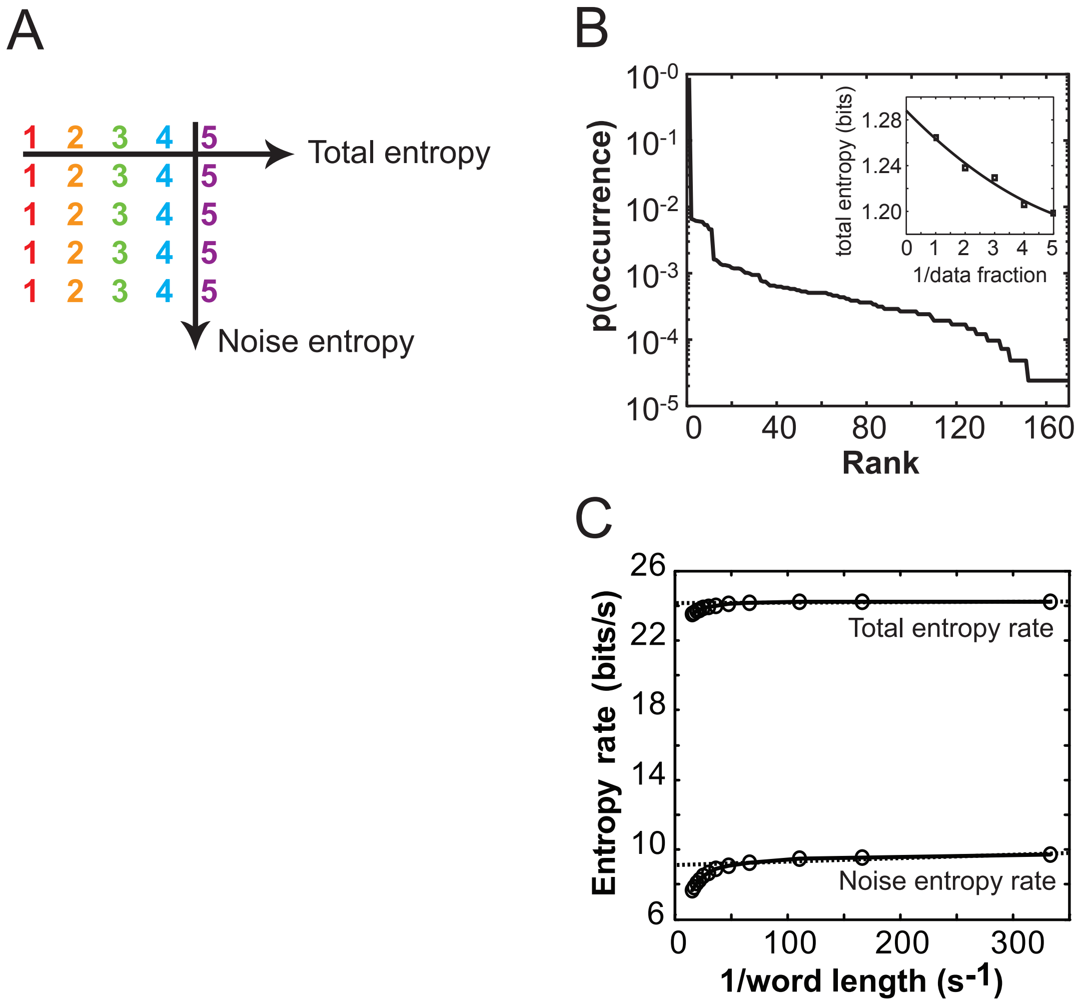
A
1 2 3 4 5
Total entropy
Noise entropy
B
p(occurrence)
Rank
total entropy (bits)
1/data fraction
C
Entropy rate (bits/s)
Total entropy rate
Noise entropy rate
1/word length (s-1)

Figure S2

Figure S2 (related to Figure 3). Calculating mutual information at the RGC-LGN synapse using the direct method. (A) Trains 1-5 were applied 5 times (in the order left to right, and then down the successive rows). Analysis of entropy across all 5 trains gives a measure of total response variability. Analysis of entropy across repeated application of the same train gives the noise entropy. (B) Probability of occurrence of each of the 10-letter words produced by application of all 5 trains for a specimen cell, ranked by probability value. Inset shows the dependence of the entropy, computed from this probability distribution according to the Shannon formula [3] (see Supplemental Experimental Procedures), on the fraction of data included in the analysis. Also plotted is a least squares fit of a quadratic function (see Supplemental Experimental Procedures), where the intercept is our extrapolation to the true value of the entropy with infinite data [1]. (C) Total and noise entropy for a specimen cell as a function of 1/word length (a word length of ten 3 msec bins corresponds to 1/word length = 33 sec^{-1}). Dashed lines indicate linear extrapolations to infinite word length [1].

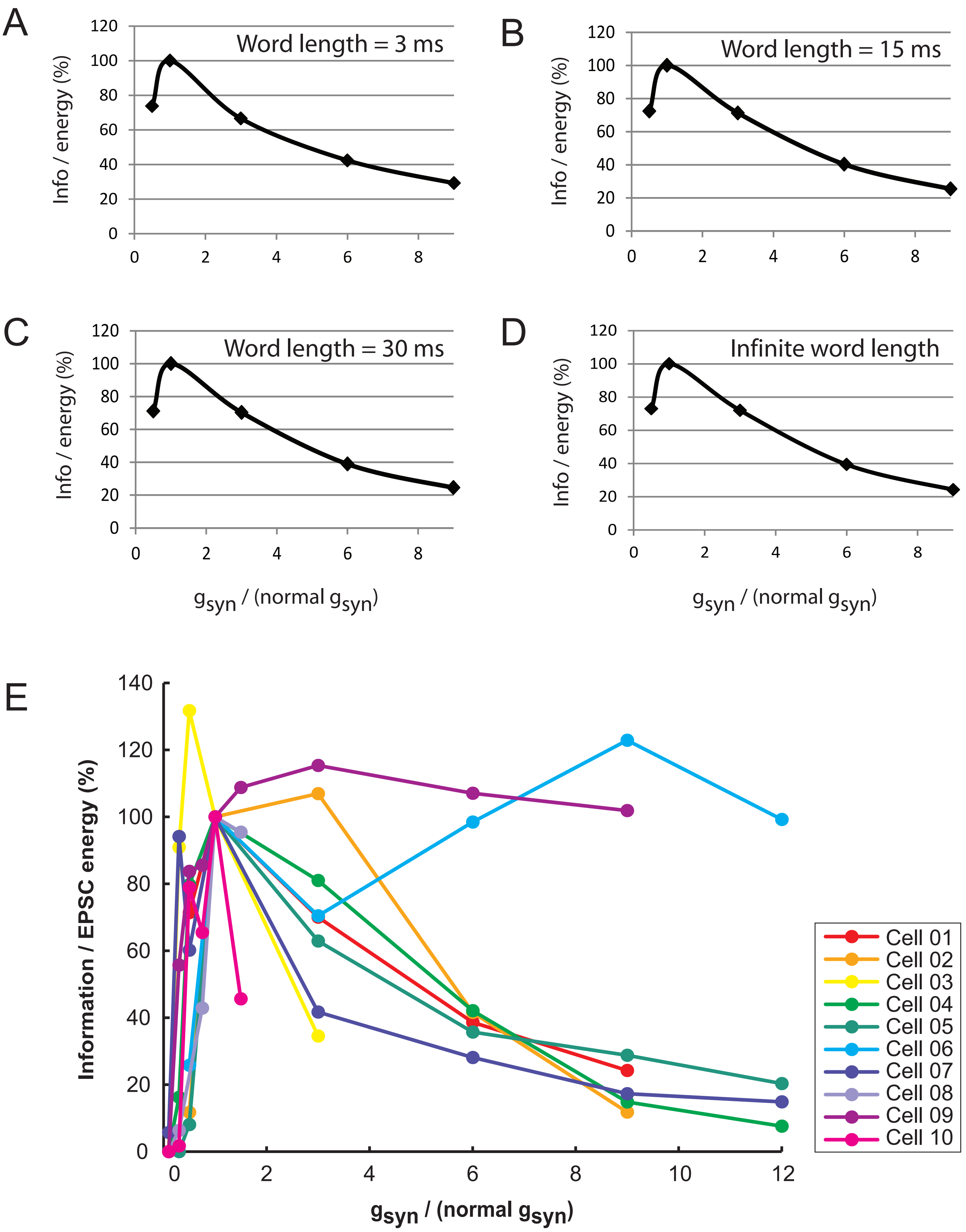

A
Word length = 3 ms
B
Word length = 15 ms
C
Word length = 30 ms
D
Infinite word length
Info / energy (%)
g_syn / (normal g_syn)
E
Information / EPSC energy (%)
g_syn / (normal g_syn)
Cell 01
Cell 02
Cell 03
Cell 04
Cell 05
Cell 06
Cell 07
Cell 08
Cell 09
Cell 10

Figure S3

Figure S3 (related to Figure 5). Information divided by energy used on reversing the ion influx generating postsynaptic currents as a function of g_{syn}. (A-D) Efficiency (bits transmitted / ATP used on EPSCs) plotted against synaptic conductance (altered using dynamic clamp to be 0.5, 1, 3, 6 and 9 times the biologically-occurring conductance) for an example cell. The relationship does not change when the information is calculated using words of length (A) 3 msec; (B) 15 msec; (C), 30 msec (used for all cells in Figure 5); or (D) linear extrapolation to infinite word length (as in Figure S2C). Bin size was always 3 msec. This analysis was performed on three cells, with similar results. (E) Information divided by energy used on reversing the ion influx generating postsynaptic currents in 10 individual cells plotted in different colours against g_{syn}. For each cell, the efficiency is normalised to the value at g_{syn}x1. Six out of ten cells show a maximum efficiency at the biological conductance value (g_{syn}x1), three cells show a maximum at a higher conductance magnitude (two at g_{syn}x3 and one at g_{syn}x9), and one cell shows a maximum at a lower conductance magnitude (at g_{syn}x0.5). The averaged data are shown in Figure 5E. 100% corresponds to 15.6±2.7 bits per 10^8 ATP molecules used.

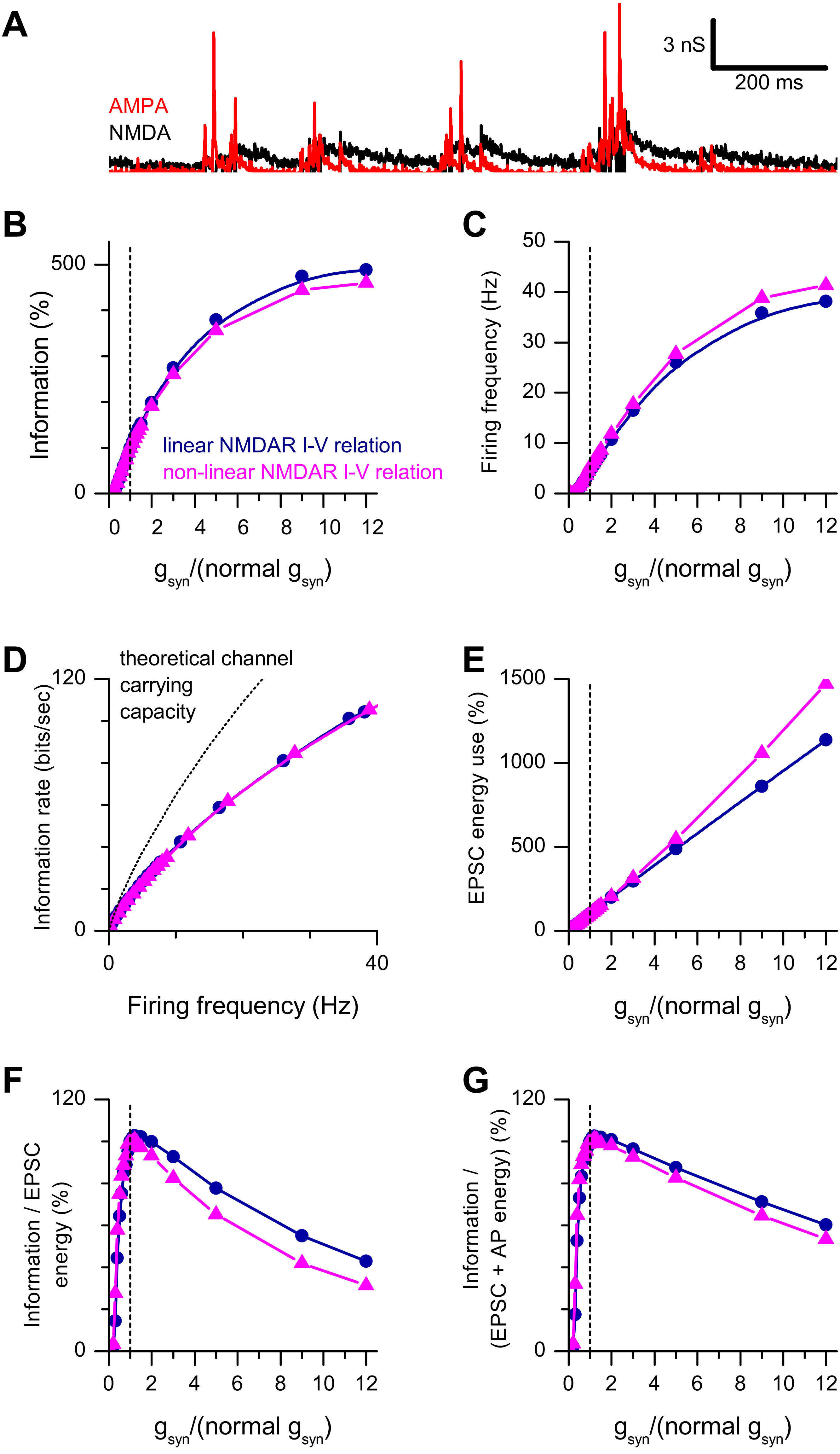
A
AMPA
NMDA
3 nS
200 ms
B
Information (%)
500
0
linear NMDAR I-V relation
non-linear NMDAR I-V relation
0 2 4 6 8 10 12
g_syn/(normal g_syn)
C
Firing frequency (Hz)
50 40 30 20 10 0
0 2 4 6 8 10 12
g_syn/(normal g_syn)
D
theoretical channel
carrying
capacity
Information rate (bits/sec)
120
0
0 40
Firing frequency (Hz)
E
EPSC energy use (%)
1500 1000 500 0
0 2 4 6 8 10 12
g_syn/(normal g_syn)
F
Information / EPSC energy (%)
120
0
0 2 4 6 8 10 12
g_syn/(normal g_syn)
G
Information / (EPSC + AP energy) (%)
120
0
0 2 4 6 8 10 12
g_syn/(normal g_syn)

Figure S4

Figure S4 (related to Figure 5 and Supplemental Experimental Procedures). Postsynaptic conductance magnitude maximising information transferred per energy used in a model of thalamic relay cells. (A) Specimen segments of the AMPA and NMDA conductance time course derived from experiments (see Supplemental Experimental Procedures) and used in the simulations. (B) Dependence of the output information on synaptic conductance (g_{syn}) magnitude, when the thalamic relay cell model (see Supplemental Experimental Procedures) was stimulated with the AMPA and NMDA conductance recorded experimentally in response to optic tract stimulation. The graph shows the effect of assuming a linear NMDAR I-V relation (blue) or a non-linear NMDAR I-V relation (violet; see Supplemental Experimental procedures). Information is normalized to the value with g_{syn} x1 for which the mean information rate was 21.4 bits/sec (linearized NMDA conductance) or 23.7 bits/sec (non-linear NMDA conductance). For comparison, the experimental information rate for recorded cells with presynaptic stimulation was 18.3±4.5 bits/sec. Colours are the same in B to F. (C) Relationship between firing frequency and g_{syn} in the simulations. (D) Dependence of output information on mean output firing frequency evoked by stimulus trains with different g_{syn} values. Linearizing the NMDAR I-V relation had no significant effect on this relation. The theoretical maximum channel capacity, given by:

$$-[f.\Delta t.\log_2(f.\Delta t) + (1-f.\Delta t).\log_2(1-f.\Delta t)]/\Delta t,$$

where f.Δt is the probability of an action potential occurring in the temporal bin width Δt (3 msec) at a firing frequency f, is also shown. (E) Energy use on pumping out of postsynaptic ion influx as a function of g_{syn} multiplier. (F) Output information divided by energy use on reversing the ion influx generating postsynaptic currents in simulations shows a maximum close to the physiological value of g_{syn} (102.8% at g_{syn}=1.2 with the NMDAR I-V relation linearized and 100.8% at g_{syn}=1.2 with a non-linear NMDAR I-V relation). 100% (at g_{syn} = 1) corresponds to 31.9 bits per 10^8 ATP molecules used with the NMDAR I-V relation linearized and to 32.3 bits per 10^8 ATP molecules used with the non-linear NMDAR I-V relation, slightly larger than the experimental value of 15.6±2.7 bits per 10^8 ATP molecules used. (G) Output information divided by energy use on reversing the ion influx generating postsynaptic currents and postsynaptic action potentials also shows a maximum close to the physiological value of g_{syn} (102.5% at g_{syn}=1.2 with the NMDAR I-V relation linearized and 101.1% at g_{syn}=1.2 with a non-linear NMDAR I-V relation). 100% (at g_{syn} = 1) corresponds to 8.0 bits per 10^8 ATP molecules used with the NMDAR I-V relation linearized and to 8.1 bits per 10^8 ATP molecules used with the non-linear NMDAR I-V relation, slightly below the experimental value of 9.0±1.2 bits per 10^8 ATP molecules used.

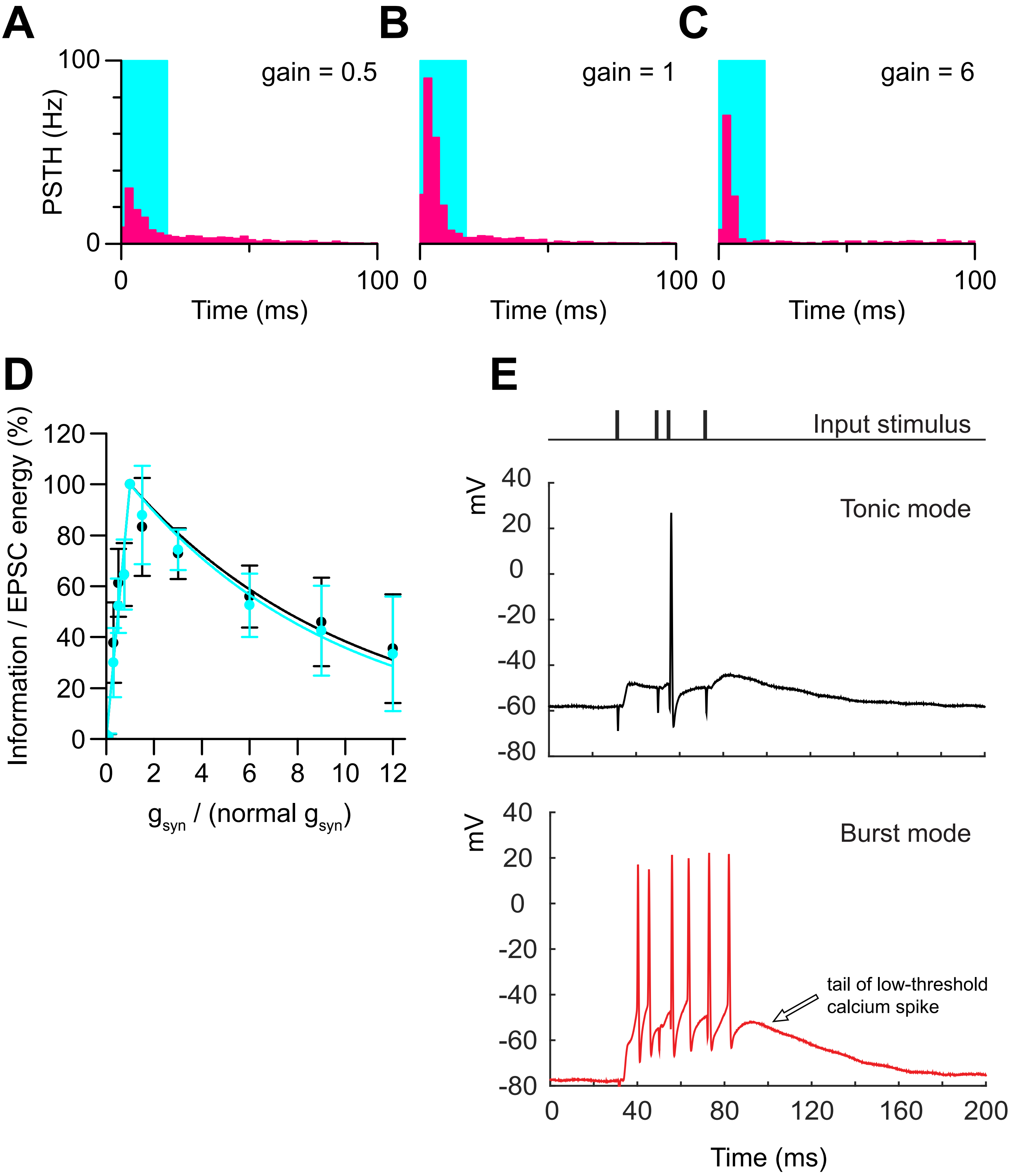
A
B
C
PSTH (Hz)
100
0
gain = 0.5
gain = 1
gain = 6
Time (ms)
D
Information / EPSC energy (%)
120
100
80
60
40
20
0
2
4
6
8
10
12
g_syn / (normal g_syn)
E
Input stimulus
mV
40
20
0
-20
-40
-60
-80
Tonic mode
Burst mode
tail of low-threshold
calcium spike
40
80
120
160
200
Time (ms)

Figure S5

Figure S5 (related to Figure 5 and Supplemental Experimental Procedures). Sampling window for assessing action potential transmission, and comparison of energy efficiency as a function of g_{syn} when information is estimated with two different methods. (A-C) Peristimulus time histograms for one example cell at three different scaling values for g_{syn}/(normal g_{syn}) (0.5, 1 and 6) illustrating that an 18 ms search window (cyan rectangle) contains most of the PSTH at gains and mutual information rates covering almost the entire range of the LGN data. (D) Comparison of energetic efficiency at the retinothalamic synapse obtained using mutual information estimates obtained with the direct method (black; same data as in Figure 5E) and using the alternate calculation described in the Supplemental Experimental Procedures with a search window of 18 msec (cyan). A similar peak in the relationship at g_{syn}/(normal g_{syn}) = 1 was seen even using search windows as small as 9 ms or as large as 30 ms. Data are represented as mean ±s.e.m. (E) Spiking response to the same input stimulus when an example cell was held in tonic mode (at approximately -55 mV) and burst mode (at approximately -75 mV). In burst mode, a small depolarization can trigger a low-threshold calcium spike, upon which multiple action potentials “ride”. This often means that one input spike triggers multiple output spikes, raising the question of whether one takes a single spike or a burst of spikes as the “output” in this mode of firing.

SUPPLEMENTAL EXPERIMENTAL PROCEDURES

Information analysis: direct method applied to retinogeniculate data

To calculate the information transmitted across the retinogeniculate synapse, we assessed the possible repertoire of signal encoding achievable by the optic tract to LGN neuron synapse by applying five separate 5 sec input trains evoked by different natural scenes (Figure 3A). For each recording, we applied these five trains in sequence (1-2-3-4-5), five times (Figure S2A), thus generating a 125 sec output spike train for analysis. We assessed the variability of the output response to the input information (to obtain the "total entropy", calculated below, Figure S2B), and also the variability in output response to a single input train applied on five separate occasions (to give the "noise entropy", calculated below).

To calculate the stimulus-related information in the output spike train, we used the direct method [S1]. Binarised output spike trains were binned with 3 msec precision (approximately the action potential refractory period). The binned trains were examined in segments of a fixed length, called "words" (a particular sequence of 1s and 0s), each word representing a possible neural response. For example, for a word length of five (3 msec) bins, an example word would be 10010. Words were allowed to overlap, so that each time bin was the start of a new word [S1 and S2]. The action potential string 100100001… would thus yield the 5 letter words 10010, 00100, 01000, 10000, 00001, etc. This maximised the number of words available for analysis in the 125 sec output spike train, the effects of correlations in which are removed by extrapolation to infinite word length [S1 and S2] as in Figure S2C. Words that are too long can cause a sampling bias (due to insufficient data being acquired) in which case the entropy (calculated below) is underestimated [S1 and S2]. Choice of word length for the analysis to avoid this problem is discussed below.

A probability distribution of word occurrences was built (Figure S2B), and used to calculate, for two distinct situations (described below), the Shannon entropy [S3], H (in bits), using the general formula:

$$H = -\Sigma\ p(\text{word}) \times \log_2 [p(\text{word})]$$

where p is the probability of a particular word occurring.

First, a probability distribution was built for the occurrence of all possible words of a given length, by counting the frequency of occurrence of each word in the first 25 sec of stimulation (i.e. across the first run of all five trains, represented by the horizontal arrow in Figure S2A). This probability distribution (Figure S2B) was used to calculate the total entropy (H_{total}) of the first 25 sec of the spike train (using the formula above). H_{total} was calculated in this way for each successive run of the five different input trains (i.e. for each row in Figure S2A), and then averaged to get a final estimate of H_{total} for the whole 125 sec recording. H_{total} reflects the maximum possible variability of the postsynaptic response for the input trains used, and a higher value reflects a higher information capacity in the spike train.

Second, to assess variability in the response to a repeated input train [S1], a probability distribution was built for the occurrence of all possible words (of a given length) that were evoked at a fixed time by a single input train when it was repeated five times. To do this, for a given word length, and looking at the responses over the 5 repeated identical trains, we counted the frequency of occurrence of words starting at a set time, *t*, after the onset of each repeated train. H_{noise} was calculated in the window from *t* to *t*+(word length) in the repeated train (using the formula above), and then averaged across all time points. H_{noise} was calculated in this way for each different repeated train (i.e. for each column in Figure S2A) and then averaged to get a final estimate of H_{noise} for the whole recording. H_{noise} reflects the trial-to-trial variability of the response, and a lower value reflects low noise levels in the system (i.e. little variability in the response to repeats of the same input).

Subtracting H_{noise} from H_{total} gives an estimate of the mutual information (I, in bits) between the output and input spike trains [S1], in other words, how informative the output spike train is about the input spike train:

$$I = H_{total} - H_{noise}$$

Because extrapolating to infinite word length [S1] as in Figure S2C was very computationally time-consuming, we defined the maximum word length that could be used before sampling problems arose by varying the word length between 3 and 63 ms (Figure S2C). We found that total entropy estimates based on 30 msec words differed by only 1.2±0.4% (averaged across dynamic clamp conditions for 3 cells) from the entropy estimate based on a linear extrapolation to infinite word length [S1 and S2], while the resulting value of information rate differed by only 0.3±0.6% from the value obtained by extrapolation. Similarly, the relationship between efficiency and g_{syn} was not affected by the use of either extrapolated entropy estimates or entropy estimates based on 30 msec words (or, indeed, words shorter than 30 msec; 3 cells, Figures S3A-S3D). 30 msec words were therefore used for the entropy calculations that follow. Throughout the paper, entropy and information estimates derived from this method are divided by the word length (30 msec) to get entropy and information rates in bits/sec.

Data adequacy for entropy calculations

Entropy estimates based on insufficient data could yield misleading results. However, there are significant experimental constraints on the length of whole-cell recordings (applying synaptic stimulation in voltage and current clamp modes, and then applying up to eight different conductance amplitudes in dynamic clamp takes around one hour and requires exceptional stability of the recorded cell). We therefore used each stimulus train (1-5, Figure 3A and Figure S2A) as both a “unique” input (to calculate total entropy) and a “repeat” input (to calculate noise entropy), thus maximising the utility of the data we collect. We checked whether this amount of data was sufficient to produce unbiased entropy estimates by applying the quadratic extrapolation correction described in ref. [S1] (their Figure 2 inset). Specifically, for every cell’s response to presynaptic stimulation, and for one cell’s response to every dynamic clamp condition, we fit a quadratic polynomial [S1] describing how

the entropy estimate converges when using increasing fractions of the dataset (Figure S2B inset). The correction (extrapolated value minus value obtained using the whole data set) averaged over the 10 cells for synaptic stimulation was only 1.3 ± 0.4% for the total entropy and 0.6 ± 0.3% for the noise entropy, and averaged over the 7 g_{syn} values in one specimen cell was only 1.0 ± 0.2% for the total entropy and 0.4 ± 0.4% for the noise entropy. We therefore used the empirical values (based on the entire dataset) rather than the quadratically extrapolated values for both total and noise entropies.

For the noise entropy, using 5 repetitions of the same input undersamples the distribution of words for word lengths of 3 bins (9 msec) or longer. Nevertheless, the relationship between synaptic energetic efficiency and g_{syn} was independent of the word length used for the analysis (Figure S3), and was replicated by a different method of calculating mutual information which avoids this undersampling problem (Figure S5D). For this second method, we segmented the 125 second input train into a series of 3 ms time bins (approximately the refractory period of a neuron) and calculated the information contained in this train (I_{input}) as:

$$I_{input} = -\, s.\log_2(s) - (1 - s).\log_2(1 - s)$$

where s is the probability of an action potential arriving in any given time bin, and I_{input} is given in bits per time bin [S2, S3 and S4]. We then calculated the mutual information per time bin (I_m), i.e. how much the sequence of output spikes measured in the LGN cell (AP_{out}) tells us about the train of input spikes (AP_{in}):

$$I_m(APs_{out}; APs_{in}) = I_{input} + \Sigma\, p(y)\, .\, \Sigma\, p(x|y)\, .\, \log_2 [p(x|y)]$$

where the input x is 1 when there is a spike and 0 otherwise, the output y is 1 when there is a spike and 0 otherwise, and the sums are over all x and y (ref. [S2], eqn. 4.12). In order to be counted as being triggered by an input spike, an output spike had to occur within a particular time window after the input spike. This time window was set to 18 ms based on post-stimulus time histograms of the experimental data (Figures S5A-S5C), and probability distributions were built for each recording, for each of four possible occurrences (x-y): (1) an output spike preceded by an input spike (1-1); (2) an output spike not preceded by an input spike ("0-1"); (3) no output spike when there was an input spike ("1-0"); (4) no output spike when there was not an input spike ("0-0"). Results of this calculation were divided by the time bin (3 ms) to get information rates in bits/sec. Applying this method gave information estimates that were almost identical to those calculated using the direct method, and the choice of information analysis method did not affect the energy efficiency curve (Figure S5D).

Mathematical model of thalamic relay cells

The mathematical model of thalamic relay cells was adapted from earlier models [S5, S6 and S7]. Briefly, the model LGN neuron consisted of a single compartment that included voltage-dependent currents described by Hodgkin-Huxley kinetics [S8]

$$C_m \frac{dV}{dt} = -\sum_j i_j - i_{Hold} - i_{Syn}$$

where C_m = 1 µF/cm^2 is the membrane capacitance, V is the membrane voltage (in mV), i_{Hold} is the injected current, i_{Syn} is the synaptic current and i_j are the intrinsic currents. The cell surface area was 1.52·10^{-4} cm^2, derived from the measured cell capacitance of 152±8 pF (mean±s.e.m., n=18) by assuming a specific capacitance of 1 µF/cm^2. All currents and conductances are subsequently reported per unit surface area (cm^2). Following Bazhenov and colleagues [S6], the intrinsic currents included a leak current i_L, a potassium leak current i_{KL}, an A-type potassium current i_A, a T-type low threshold calcium current i_T, an h-current i_h, a fast sodium current i_{Na} and a fast potassium current i_K. All the intrinsic currents had the same general form

$$i = g m^M h^N (V - E)$$

where for each current *i*, *g* is the maximal conductance, *m(t)* is the activation variable, *h(t)* is the inactivation variable, *E* is the reversal potential and *M* and *N* are the number of independent activation and inactivation gates.

The i_h current was given by

$$i_h = g_{\max} O \, (V - E_h)$$

with E_h = –43 mV [S9]. $g_{\max}$ = 0.0254 mS/cm^2 was set to match the average current recorded in our experiments in response to a 200 ms hyperpolarizing voltage-clamp pulse from –60 to –120 mV. The time dependence of the gating variable O was defined by

$$\frac{dO}{dt} = \frac{1}{\tau_O}(O_\infty - O)$$

with time constant $\tau_O = 1/\left[e^{(-14.59-0.086 \cdot V)} + e^{(-1.87+0.0701 \cdot V)}\right]$ (in msec) and steady-state variable $O_\infty = 1/\left[1 + e^{((V+75)/5.5)}\right]$ [S9].

The leak currents were given by

$$i_L = g_{\mathrm{L}}(V - E_L)$$

and

$$i_{KL} = g_{\mathrm{KL}}(V - E_K)$$

with E_L = –70 mV [S5]. E_K was set to match the effective potassium reversal potential used in the experiments E_K = –105 mV, while g_{L} = 0.025 mS/cm^2 and g_{KL} = 0.025 mS/cm^2 were manually adjusted to match both the average input resistance at the resting membrane potential (R_i = 149±28 MΩ in experiments, mean ± s.e.m., n=18; R_i = 145 MΩ in the model) and the resting membrane potential as recorded in experiments (V_{rest} = -76±2 mV in experiments, mean ± s.e.m., n=18; V_{rest} = –77.4 mV in the model).

The A-type potassium current was given by

$$i_A = g_A m^M h^N (V - E_K)$$

with M = 4 and N = 1. The time dependence for m and h was defined as for O, with

$$m_\infty = 1/\left[1 + e^{(-(V+60)/8.5)}\right]$$

$$\tau_m = 0.1 + 0.27/\left[e^{((V+35.8)/19.7)} + e^{(-(V+79.7)/12.7)}\right]$$

$$h_\infty = 1/\left[1 + e^{((V+78)/6)}\right]$$

and

$$\tau_h = 0.27/\left[e^{((V+46)/5)} + e^{(-(V+238)/37.5)}\right]$$

if $V < -63$ mV and $\tau_h = 5.1$ msec otherwise [S6 and S7].

The T-type calcium current was given by

$$i_T = g_T m^M h^N (V - E_T)$$

with M = 2 and N = 1. The time dependence for m and h was defined as for O, with

$$m_\infty = 1/\left[1 + e^{(-(V+57)/6.2)}\right]$$

$$\tau_m = 0.13 + 0.22/\left[e^{(-(V+132)/16.7)} + e^{((V+16.8)/18.2)}\right]$$

$$h_\infty = 1/\left[1 + e^{((V+83)/4)}\right]$$

and

$$\tau_h = 8.2 + \left[56.6 + 0.27 \cdot e^{((V+115.2)/5)}\right]/\left[1 + e^{((V+86)/3.2)}\right]$$

E_T is given by $E_T = RT/2F \cdot \log(\mathrm{Ca}_0^{2+}/\mathrm{Ca}^{2+})$ with F = 96489 C/mol the Faraday constant, R = 8.314 J mol^{-1} K^{-1} the gas constant, T = 309°K the temperature and Ca_0^{2+} = 2 mM the extracellular calcium concentration. The intracellular calcium dynamics were defined by

$$\frac{d\mathrm{Ca}^{2+}}{dt} = -\frac{1}{\tau_{\mathrm{Ca}}}\left(\mathrm{Ca}^{2+} - \mathrm{Ca}_i^{2+}\right) - A\, i_T$$

with Ca_i^{2+} = 2.4·10^{-4} mM, the baseline intracellular calcium concentration, and A = 5.18·10^{-5} mM cm^2 msec^{-1} µA^{-1}, a constant.

The fast sodium current was defined by

$$i_{Na} = g_{Na} m^3 h (V - E_{Na})$$

with E_{Na} = +90 mV adapted to match the experiments. The maximal conductance g_{Na} = 4.4 mS/cm^2 was set to match the peak current recorded in experiments in response to a 200 ms +40 mV depolarizing voltage step from the resting membrane potential. The time dependence for m and h was defined by

$$\frac{dx}{dt} = \alpha_x(1 - x) - \beta_x x$$

where x stands for either h or m and with [S10]

$$\alpha_m = 0.32\,\left[13.1 - V + V_{\text{shift}}^{\text{Na}}\right] / \left[e^{\left((13.1 - V + V_{\text{shift}}^{\text{Na}})/4\right)} - 1\right]$$

$$\beta_m = 0.28\,\left[V - V_{\text{shift}}^{\text{Na}} - 40.1\right] / \left[e^{\left((V - V_{\text{shift}}^{\text{Na}} - 40.1)/5\right)} - 1\right]$$

$$\alpha_h = 0.128\, e^{\left((17 - V + V_{\text{shift}}^{\text{Na}})/18\right)}$$

and

$$\beta_h = 4 / \left[1 + e^{\left((40 - V + V_{\text{shift}}^{\text{Na}})/5\right)}\right].$$

The fast potassium current was given by

$$i_K = g_K n^4 (V - E_K)$$

The maximal conductance g_K = 3.3 mS/cm^2 was set to match the steady-state current recorded in experiments in response to a 200 ms +40 mV depolarizing voltage step from the resting membrane potential. The time dependence for *n* was defined as for the sodium gating variables *m* and *h* with [S10]

$$\alpha_n = 0.032\,\left[15 - V + V_{\text{shift}}^{\text{K}}\right] / \left[e^{\left((15 - V + V_{\text{shift}}^{K})/5\right)} - 1\right]$$

and

$$\beta_n = 0.5\, e^{\left((10 - V + V_{\text{shift}}^{\text{K}})/40\right)}.$$

$V_{\text{shift}}^{\text{Na}}$ = –60.1 mV and $V_{\text{shift}}^{\text{K}}$= –62.5 mV were manually adjusted to allow the model to be depolarized to –55 mV without spontaneously spiking. These values slightly differ from the values in the model of ref. [S10] ($V_{\text{shift}}^{\text{Na}}$ = $V_{\text{shift}}^{\text{K}}$= –60 mV) and from the values in the model of refs. [S6] and [S7] ($V_{\text{shift}}^{\text{Na}}$ = $V_{\text{shift}}^{\text{K}}$= –63 mV). i_{Hold} = -2.05 µA/cm^2 was set in subsequent simulations so as to hold the model at –55 mV. For a cell surface area of $1.52 \cdot 10^{-4}$ cm^2, this corresponds to an injected current of ~310 pA, similar to experimentally measured values of 30-550 pA. g_A = 3 mS/cm^2 and g_T = 1.8 mS/cm^2 were set so that the model achieved an output frequency, when stimulated with the recorded synaptic conductance, similar to the average frequency observed in experiments with extracellular stimulation (5.3 Hz in the model, 4.1±0.8 Hz in the experiments, mean±s.e.m., n=18).

To determine the AMPA and NMDA synaptic conductance components generated by extracellular stimulation of retinal ganglion cell axons with the 125 second stimulus trains used in Figs. 3 to 5, we recorded the evoked currents in thalamic relay neurons held at -55 mV with and without bath application of the NMDAR blocker 50 µM D-AP5 (in the presence of 5 µM GABAzine). Currents were converted to conductance by dividing them by the holding potential (i.e. using a reversal potential of 0 mV). To determine the time course of the NMDA component, the conductance time course recorded in AP5 was subtracted from the total conductance time course. This procedure was repeated with three cells and the average AMPA (g_{AMPA}) and NMDA (g_{NMDA}) conductance time courses (Figure S4A) that were evoked by the input stimulation trains were used as inputs in the

simulations. To reproduce the dynamic-clamp experiments modulating the amplitude of the synaptic conductance, g_{AMPA} and g_{NMDA} were scaled by a gain factor varying between 0 and 12. Two batches of simulations were run, one with a linearized NMDA I-V relation, so that the total synaptic current varied linearly with voltage, as was used in the experiments, and one with a voltage-dependent NMDA conductance including Mg^{2+} block, to investigate whether our omission of this non-linearity in the dynamic clamp experiments had a significant effect on the results. In the first case, the synaptic current i_{Syn} is simply given by

$$i_{Syn} = -(g_{AMPA} + g_{NMDA})\,(V - E_{\text{excitatory}})$$

with $E_{\text{excitatory}}$ = 0 mV. To describe the NMDA non-linear I-V relation, we recorded the evoked currents in thalamic relay neurons generated by retinal ganglion cell stimulation during bath application of 1 µM NBQX (in the presence of 5 µM GABAzine) while holding the cell at different voltages (-74 mV to 36 mV in 10 mV steps). The recorded currents were then averaged across cells, and the voltage dependence was fitted by a function of the form $f(V) = a/(1 + b \cdot e^{-c \cdot V})$ with a, b and c as constants. i_{Syn} was then given by

$$i_{Syn} = -g_{\text{AMPA}}(V - E_{\text{excitatory}}) - g_{\text{NMDA}}\left(\frac{9.69}{1 + 0.1688\, e^{-0.0717\,V}}\right)(V - E_{\text{excitatory}})$$

where $g_{\text{AMPA}}(V\text{-}E_{\text{excitatory}})$ and $g_{\text{NMDA}}(V\text{-}E_{\text{excitatory}})$ describe the time course of the separate synaptic current components at -55 mV.

To calculate information transfer at the simulated synapse (Figure S4B-D), output spike trains were processed exactly as described for the experimental data. To calculate the metabolic cost incurred by the modelled cell, for Figures S4E and S4F the Na^+ component of i_{Syn} was integrated and converted to the corresponding ATP consumption per unit time, while for Figure S4G the same procedure was followed for i_{Na}, the Na^+ component of i_h and Ca^{2+} entry via i_T, and this was added to the ATP used on i_{Syn}. For i_{Syn}, the conductance was scaled by 7/13 (derived from the reversal potentials $E_{\text{excitatory}}$ = 0 mV, E_{Na} = +90 mV and E_K = -105 mV) and multiplied by V - E_{Na} to calculate the contribution of sodium ions. For i_h, the conductance was scaled by $(E_K - E_h)/(E_K - E_{Na})$ and multiplied by V - E_{Na} to isolate the contribution of sodium ions. For i_T, we assumed that each calcium ion is exchanged for 3 sodium ions [S11].

To convert currents and conductance back and forth between absolute amplitudes (measured) and amplitudes per surface area, we used the average cell surface area taken from membrane capacitance measurements obtained in experiments ($1.52 \cdot 10^{-4}$ cm^2; see above). Simulations were run using custom-written MATLAB scripts (The Mathworks, Natick MA). Differential equations were integrated using the built-in solver *ode15s* with an integration time step dt = 0.05 ms.

Tonic and burst modes of thalamic firing

Depending on their resting potential, relay neurons in the thalamus have two modes of firing: tonic mode (at -55 mV), where a single input spike tends to produce (at the most) one output spike, and burst mode (below -70 mV), where a single input spike may produce a burst of output spikes [S12], riding on a depolarising “calcium spike” (Figure S5E). Our analysis is restricted to cells firing in the tonic mode seen during alert wakefulness [S13 and S14]. Preliminary data from 3 cells (not shown) suggested that the same optimisation of information transmitted per energy used occurs during burst mode, however the synaptic energy efficiency may differ during less alert states or sleep, when synapses may renormalise their postsynaptic conductances and restore their energy supply [S15, S16 and S17]. A complication with carrying out this analysis in burst mode is the uncertainty in how information should be calculated: should bursts be treated as unitary events (in which case, they have been found to carry as much as three times the information of single spikes in tonic mode [S18]) or should the spikes within the bursts be treated as independent events, as we have done for tonic mode (in which case, bursts have been reported to contribute negatively to information transfer at the retinogeniculate synapse [S19])? A better understanding of which burst features are most relevant to coding at this synapse will be critical to assessing the energetic efficiency of information transmission in burst mode.

Effects of local inhibition at the retinogeniculate synapse

In vivo, the retinogeniculate synapse does not act in isolation, but in the presence of GABAergic modulation (both pre- and post-synaptically [S20, S21 and S22]). Inhibition evoked by retinal input has been suggested, in the cat, to generate net hyperpolarization of the LGN cell and control the transition from tonic firing to burst firing mode [S23]. We never saw this behaviour in our recordings (optic tract stimulation always evoked excitation followed by disynaptic inhibition, rather than net inhibition), possibly because of our use of rat rather than cat, or the absence of anaesthesia in our brain slice experiments, but inhibition might nevertheless shape the cells’ coding properties. Indeed, in principle, inhibition might improve the temporal precision of postsynaptic action potentials and thus increase the information encoded [S24].

We therefore performed the retinogeniculate experiments in the absence of gabazine, and found that approximately half of patch-clamped relay neurons had observable disynaptic inhibition, which tended to display short-term depression (Figure S1E). Surprisingly, we found that blocking the disynaptic inhibition in these cells by superfusing gabazine did not alter the firing frequency or information rate when presynaptic stimulation was applied (Figure S1F). Because gabazine also has no effect on EPSC size (Figure S1F), it appears that local inhibition does not significantly affect the energy efficiency of retinogeniculate transmission.

Correlations between inputs

In vivo, especially in sensory systems, it is likely that a set of postsynaptic neurons will receive inputs from presynaptic cells that are transmitting correlated information. An important future

line of work will be to assess the energetic efficiency of the whole population of retinal synapses onto the whole set of thalamic neurons. For example, the correlated activity of multiple cells may [S25], or may not [S26], increase the amount of information available to the visual cortex. When assessing how synaptic conductances are set to regulate information transfer and energy use, it will be interesting to investigate whether the conductance of each input to a postsynaptic cell is set independently, or whether account is taken of the correlations in information passing through spatially adjacent cells.

Relationship to earlier work

An earlier theoretical analysis claimed that the information transmitted at excitatory synapses is maximized per molecule of glutamate released [S27]. However, as has previously been noted (https://pubpeer.com/publications/23242311), this paper confused the two uses of the term entropy in information theory. If a signalling system can represent signals in many different ways (implying a high signal entropy), then it can transmit a large amount of information. However, if there is a lot of random noise in the signal then there will be a large noise entropy. Unfortunately the calculations in ref. [S27] maximise the noise entropy (by maximising the variations of postsynaptic current generated by random opening and closing of ion channels) - they do not maximise the signal entropy. Thus, the claim [S27] that the observed number of glutamate molecules maximises the signal information content is entirely the opposite of what their analysis does, which is to maximise the noise.

SUPPLEMENTAL REFERENCES

S1. Strong, S.P., Koberle, R., de Ruyter van Steveninck, R., and Bialek, W. (1998). Entropy and Information in Neural Spike Trains. Phys. Rev. Lett. *80*, 197-200.

S2. Dayan, P., and Abbott, L.F. (2001). Theoretical Neuroscience (Cambridge, Massachusetts: The MIT Press).

S3. Shannon, C.E. (1948). A mathematical theory of communication. Bell System Tech. J. *27*, 379-423.

S4. Levy, W.B., and Baxter, R.A. (1996). Energy efficient neural codes. Neural Comput. *8*, 531-543.

S5. McCormick, D.A., and Huguenard, J.R. (1992). A model of the electrophysiological properties of thalamocortical relay neurons. J. Neurophysiol. *68*, 1384-1400.

S6. Bazhenov, M., Timofeev, I., Steriade, M., and Sejnowski, T.J. (1998a). Cellular and network models for intrathalamic augmenting responses during 10 Hz stimulation. J. Neurophysiol. *79*, 2730-2748.

S7. Bazhenov, M., Timofeev, I., Steriade, M., and Sejnowski, T.J. (1998b). Computational models of thalamocortical augmenting responses. J. Neurosci. *18*, 6444-6465.

S8. Hodgkin, A.L., and Huxley, A.F. (1952). A quantitative description of membrane current and its application to conduction and excitation in nerve. J. Physiol. *117*, 500-544.

S9. Huguenard, J.R., and McCormick, D.A. (1992). Simulation of the currents involved in rhythmic oscillations in thalamic relay neurons. J. Neurophysiol. *68*, 1373-1383.

S10. Traub, R.D., and Miles, D. (1991). Neuronal networks of the hippocampus (Cambridge: CUP).

S11. Attwell, D., and Laughlin, S.B. (2001). An energy budget for signaling in the grey matter of the brain. J. Cereb. Blood. Flow Metab. *21*, 1133-1145.

S12. Scharfman, H.E., Lu, S.M., Guido, W., Adams, P.R., and Sherman, S.M. (1990). N-methyl-D-aspartate receptors contribute to excitatory postsynaptic potentials of cat lateral geniculate neurons recorded in thalamic slices. Proc. Natl. Acad. Sci. U.S.A. *87*, 4548-4552.

S13. Coenen, A.M., and Vendrik, A.J. (1972). Determination of the transfer ratio of cat's geniculate neurons through quasi-intracellular recordings and the relation with the level of alertness. Exp. Brain Res. *14*, 227-242.

S14. Livingstone, M.S., and Hubel, D.H. (1981). Effects of sleep and arousal on the processing of visual information in the cat. Nature *291*, 554-561.

S15. Maret, S., Faraguna, U., Nelson, A.B., Cirelli, C., and Tononi, G. (2011). Sleep and waking modulate spine turnover in the adolescent mouse cortex. Nat. Neurosci. *14*, 1418-1420.

S16. Vyazovskiy, V.V., Cirelli, C., Pfister-Genskow, M., Faraguna, U., and Tononi, G. (2008). Molecular and electrophysiological evidence for net synaptic potentiation in wake and depression in sleep. Nat. Neurosci. *11*, 200-208.

S17. Harris, J.J., Jolivet, R., and Attwell, D. (2012). Synaptic energy use and supply. Neuron *75*, 762-777.

S18. Reinagel, P., Godwin, D., Sherman, S.M., and Koch, C. (1999). Encoding of visual information by LGN bursts. J. Neurophysiol. *81*, 2558-2569.

S19. Reinagel, P., and Reid, R.C. (2000). Temporal coding of visual information in the thalamus. J. Neurosci. *20*, 5392-5400.

S20. Turner, J.P., and Salt, T.E. (1998). Characterization of sensory and corticothalamic excitatory inputs to rat thalamocortical neurones in vitro. J. Physiol. *510*, 829-843.

S21. Chen, C., and Regehr, W.G. (2000). Developmental remodeling of the retinogeniculate synapse. Neuron *28*, 955-966.

S22. Chen, C., and Regehr, W.G. (2003). Presynaptic modulation of the retinogeniculate synapse. J. Neurosci. *23*, 3130-3135.

S23. Wang, X., Hirsch, J.A., and Sommer, F.T. (2010). Recoding of sensory information across the retinothalamic synapse. J. Neurosci. *30*, 13567-13577.

S24. Butts, D.A., Weng, C., Jin, J., Yeh, C., Lesica, N., Alonso, J., and Stanley, G.B. (2007). Temporal precision in the neural code and the timescales of natural vision. Nature *449*, 92-96.

S25. Dan, Y., Alonso, J.M., Usrey, W.M., and Reid, R.C. (1998). Coding of visual information by precisely correlated spikes in the lateral geniculate nucleus. Nat. Neurosci. *1*, 501-507.

S26. Meytlis, M., Nichols, Z., and Nirenberg, S. (2012). Determining the role of correlated firing in large populations of neurons using white noise and natural scene stimuli. Vision Res. *70*, 44-53 .

S27. Savtchenko, L.P., Sylantyev S., and Rusakov D.A. (2013). Central synapses release a resource-efficient amount of glutamate. Nat. Neurosci. *16*, 10-12.
